# Genome-Guided Metabolomic Profiling of Peptaibol-Producing *Trichoderma*

**DOI:** 10.3390/ijms26125599

**Published:** 2025-06-11

**Authors:** Arseniy A. Sinichich, Danil V. Krivonos, Anna A. Baranova, Mikhail Y. Zhitlov, Olga A. Belozerova, Vladislav A. Lushpa, Andrey V. Vvedensky, Marina V. Serebryakova, Anastasia I. Kalganova, Arsen M. Kudzhaev, Yuri A. Prokopenko, Sofia S. Sinelnikova, Ekaterina A. Trusova, Sergey I. Kovalchuk, Elena N. Ilina, Stanislav S. Terekhov, Vera A. Alferova

**Affiliations:** 1 Shemyakin-Ovchinnikov Institute of Bioorganic Chemistry, Miklukho-Maklaya 16/10, 117997 Moscow, Russia; asinichich@yandex.ru (A.A.S.); anjabaranowa@list.ru (A.A.B.); droplbox38@gmail.com (M.Y.Z.); o.belozyorova@gmail.com (O.A.B.); lushpa1696@gmail.com (V.A.L.); kalganovanas@ibch.ru (A.I.K.); kudzhaev_arsen@mail.ru (A.M.K.); tetrahydrofuran@mail.ru (Y.A.P.); sinelnikowa.sofia@yandex.ru (S.S.S.); katatrusova532@gmail.com (E.A.T.); xerx222@gmail.com (S.I.K.); 2 Department of Chemistry, Lomonosov Moscow State University, Kolmogorova 1, 119992 Moscow, Russia; 3 Moscow Center for Advanced Studies, Kulakova Str. 20, 123592 Moscow, Russia; danil01060106@gmail.com; 4 Research Institute for Systems Biology and Medicine (RISBM), 18 Nauchniy Proezd, 117246 Moscow, Russia; vvedenskiia@sysbiomed.ru (A.V.V.); ilinaen@gmail.com (E.N.I.); 5 Belozersky Institute of Physico-Chemical Biology, Lomonosov Moscow State University, 1-40 Leninskie Gory, 119991 Moscow, Russia; mserebr@mail.ru

**Keywords:** *Thichoderma* sp., SF4-type peptaibols, biosynthesis, trichorozins, antibacterial, cytotoxic

## Abstract

Peptaibols are linear fungal peptides featuring α,α-dialkylated amino acids (e.g., α-aminoisobutyric acid (Aib), isovaline (Iva)) and characteristic C-terminal alcohol groups. Despite their promising antibacterial and antiplasmodial activities, detailed biosynthetic studies remain limited. A genome-guided study of the fungus *Trichodema* sp. SK1-7, isolated from decaying wood, revealed the production of previously described trichorozin IV (**1**), along with novel SF4-type peptaibol **2** (trichorozin V). The structures of these compounds were elucidated through MS analysis, NMR study and advanced Marfey’s method. The genome of *Trichoderma* sp. SK1-7 harbors two PKS-NRPS hybrid gene clusters containing 14 and 18 adenylation domains. Analysis of the modular architecture suggested that trichorozins are synthesized by a 14-module protein via a module skipping mechanism. Genome mining revealed several types of short peptaibol synthase architectures (10–14 adenylation domains) across various *Trichoderma* species, accompanied by similar long peptaibol synthases. Furthermore, putative Aib/Iva biosynthesis machinery in *Trichoderma* was identified, showing specific architectures potentially involved in regulating peptaibol biosynthesis. Feeding experiments demonstrated that peptaibol production depends on the ratio of Iva/Aib. The isolated compounds exhibited moderate antibacterial and cytotoxic activities along with a synergistic effect when combined with membrane-targeting antibiotics. Our findings suggest that genome-guided approaches hold promise for further development of peptabiotics with a wide range of applications, including antibiotic adjuvants.

## 1. Introduction

Peptaibols (stands for peptide-Aib-alcohol) and more generally peptaibiotics (peptaibols, lipopeptaibols, lipoaminopeptaibols) represent a distinct class of non-ribosomally synthesized compounds, characterized by noncanonical α,α-dialkylated amino acids (mostly aminoisobytyric acid, AIB), an acylated N-terminus and C-terminal 1,2-aminoalcohol. Peptaibols form special *α*-helix and *β*-bend patterns, which endow them with permeabilizing abilities [1,2]. Notable resistance to proteolytic degradation along with a wide range of biological activity make peptabiotics compelling candidates for pharmaceutical and agricultural applications. Several very promising compounds were isolated from natural sources. For example, peptaibols from marine fungus *Stephanonectria keithii* LZD-10-1 exhibit high antimicrobial activity and in vivo efficacy against staphylococcus infection [3], lipopeptaibols texenomycins from *Mariannaea elegans* showed considerable antifungal activity [4], peptaibols from *Trichoderma guizhouense* demonstrated cytotoxic properties [5], peptaibols from *Trichoderma longibrachiatum* showed synergistic effects in plant–microbe signaling interactions [6], peptaibols from *Simplicillium obclavatum* showed significant activity against the tobacco and tilapia pathogens [7] and peptaibols from *Emericellopsis* sp. possessed herbicidal activity [8]. Moreover, the antiplasmodial activity of peptaibols attracts significant attention [9,10]. Membrane-permeabilizing properties of peptaibols can also be utilized for carrier peptides in oligonucleotide delivery [11,12,13,14,15].

**Figure 1:**
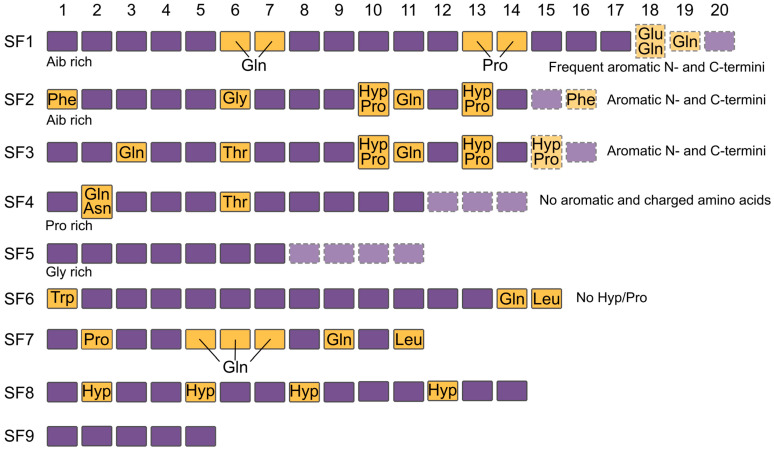
Structural features of peptaibol subfamilies. Conserved positions are indicated with yellow, and variable positions are highlighted with violet. Chain length variations are marked with dashed lines. Hyp—hydroxyproline.

Initially, peptaibols were classified as “long” (17–20 residues), “short” (usually 11–16 residues) and “lipopeptaibols” (N-acylated with long fatty acids) [1]. Alternative classification [16], including sequence similarities and structural features, divides peptaibols into nine subfamilies, SF1-SF9 (Figure 1). Since 2001, when this classification was introduced, a lot of alternative peptaibol sequences were discovered, but in the absence of more accurate classification, SFs remain a practical classification tool [1].

Despite high interest in peptaibiotics for drug development, data on the exact structure and biosynthetic origin of peptaibols is rather scarce. *Trichoderma* spp. is the most abundant peptaibol-producing organism. Currently, only 90 complete genome sequences are available for *Trichoderma* sp. in MycoCosm [17] (https://data.jgi.doe.gov/refine-download/mycocosm, accessed on 31 March 2025). Moreover, data on peptaibol biosynthetic origin is not readily available; the MiBIG database provides only the biosynthetic gene cluster (BGC) of emerimicin (BGC0002555) [18]. Recent studies reveal the importance of the exact structure of the compound on its biological properties [19]. Nonetheless, a lot of previous studies identify only the amino acid sequence of the produced compounds. This approach neither provides stereoconfiguration of the peptide, nor distinguishes Leu/Ile and Val/Iva. In-depth analysis of structure–activity relationships in peptaibol families enables the rational design of effective drug candidates, as recently illustrated by the development of peptaibol-based analogues, effective against multi-drug resistant bacteria [20].

In this work, we report the genomic and metabolic characterization of peptaibol-producing *Trichoderma* sp. SK1-7, taxonomically distinct from other species. Detailed structural characterization of major peptaibols revealed a novel trichorozin IV homologue (trichorozin V). Genome mining and peptaibol–synthase architecture analysis display various gene cluster architectures of SF4-peptaibol synthases and co-production with SF1-type peptaibols, affected by the Aib/Iva ratio in feeding experiments. These findings suggest plausible attenuation of long peptaibols by their shorter counterparts. Short peptaibols, exhibiting moderate cytotoxic and antimicrobial activity, were able to potentiate membrane-active compounds against ESKAPE pathogens.

## 2. Results

### 2.1. Antimicrobial Activity of Strain SK1-7 Is Associated with the Production of 11-Residue Peptaibols

**Figure 2:**
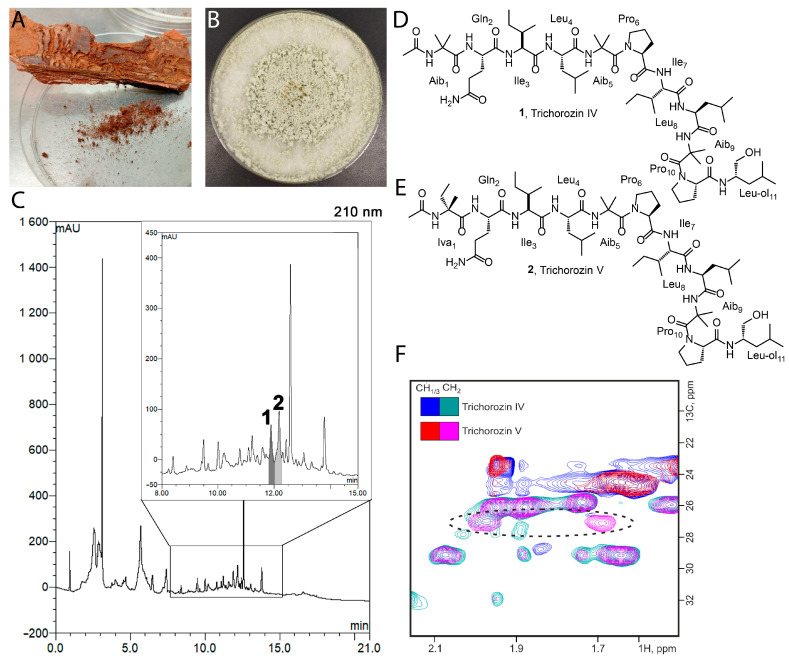
Isolation and activity-guided fractionation of the producing strain. (**A**) A sample of spruce bark infested with bark beetle (*Ips typographus*). (**B**) Morphology of the isolated active strain SK1-7. (**C**) HPLC-DAD Chromatogram of Sk1-7 mycelium extract, 210 nm. Inlay—zoomed from 8 to 15 min, collected fractions in grayscale—**1** and **2**. Injected volume—20 μL. (**D**) The structure of the peptide **1**. (**E**) The structure of the peptide **2**. Three-letter designations of amino acid residues are introduced in the figure. (**F**) Fragment of superposition of ^1^H, ^13^C-HSQC NMR spectra of the **1** and **2** samples. Signals of CH-/CH_3_-groups are shown in blue/red, and CH_2_-groups in green/violet. Signals of an additional CH_2_-group present in compound **2** are highlighted in an oval.

The inner part of spruce bark that was fed on by the bark beetle typographus (*Ips typographus*) was studied. The untreated spruce logs had been lying outdoors for 1 month in the village of Khombus-Batyrevo, Chuvash Republic, Russian Federation (55.293515 N, 47.088331 E). The average temperature in October 2022 was +6 °C with 82% humidity. Bark samples were stored in sterile containers. Preliminary bark samples were washed with sterile water and suspended for inoculation (Figure 2A). The inoculum was spread on 9 × 9 cm Petri dishes with solid nutrient medium brain heart infusion (with nystatin 50 μg/mL), potato dextrose (with tobramycin 25 μg/mL), nutrient agar (with nystatin 50 μg/mL) and nutrient agar (with nystatin 50 μg/mL and nalidixic acid 30 μg/mL). The dishes were incubated at 28 °C under aerobic conditions for 10 days to ensure the isolation of the less abundant bacteria and fungi, since they require a longer period to form colonies. As the colonies grew, they were transferred and purified on potato-dextrose agar (fungi) or nutrient agar (bacteria). The strains were stored in glycerol (20%, *v*/*v*) at −87 °C. In total, 46 microbial isolates were isolated.

The isolates were screened using the agar diffusion method against a wide range of test microorganisms; the strain SK1-7 (Figure 2B) exhibited antimicrobial activity against Gram-positive bacteria (*Bacillus subtilis* ATCC 6633) and the hypersensitive *Escherichia coli* strain (*E. coli lptD*^mut^) with a permeable outer membrane.

Activity-guided fractionation revealed two major components with antimicrobial activity (**1**, **2**, Figure 2C). High-resolution LC-MS analysis of isolated fractions performed in positive ion mode identified two main components, **1** and **2**, exhibiting molecular ions at *m*/*z* 1189.7922 [M+H]^+^ and 1203.8071 [M+H]^+^, corresponding to the molecular formulas C_59_H_104_N_12_O_13_ and C_60_H_106_N_12_O_13_, respectively. MS/MS spectra acquired in HCD mode showed highly similar fragmentation patterns for both compounds. For compound **1**, key fragment ions were detected at *m*/*z* 256.13, 369.21, 482.29 and 567.39. In the spectrum of compound **2,** analogous key fragment ions were observed at *m*/*z* 270.15, 383.23, 496.31 and 581.36, each shifted by +14.02 Da. This consistent mass difference suggests that compound **2** has one additional CH_2_ group compared to compound **1**, indicating that the two molecules are structural homologues. Complete LC-MS and MS/MS data are provided in the Supplementary Materials (Figures S1 and S2). To resolve the precise structural differences, both compounds were subsequently analyzed by NMR spectroscopy.

In the case of the **1** sample, the 2D ^1^H,^1^H-TOCSY and ^1^H,^13^C-HSQC spectra allowed for the identification of signals corresponding to the following amino acid residues (hereinafter referred to as aa): Gln (1), Leu (2), Ile (2), Leu-ol (1), Pro (2) and Aib (2). The presence of the additional Ala-αMet was inferred from the combined analysis of the ^1^H,^13^C-HSQC and ^1^H,^13^C-HMBC spectra. By examining the determined chemical shifts of the protons associated with the nitrogen atom, we were able to ascertain the chemical shifts of the NH groups, with the exception of those corresponding to prolines and the initial Ala-αMet residue.

The NOE signals H_α_-H_α+1_ facilitated the identification of the following molecular fragments: Aib-Gln-Ile-Leu-Aib and Ile-Leu-Aib. The immediate neighbors of both prolines were established through cross-peaks in the NOESY spectra, which enabled us to assign the complete polypeptide chain as follows: Ac-Aib-Gln-Ile-Leu-Aib-Pro-Leu-Aib-Pro-Leu-ol. Additionally, the methyl signal associated with the acetyl group was identified through the analysis of the ^1^H,^13^C-HSQC and ^1^H,^13^C-HMBC NMR spectra (Supplement S1). The chemical shift values for the atoms in **1** are presented in Table S1, while the secondary structure is illustrated in Figure 2D. Comparison with published data reveals that the planar structure of **1** is identical to previously characterized peptaibol trichorozin IV, belonging to the SF4 family of short peptaibols.

In the case of **2**, the assignment of signals was conducted using the aforementioned method (Figure 2E). Notably, additional signals were identified in the ^1^H,^13^C-HSQC and ^1^H,^13^C-HMBC spectra of **2**, which correspond to a CH_2_- group (Figure 2F, Supplement S1). This additional group is situated in the first Aib residue, between the Cα atom and one of the associated methyl groups. The resulting amino acid is isovaline. Therefore, the complete sequence of sample **2** is as follows: Ac-Iva-Gln-Ile-Leu-Aib-Pro-Leu-Aib-Pro-Leu-ol. The structure of **2**, named trichorozin V, is depicted in Figure 2E, while the chemical shift values of the NMR signals are provided in Table S2. The compounds with similar composition were previously detected by mass spectrometry [21]. Interestingly, the compound with a similar sequence to **2**, trichofumin B from *Trichoderma* sp. HKI 0276, was shown to contain only Leu residues by derivatization [22].

The advanced Marfey’s analysis of **1** confirmed the l-configuration for Glu/Gln, Pro, Ile and Leu residues. In **2,** hydrolizate amino acid residues also exhibited l-configuration, except for Iva, which was determined to have a d-configuration.

### 2.2. Complete Genome Sequencing Reveals Distinct Taxonomical Position of Trichoderma sp. SK1-7

The total length of the assembled genome is 41.39 Mb represented by 35 contigs; there are 10 mismatches (N letters) in the resulting assembly. N50 is 3125370 and L50 is 5. GC % of the genome is 47.25 %. The mitochondrial chromosome is assembled as a ring of 34921 base pairs. The mitochondrial DNA coverage is about 15 times higher than the rest of the genome. When the mitochondrial DNA sequence was aligned using blastn in nr/nt, the closest entries belonged to *Trichoderma pyromidale* (Query coverage = 86% and Per. Ident = 94.39%) and *Trichoderma afrohrozianum* (Query coverage = 75% and Per. Ident = 98.52%). The mitochondrial DNA alignment itself is fragmented (visualization in Supplement S1 and Figures S1–S3). The *T. pyromidale* genome reference assembly is not available in the NCBI Genomes database, and the *T. afrohrozianum* Th6 reference assembly possesses different assemblies from the one obtained in this work. The *T. afrohrozianum* Th6 assembly is 41.6 Mb in size with GC composition equal to 46.5 %.

**Figure 3:**
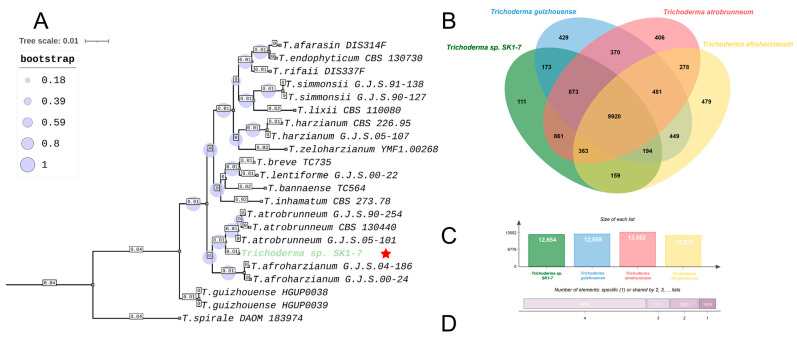
(**A**) Phylogenetic tree constructed by maximum likelihood mode based on ITS + *tef1 + rpb2* concatenate; *T. spirale* DAOM was used as an outgroup, T. hombus SK1-7 is marked with green font and red star. (**B**) Venn diagram for *T. hombus* SK1-7 and closely related species. (**C**) Number of protein clusters in different species. (**D**) Number of protein clusters specific for 2, 3, 4 species and unique protein clusters (1).

A total of 513 samples were used for the complete phylogenetic analysis of the *tef1* + *rpb2* concatenate, and 351 samples were used for the complete phylogenetic analysis of the ITS + *tef1* + *rpb2* concatenate. We have indicated the GenBank IDs of all sequences used in the phylogenetic analysis in Table S3. Phylograms were constructed both for all available *Trichoderma* strains that were used for analysis (Supplemental Figures S1–S3) and only for the most phylogenetically similar species to SK1-7. Thus, 27 samples for the *tef1*-*rpbA* tree and 22 samples for the ITS + *tef1* + *rpb2* tree were used to construct such trees. Based on the phylograms obtained, *Trichoderma* sp. SK1-7 forms a sister clade with strains of the species *T. atrobrunneum*. This indicates a new taxon, and the similar formation of a sister clade of *Trichoderma* sp. SK1-7 with *T. atrobrunneum* can be traced in the phylograms constructed both with tef1 + rpb2 concatenate (Figure S4) and with ITS + *tef1* + *rpb2* (Figure 3A). We propose the new taxon name *Trichoderma hombus* for the strain SK1-7 on the basis of the strain’s isolation location.

Full reference genome assemblies of *T. guizhouense*, *T. atrobrunneum* and *T. afroharzianum* were taken for comparative genomic analysis (Figure 3B–D). The genome of the closely related species *T. pyramidale* is not available in the NCBI Genomes database at the time of the study, so this species was not used for comparative genomic analysis. A total of 15,546 clusters of orthologs were found for the four species; among them, 5211 unique clusters occur only for a single species. In total, 9920 clusters of orthologs were found in all four analyzed species. Further, 111 clusters are unique to *T. hombus* SK1-7, 173 clusters are common only to *T. guizhouense* and *T. hombus*, 159 are common only to *T. afroharzianum* and *T. hombus* and 861 clusters are common to *T. hombus* and *T. afroharzianum*.

Out of 111 clusters, according to the molecular functions, 18.75% associated with oxidoreductase activity (GO:0016491), 12.50% associated with hydrolase activity (GO:0016787), 12.50% associated with transferase activity(GO:0016740), 12.50% associated with transporter activity (GO:0005215), 12.50% associated with molecular function (GO:0003674), 6.25% associated with ion binding (GO:0043167), 6.25% associated with binding (GO:0005488), 6.25% associated with nucleic acid binding (GO:0003676), 6.25% associated with nucleic acid binding (GO:0001882) and 6.25% associated with nucleoside binding (GO:0000166). A total of 62 out of 111 orthology clusters have GO annotation and the rest have an undescribed function. A complete list of ortholog clusters is provided in the Supplementary Materials.

### 2.3. Bioinformatic Analysis Provides Insights into the Biosynthetic Origin of SF4-Type Peptaibols

**Figure 4:**
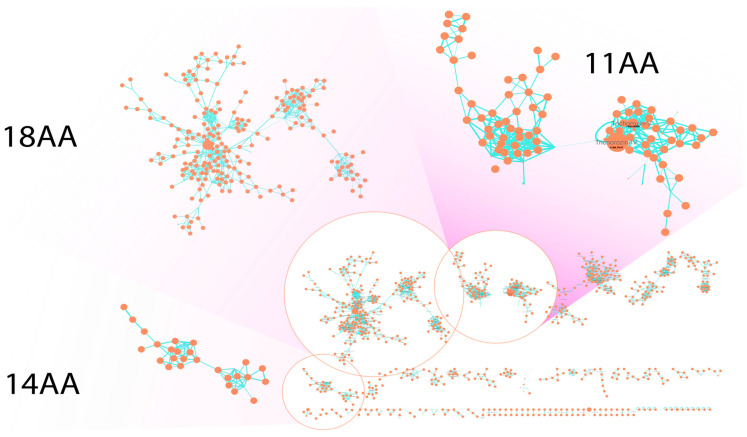
Feature-based molecular network (FBMN) of total peptabiolic fraction. Each node corresponds to the mass observed in the spectrum, the size of the node reflects the overall intensity and the edge width is proportional to the cosine score (a value that evaluates the similarity of the fragmentation spectra). Right bottom corner—overview on FBMN, 11 AA—cluster of peptaibols containing 11 amino acids, 14 AA—cluster of peptaibols containing 14 amino acids, 18 AA—cluster of peptaibols containing 18 amino acids (trichokindin-like). Trichorozin IV and Trichorozin V are annotated in 11AA.

Detailed study of biosynthesis of short SF4-type peptaibols was described only for the *tex2* gene with 14 modules from *Trichoderma virens* Gv29-8 [21]. The suggested module skipping mechanism was further confirmed by deletion in *T. virens*, *T. reesei* QM9414 and *T. atroviride* [23]. Generally, *Trichoderma* sp. strains produce complex mixtures of peptaibols; 11AA products are more frequently detected than 14AA-variants [24]. The production of 11AA and 14AA products was detected in several studies, including a biocontrol agent survey [25], but both metabolomic and genomic characteristics are rarely available for short peptaibol families. Therefore, we decided to analyze biosynthetic gene clusters in the *Trichoderma* sp. SK1-7 genome. AntiSMASH analysis revealed two large PKS-NRPS hybrid clusters, harboring two peptaibol synthases with 14 adenylation domains (named *tho1*, Table S4) and 18 adenylation domains (*tho2*, Table S5). To corroborate the production of long peptaibols, we analyzed total peptaibolic fractions of *Trichoderma* sp. SK1-7 with feature-based molecular networking and revealed a cluster of 18 residue-containing peptaibols (Figure 4). Analysis of mass fragmentation for two major components of the MS network cluster (**3** with [M+2H]^2+^ ion at *m*/*z* 881.5529 and **4** with [M+2H]^2+^ ion at *m/z* 874.5435) revealed the amino acid sequence Ac-Aib-Ser-Ala-Aib-Iva-Gln-Iva-Leu-Aib-Ala-Aib/Iva-Aib-Pro-Leu-Aib-Aib-Gln-Valol, characteristic for SF1-type peptaibols trichokindins previously isolated from *T. harzianum* [26] (Figures S5 and S6). Minor components of the cluster included trichokindin-like peptaibols (Figure S7) with additional Aib/Iva substitutions. Moreover, the molecular network revealed production of 14-residue peptaibols by *Trichoderma* sp. SK1-7 (Figure 4).

**Figure 5:**
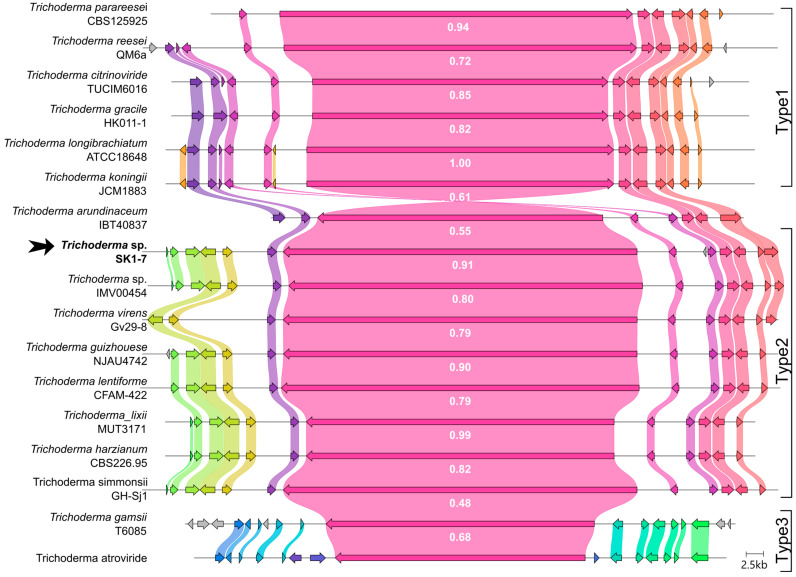
Comparison of the short (10–14 adenylation domains) peptaibol synthase-containing BGCs (Table S5), generated using the clinker tool (https://cagecat.bioinformatics.nl/, accessed on 30 April 2025) [30]. Homologous genes are color-coded, and labels indicate the corresponding protein identities.

In genera other than *Trichoderma,* 11-residue peptabiotics are shown to be formed by NRPS with 11 adenylation modules; for example, Bmt-containing peptabiotic tolypocladamide H, isolated from entomopathogenic fungus *Tolypocladium inflatum* [27]. To analyze the genomic context and frequency of short and long peptaibol synthase co-existence, extensive literature and genome mining for *Trichoderma* complete genome sequences harboring short (10–14 adenylation domains) synthases was performed. As 15-residue peptaibols generally bear low sequence similarity with SF4 type compounds and are produced by synthases with alternative architecture [28,29], they were excluded from this analysis. The related *Trichoderma* species were annotated using AUGUSTUS and analyzed with AntiSMASH. Presence of the relevant clusters was validated manually; the resulting set of strains is provided in Table S6. Interestingly, all the selected genomes contained long peptaibol synthase in addition to the short one, with only slight differences in genomic context (Figure S7). A comparison of BGC architecture revealed three types of short peptaibol synthases (Figure 5), with *tho1* BGC in *Trichoderma* sp. SK1-7 belonging to the most abundant type 2.

**Figure 6:**
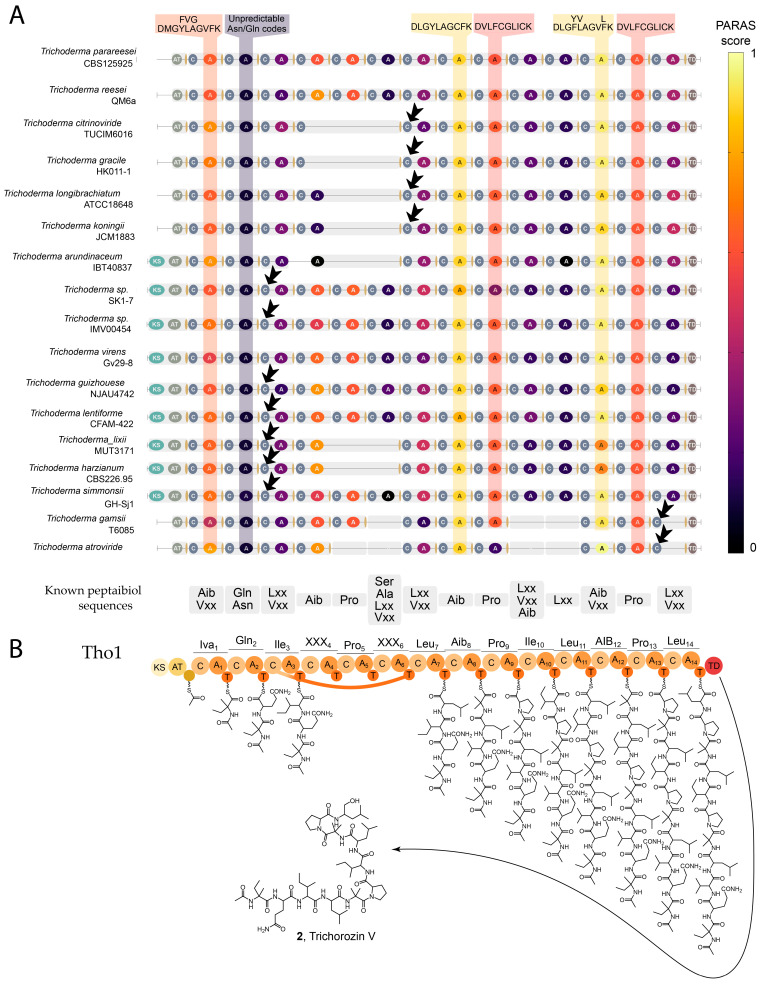
Analysis of *tho1* BGC. (**A**) Comparison of the short (10–14 adenylation domains) peptaibol synthase domain architecture (Table S6). Adenylation domains are color-coded according to the PARAS score of substrate specificity prediction. Black arrows indicate condensation domains with HHxxxDG active site consensus sequence deviations. (**B**) Proposed scheme of SF4 peptaibol trichorozin V (**2**) biosynthesis.

Previous works showed that adenylation domain phylogenetic analysis [28,29] and signature sequence comparison [21,23] are limited in the prediction of substrate specificity for peptaibol synthases. Therefore, we probed recently developed machine-learning specificity predictor PARAS for the analysis of the module architecture [31] (Figure 6A). PARAS-score mapping was found to be useful for peptaibol synthase gene architecture alignment. Some of the gaps can be attributed to the low quality of sequences, but the general trend of M4-M6 gaps suggests that module loss can be an alternative mechanism for short peptaibol structural diversification. A recent report on [29] the co-production of short (11 and 14AA peptaibols) and long (15AA) peptaibols suggests that 15-residue products are the result of module losses in *Trichoderma endophyticum* MMSRG85.

Alignment of condensation domains revealed an unusual HHxxxDA motif instead of the canonical HHxxxDG in the active site of the C3 domain in Tho1. The HHxxxDA motif was also identified in module 3 condensation domains in most of type 2 peptaibol synthases (Figure 6A) and module 7 of type 1 synthases. Notably, modules 3 and 7 flank the skipped fragment in 14-module synthases. Module skipping is extremely rare in natural NRPS systems. It was first described for myxochromides S biosynthesis [32]. In the case of *mch* BGC, both skipped and upstream condensation domains have alterations in the classic HHxxxDGxS motif. The C4 domain in the skipped module harbors the HHxxxDAWS active site sequence, whereas upstream C5 has conservative histidine substitution (HQxxxDPAS) [32]. This motif is reported in hybrid [33,34] and stuttering NRPS [35] and associated with diffusible substrate acceptance. The location of this substitution in peptaibol synthases suggests that C-domains can facilitate chain transfer during module skipping. Interestingly, type 3 clusters contained condensation domain alterations in the last module (Table S7). The analysis of the domain architecture allowed us to suggest the scheme of trichorozin V (**2**) biosynthesis via skipping of modules 4, 5 and 6 in Tho1 (Figure 6B).

Due to the production of a wide variety of closely related peptides, the majority of studies rely on mass-spectrometry-based identification of peptide sequences. Moreover, some of the previously described compounds were shown to contain d-configured amino acids; e.g., d-Iva in hypomurocins A, B [36] and zeramicins IIB [37]. NRPS, introducing d-configured amino acids, usually contain epimerization domains in corresponding modules, but in the biosynthesis of emerimicins, d-Iva was shown to be activated by the adenylation domain [18]. Therefore, peptaibol-producing Trichoderma are capable of synthesizing d-Iva.

**Figure 7:**
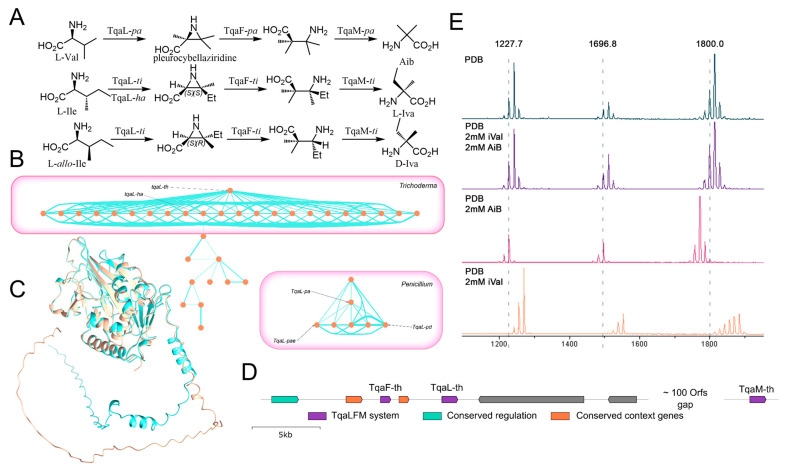
(**A**) Biosynthetic origin of Aib and Iva, synthesized by various TqaLFM systems. (**B**) Fragment of SSN generated for TqaL-th sequence. Each node represents TqaL-th homologue; edge width represents protein identity. Reported TqaLs are marked. (**C**) Alignment of TqaL-th (this work, orange) and Tqal-ha (XP_013945817.2, teal) models generated by AlphaFold3 [42] and aligned with ChimeraX. Alignment RMSD and AlphaFold PAE are provided in Figure S10. (**D**) Genomic context of TqaLFM-th in *Trichoderma* sp. SK1-7: conservative genes revealed by GND analysis highlighted with color. (**E**) MALDI-TOF spectra of total peptaibolic fraction, obtained from long-term (24 days) cultivation of *Trichoderma* sp. SK1-7 under different cultivation conditions. [M+K]^+^ pseudomolecular ion peaks are observed. The highlighted masses correspond to **1** (calc. [M+K]^+^ 1227.7482), **3** (calc. [M+K]^+^ 1800.0445 *m*/*z*) and unidentified 14-residue peptaibol.

**Figure 8:**
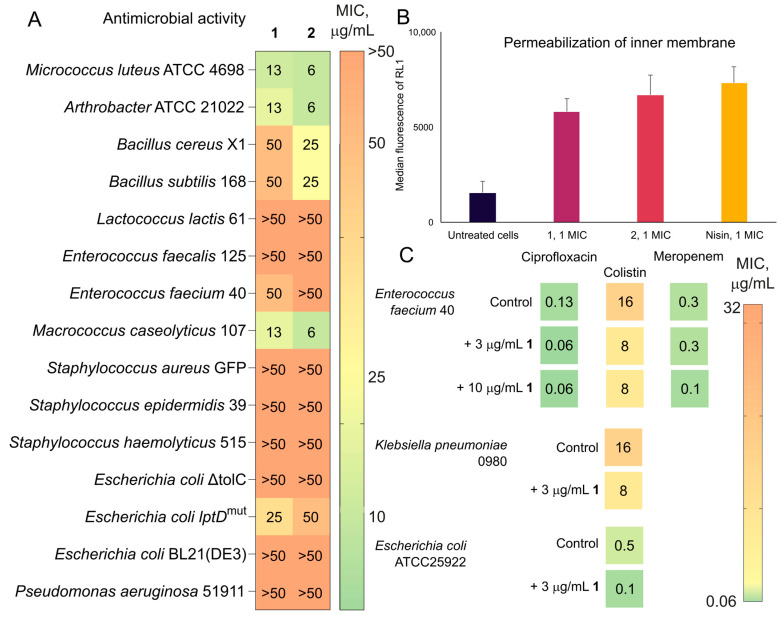
Biological evaluation of trichorozins IV (**1**) and V (**2**). (**A**) Heat map of MICs of **1** and **2** against Gram-positive and Gram-negative bacteria. (**B**) The effect of **1** and **2** on the permeabilization of the *M. luteus* inner membrane as measured by SYTOX red dead stain fluorescence, with nisin used as a positive control. (**C**) Synergistic effect of addition of **1** to the media on antimicrobial activity of colistin, meropenem and ciprofloxacid against ESKAPE pathogens, measured using the broth microdilution method in MHB.

Fungi are known to produce α,α-dialkylyated amino acid Aib and various mixtures of d- and l-Iva [38]. The biosynthetic origin of these amino acids remained unclear until recently. First, Aib was shown to be produced by *Penicillium arizonense* from l-valine via radical-mediated aziridine formation by Fe(II)/α-ketoglutarate (αKG)-dependent oxygenase (aziridinase) TqaL-*pa*. Subsequent ring-opening hydrolysis by a haloalkanoic acid dehalogenase-like protein TqaF-*pa* is followed by oxidative decarboxylation by a non-heme iron enzyme TqaM-*pa* [39] to yield Aib (Figure 7A). Later, dl-Iva was shown to also be produced by TqaLFM-ti from l-Ile [40]. TqaLFM-*ti* is involved in the biosynthesis of efrapeptin G, containing Aib and l-Iva. In this case, tqaL is encoded in efrapeptin BGC, and tqaFM are located at a different contig, at least 854 kbp apart [40]. Aziridinase homologue *tqaL-ha* was identified in the atroviridin-producing *Hypocrea atroviridis* genome [41]. Substrate specificity of this protein studies, as well as the structure of atroviridin containing both Aib and l-Iva, shows that TqaL-*ha* also accepts both Val and Ile as substrates (Figure 8A). The ability of the TqaLFM-*ti* system to produce d-Iva from l-allo-Ile was demonstrated in in vitro supplementation experiments, but to the best of our knowledge, the TqaLFM system in the peptaibol-producing strain was not previously characterized. Therefore, we identified TqaLFM homologues, named TqaLFM-*th*, in *Trichoderma* sp. SK1-7 genome (Table S8). Three proteins were found to be encoded in *Trichoderma* sp. SK1-7 on the same contig in two regions, separated by ~100 orfs (138kb). TqaL-th exhibited modest similarity with TqaL-pa (62%) and was closely related to tqaL-ha (85%). Other TqaL-th homologues were identified using sequence similarity network (SSN) analysis (Figure S8); nodes corresponding to identified aziridinases are marked. Homologous proteins originating from the *Trichoderma* species were found to form a distinct cluster (Figure 7B), including TqaL-*ha*. Sequence alignment of TqaL-*ha* and TqaL-*th* reveals no substitutions in substrate-binding regions, and alignment of AlpaFold3-generated structures reveals similar protein structures; the differences are located in the N-terminal disordered region, predicted with low confidence (Figure 7C, Figure S9). Genome neighborhood diagrams, generated with ESI-GNT, reveal that this organization of the TqaLFM system is conservative for *Trichoderma*. In all available sequences, BZIP domain-containing protein, putatively involved in regulation, and thioesterase domain-containing protein were encoded upstream to TqaL (Figure 7D). Genes encoding TqaL and TqaF proteins are separated with one gene encoding a poorly characterized protein showing similarity with the ATP synthase alpha chain precursor (74%). Strict conservation of this architecture might indicate involvement of these proteins in the regulation or biosynthesis of Aib and Iva in *Trichoderma*. PKS-like Orf6 and adenylation domain-containing Orf7 in *Trichoderma* sp. SK1-7 were not conserved among other *Trichoderma*. Genes showing similarity with *tqaM* are located separately in analyzed genomes, typically at a comparable distance (100 ORFs) from *tqaLF*. No strict conservation of the genomic context in this region was observed in TqaM GNT analysis.

A lack of obvious regulation in peptaibol production suggests that in vitro concentrations of crucial amino acids might determine the resulting composition of the peptaibol fraction. To test this hypothesis, we performed supplementation of *Trichoderma* sp. SK1-7 with key amino acids Aib and Iva. Prolonged cultivation was applied to maximize the production of all types of peptides. MALDI analysis of feeding experiments with *Trichoderma* sp. SK1-7 with Aib, dl-Iva and Aib/dl-Iva combination revealed that Iva-only supplementation not only resulted in Aib substitution with Iva, but also shifted the SF4/SF1 ratio in comparison with control and Aib + Iva supplementation (Figure 7E).

### 2.4. Trichorozins Exhibit Moderate Antibacterial and Cytotoxic Properties, Induce Membrane Permeabilization and Potentiate Membrane-Active Compounds

The isolated compounds **1** (trichorozin IV) and its homologue **2** (trichorozin V) antimicrobial activity spectra were assessed on 15 strains of Gram-positive and Gram-negative bacteria, among which there are strains of *Escherichia coli* with a sensitive outer membrane—*E. coli* lptD^mut^ and *E. coli* Δ*tolC* KanR. The results indicate that strains such as *Micrococcus luteus* ATCC 4698, *Arthrobacter* ATCC 21022 and *Macrococcus caseolyticus* 107 are most sensitive to the compounds (Figure 8A, Table S9). The least sensitive are *Bacillus cereus X1* and *Bacillus subtilis* 168. Compound **2** exhibits a higher inhibitory effect in comparison with **1**. The toxic effect of SK1-7P1 and SK1-7P2 was assessed on the HEK 293T cell line (human embryonic kidney cells) (Figure S10). Cell viability analysis demonstrates moderate cytotoxic activity for **1** (IC_50_ = 3 ±1 μg/mL) and **2** (IC_50_ = 5 ± 1.2 μg/mL).

To determine the extent to which membrane permeabilization of Gram-positive bacteria contributed to the antimicrobial activity of the compounds, we examined the ability to increase inner membrane permeability. The permeability of the intracellular membrane of *M. luteus* was investigated using the fluorescent probe SYTOX red dead stain to penetrate cells. SYTOX cannot cross the intact cell membrane but can only cross the damaged cell membrane and stain for nucleic acid, emitting red fluorescence (Figure 8B). The median fluorescence intensity (RL1 channel) is presented as a measure of membrane damage, with higher values indicating greater permeabilization. Untreated cells exhibited minimal fluorescence, indicating intact membranes with limited dye penetration. The lantibiotic nisin, used as a positive control, displayed the highest membrane-disrupting capability. These results suggest that both compounds **1** and **2** exert their antimicrobial effects at least partially through the disruption of bacterial membrane integrity, with **2** demonstrating membrane-permeabilizing activity more comparable to the pore-forming lantibiotic nisin.

Assessment of the synergistic effect revealed various effects of **1** and **2** on antimicrobial activity of clinically applied antibiotics. The addition of peptaibols in cultivation medium led to pronounced activity enhancement for colistin and slight potentiation for nisin, meropenem and ciprofloxacin (Figure 8C). Calculated fractional inhibitory concentration (FIC) value for colistin in the presence of **1** (FIC**_1_**_-C_) is 0.25, which is responsible for **1** promoting the action of colistin, while the addition of colistin has no effect on the antibacterial activity of **1** (FIC_C-**1**_ = 1). Given the co-expression of short peptaibols with longer counterparts, their ecological role might involve modulation of the biological activity of other compounds, including partial membrane permeabilization.

## 3. Discussion

Long and even-numbered peptaibol predominate in naturally occurring mixtures, presumably due to more pronounced membrane activity achieved by transmembrane spanning. An 11AA peptaibols length corresponds approximately to a half-bilayer spanning helix [43]. Nonetheless, these compounds have ion channel formation ability, highly dependent on even subtle changes in amino acid composition [19].

Fungi typically produce a wide variety of peptaibols in complex mixtures, usually combining short- and long-chain compounds. Recent metabolomic and molecular networking studies show that short peptaibols are accompanied by long counterparts; for example, lipopetaibols lipostrigaibols A-E and strigaibols A-H from *Trichoderma strigosum* [44] and endophytins from *Trichoderma endophyticum* [29]. In this work, genome mining reveals that SF4-type peptaibols BGCs are encoded by the strains, metabolically characterized only as long peptaibols producers, suggesting these peptides may act in combination or modulate the overall biological effect of mixtures.

The module skipping mechanism is not yet well understood, but it is very important for NRPS systems engineering [45]. First reported for nyxochromides biosynthesis, it was further widely applied for diversification of this antibiotic family by synthetic biology approaches [46]. For example, A-domain deletion in plipastatin biosynthesis led to a variety of products with module skipping [47]. Wide genomic characterization of SF4-type peptaibols synthase may provide useful insights into the molecular basis of module skipping. Recently isolated from *Trichoderma* sp. RK10-F026 11-residue peptaibols, zealpeptaibolins share some sequence similarities with 14-residue peptaibols and therefore are speculated to be formed via module 1, 2 and 6 skipping in tex2-like NRPS [48]. 11AA peptaibols trichofumins A, B detection in *Trichoderma* sp. HKI 0276 was accompanied by isolation of 13AA peptaibols trichofumins C and D, sharing some resemblance with SF4 sequences but containing a Gln-Gln motif, therefore not accessible by module skipping in *tex2*-like genes [22]. These peptides may originate from module skipping in larger NRPS, but unfortunately no genetic information is available for these strains. Further combined genomic and metabolomics profiling of peptaibol-producing species can unlock the hidden diversity of enzymatic machinery capable of wide chemical diversification of non-ribosomal peptides. Moreover, genome mining revealed a conservative architecture of Aib/Iva biosynthetic machinery in *Trichoderma* that might be crucial for further advances in TqaLFM investigation and application.

Predominantly, studies on structure–activity relationships in peptaibol families provide information on the biological evaluation of the isolated substances, whereas some reports indicate that the prominent membrane activity can be achieved only by a combination of components [49]. Recently isolated peptaibols were found to act synergistically with the clinical membrane-permeabilizing drug colistin [3]. Peptaibols previously were shown to exhibit significantly higher antileishmanial activity in combination with miltefosine [50]. SF4 peptaibols 1 and 2 in this work also demonstrated a synergistic effect with clinical drugs, indicating the high potential of peptaibols as membrane-active adjuvants resistant to proteolysis.

Advancements in the tools and techniques used for peptabiotics screening and purification, along with their wide range of biological activity, spur intensive investigations of this chemical space [51]. Our findings suggest that genome-guided approaches hold promise for further structural diversification of peptaibol-related compounds and deeper understanding of their ecological role and plausible application as adjuvants. Moreover, the molecular basis of unique enzymatic features of peptaibol synthases, such as obscure regulation, substrate promiscuity and module skipping, can be further applied for the creation of new peptide-based antimicrobials via a synthetic biology approach by targeted NRPS engineering.

## 4. Materials and Methods

### 4.1. Isolation and Fermentation of the Producing Strain Trichoderma sp. SK1-7

The cut, untreated spruce logs had been lying outdoors for 1 month in the village of Khombus-Batyrevo, Chuvash Republic, Russian Federation (55.293515 N, 47.088331 E). Bark samples were stored in sterile containers. Preliminary bark samples were washed with sterile water and suspended for inoculation. The inoculum was spread on 9 × 9 cm Petri dishes with solid nutrient medium brain heart infusion (with nystatin 50 μg/mL), potato dextrose (with tobramycin 25 μg/mL), nutrient agar (with nystatin 50 μg/mL) and nutrient agar (with nystatin 50 μg/mL and nalidixic acid 30 μg/mL). The dishes were incubated at 28 °C under aerobic conditions for 10 days to ensure the isolation of the less-abundant bacteria, since they require a longer period to form colonies. As the colonies grew, they were transferred and purified on potato-dextrose agar (fungi) or nutrient agar (bacteria). The strains were stored in glycerol (20%, *v*/*v*) at −87 °C. In total, 46 microbial isolates were isolated. Antimicrobial activity was screened using the agar diffusion method against a wide range of test microorganisms: a fungus (*Aspergillus niger* INA 00760), a yeast (*Candida albicans* CBS 8836), Gram-positive bacteria (*Bacillus subtilis* ATCC 6633, *Enterococcus faecalis* ATCC 29212) and Gram-negative bacteria (*Escherichia coli* ATCC 25922).

The strain *Trichoderma hombus* SK1-7 was transferred from the surface of the agar potato-dextrose medium into a 750 mL Erlenmeyer flask with 100 mL of potato-dextrose broth. Cultivation was carried out in a thermostat at 28 °C without stirring for 14 days.

Biomass was separated from the culture broth by vacuum filtration. A total of clarified supernatant was subjected to fractionation using an LPS-500-H (copolymer divinylbenzene-hydrophilic monomer, pore size 50–1000 Å, 70 µm, Technosorbent, RF) column. After initial sorption on the column, it was eluted stepwise with increasing concentrations of acetonitrile in water: 0 → 10 → 20 → 30 → 40 → 50 → 75 → 100% MeCN. The fractions with antimicrobial activity were analyzed by HPLC. The biomass was extracted with ethanol (10/1). An ultrasonic bath was used for better mixing of the solvent with the biomass.

### 4.2. Bioactivity-Guided Fractionation, Isolation of Peptaibols

Crude ethanolic mycelium extract and culture broth fractions were centrifuged in 2 mL snap-seal microtubes with Eppendorf 5424 centrifuge for 5 min at 24,000× *g.* Then, 100 μL of supernatant was injected in analytical-scale preparative HPLC and collected in 96 deep-well plates (Eppendorf Deepwell Plate 96/2000 µL). Each collected fraction was evaporated in a centrifuge dryer (Labconco CentriVap Centrifugal Concentrators with Cold Trap) and was dissolved in 100 μL of 10%DMSO in water, and the resulting solution was then subjected to a serial dilution activity determination assay against *Bacillus subtilis* ATCC 6633 as a test strain. Active fractions were then subjected to HRESIMS analysis; resulting HRESIMS-MS spectra were dereplicated using mzMine package ver. 4.3.0 and publicly available databases.

For structure elucidation and biological experiments, medium-scale preparative isolation was performed. Mycelium extract was dried under reduced pressure, extracted with MeOH and then separated with semi-preparative chromatography (Thermo Ultimate 3000 with AS-AP autosampler, Rheodyne manual injector, DAD, Binary Pump and TCC). The condition was A—Water(mQ), B—MeCN. Gradient elution: 50–95% B in 15 min and isocratic elution of 95% B in 5 min with 5 mL/min flow rate. Column—Waters Xbridge Peptide BEH 300 C18 5 μm (10 × 250 mm). Injection volume was 500 μL. Purity was controlled by analytical HPLC (Thermo Ultimate 3000 with TBFC autosampler, DAD, Binary Pump and TCC) with gradient elution: 5–95% B in 10 min and isocratic elution of 95% B in 4 min. Column—Waters Xbridge Peptide BEH 300 C18 5 μm (4.6 × 250 mm). Injection volume was 10 μL.

### 4.3. HRMS Analysis

LC-MS analysis was carried out on an Ultimate 3000 nanoRSLC HPLC system connected to a QExactive Plus mass spectrometer (Thermo Fisher Scientific, Waltham, MA, USA). Samples were separated on a Gemini C18 3 μm NX LC column 100 × 2.1 mm (Phenomenex, Torrance, CA, USA) at 200 uL/min flow rate. Separation was performed by a linear gradient of 90% acetonitrile in water, 10 mM ammonium formate, 0.1% formic acid (Buffer B) in 99.9% H_2_O, 10 mM ammonium formate, 0.1% formic acid (Buffer A). UV data was collected at 220 nm. MS1 and MS2 spectra were recorded at 30K and 15K resolution, respectively, with HCD fragmentation. Raw data was collected and processed on the Thermo Xcalibur Qual software ver. 4.3.73.11. The MS peaks were extracted at a mass tolerance of 5 ppm.

### 4.4. Structure Elucidation

The structures of trichorosin IV (**1**) and trichorosin V (**2**) were elucidated using the conventional heteronuclear NMR approach. The analyzed compounds were dissolved in 270 µL of DMSO-d6 (100%, Sigma-Aldrich, St. Louis, MO, USA) and placed into a 5 mm Shigemi NMR tube (SHIGEMI, Tokyo, Japan). NMR spectra were recorded at 30 °C using a Bruker Avance III 600 MHz NMR spectrometer (Bruker, Billerica, MA, USA) equipped with a TCI cryogenic probe. The spectra recorded included 1D ^1^H and ^13^C, as well as 2D experiments such as ^1^H,^13^C-HSQC; ^1^H,^13^C-HMBC; DQF-COSY; ^1^H,^1^H-TOCSY (mixing time = 200 ms); and ^1^H-^1^H NOESY (mixing times of 50 and 150 ms) for each sample.

The obtained NMR spectra were processed using Topspin software version 3.2 (Bruker, USA) and analyzed with the CARA software version 1.9.1 [52]. The assignment of the polypeptide chain was performed according to a standard scheme [53].

The absolute configurations of the amino acid constituents of compounds **1** and **2** were determined through acid hydrolysis followed by application of the advanced Marfey’s method. N-(5-Fluoro-2,4-dinitrophenyl)-l-leucinamide (l-FDLA) was prepared according to standard protocol [54]. For the analysis, 0.2 mg aliquots of 1 and 2 were separately dissolved in 0.2 mL of 6 N HCl and heated at 100 °C for 120 h in sealed vials. After hydrolysis, the samples were evaporated to dryness and redissolved in 50 μL of water. Each solution was then treated with 86 μL of a 1% acetone solution of l-FDLA (30 mM, providing a molar ratio of l-FDLA to amino acid of 1.4:1 along with 10 μL of 1 M NaHCO_3_. The reaction mixtures were incubated at 37 °C for 3 h with agitation, then neutralized with 10 μL of 1 N HCl. Following vacuum drying, the samples were dissolved in DMSO for HPLC analysis. To establish reference values, both l- and d-amino acid standards, as well as their racemic mixtures, were derivatized with l -FDLA under identical conditions. For glutamine analysis, l- and d-glutamic acid standards were used since glutamine is converted to glutamic acid during acid hydrolysis. All l- and d-amino acid standards (or their racemic mixtures) were obtained from Sigma-Aldrich, except for l-Leuol/dl-Leuol, which was synthesized from l-Leu/dl-Leu, respectively, using a standard reduction method [55]. As enantiopure isovaline standards were unavailable for direct comparison, peak assignments for l-Iva and d-Iva were made in accordance with previously documented l → d elution order of l-FDLA-derivatives [5,44,56].

Analysis was performed by analytical HPLC (Agilent 1100 (Agilent, Santa Clara, CA, USA) with a quaternary pump, well-plate autosampler and DAD) using a 45 min linear gradient from 10% to 50% acetonitrile (B) in 0.1 M NH_4_OAc/2% TFA in water (pH 3.0, A) at a flow rate of 1.2 mL/min. Column—Agilent Poroshell 120 EC-C18 (2.7 μm, 4.6 × 150 mm), injection volume—5 μL. Detection was performed at 340 nm [57].

The retention times for the l-FDLA derivatives of standard amino acids were determined as follows: l-Glu/ d-Glu (26.3/27.8 min), l-Pro/ d-Pro (28.0/31.3 min), Aib (32.7 min), l-Iva/d-Iva (34.0/37.2 min), l-Ile/d-Ile (34.6/43.5 min), l-Leu/d-Leu (35.2/43.9 min), l-Leuol/ d-Leuol (35.7/44.3 min).

Upon analysis, the hydrolysate from compound **1** displayed signals corresponding to l-Glu (26.2 min, derived from l-Gln), l-Pro (28.0 min), Aib (32.8 min), l-Ile (34.6 min), l-Leu (35.3 min) and l-Leuol (35.7 min), whereas the hydrolysate from **2** additionally contained a peak for d-Iva (37.2 min) alongside l-Glu (26.3 min), l-Pro (28.1 min), Aib (32.9 min), l-Ile (34.6 min), l-Leu (35.2 min) and l-Leuol (35.6 min). Peak assignments for the FDLA-derivatized hydrolyzate components were validated by LC-MS.

### 4.5. DNA Sequencing

The FastFS DNA Library Prep Set (MGI) was used for whole-genome library preparation. DNA nanoballs were prepared using the DNBSEQ-G400RS High-throughput Sequencing Kit (FCL PE150) reagents (MGI, Shenzhen, China). Sequencing was performed on the DNBSEQ-G400 (MGI) platform in 2 × 150 bp paired-end mode. For long-read sequencing, DNA libraries were prepared using the Ligation sequencing gDNA Native Barcoding Kit (SQK-NBD114, Oxford Nanopore Technologies, Oxford, UK). The libraries were loaded on the PromethION R10 flow cell and sequenced using the PromethION platform (Oxford Nanopore Technologies).

### 4.6. Bioinformatic Analysis

For the basecalling of Oxford Nanopore data, dorado v 0.7.2 (model dna_r10.4.1_e8.2_400bps_sup@v5.0.0) [58] was used. Nanopore reads were then assembled using Flye v 2.9.5 [59] followed by polishing with MGI reads via pilon v 1.24 [60]. Gene prediction was performed using AUGUSTUS (https://bioinf.uni-greifswald.de/webaugustus/, accessed on 30 April 2025) in the web version (with AUGUSTUS species parameters: *Aspergillus fumigatus*) [61]. Biosynthetic gene clusters were searched using antiSMASH 7 [62].

Phylogenetic analysis was carried out using the full fragment of an internal transcribed spacer (ITS1-5.8S-ITS2), *tef1* (Elongation factor 1-alpha) and *rpb2* (DNA-directed RNA polymerase subunit). These fragments were isolated from the assembly by in silico PCR using the ipcress tool from the exonerate software package [63]. The primers ITS5 (5′-GGAAGTAAAAGTCGTAAGTCGTAACAAGG-3′) and ITS4 (5′-TCCTCCTCCGCTTATTATTGATATATATGC-3′) [64] were used to isolate the complete ITS fragment. The pair of primers EF1-728F (5′-CATCGAGAAGTTCGAGAAGG-3′) [65] and TEF1LLErev (5′-AACTTGCAGGCAATGTGG-3′) were used for the *tef1* region isolation and the fRPB2–5 f (5′-GAYGAYMGWGATCAYTTYGG-3′) and fRPB2–7cr (5′-CCCATRGCTTGYTTRCCCAT-3′) [66] were used for isolation *rpb2*.

Additionally, for phylogenetic analysis, all sequences collected in the publication [67] were downloaded by their GenBank ID. The ITS *+ tef1 + rpb2* and tef1-rpb2 gene concatenates underwent independent phylogenetic analysis. Multiple alignment was performed using MAFFT v7.453 [68]. The phylogenetic tree was constructed with the maximum likelihood approach using FastTree v2.1.11 with default settings [69]. Phylogenetic trees were also constructed on a separate subsample of the clade of species related to the studied fungus. The phylogenetic trees were visualized using iTOL (https://itol.embl.de/, accessed on 21 March 2025).

Comparative genomics analyses were performed using OrthoVenn2 (http://121.37.229.61:9999/home, accessed on 28 March 2025) with default settings [70]. Annotation of phylogenetically closely related species was also performed using AUGUSTUS in the web version (with AUGUSTUS species parameters: *Aspergillus fumigatus*). Accession numbers of complete genome sequences, obtained through genome mining, are provided in Supplementary Materials (Table S6). Annotation was performed using AUGUSTUS (https://bioinf.uni-greifswald.de/webaugustus/, accessed on 30 April 2025) in the web version (with AUGUSTUS species parameters: *Aspergillus fumigatus*) [61]. Biosynthetic gene clusters were extracted and analyzed using antiSMASH 7 [62]. BGC comparison was preformed using CAGECAT (https://cagecat.bioinformatics.nl/, accessed on 30 April 2025) [30]. Sequence alignments were obtained with MEGA X ver. 10.0.5 [71]. Substrate specificities of adenylation domains were predicted using PARAS (https://paras.bioinformatics.nl/, accessed on 30 April 2025) [31]. Illustrations were created in Inkscape 1.1.1 (3bf5ae0d25, 2021-09-20). The sequence similarity network (SSN) of TqaL-th was generated via the EFI-EST (https://efi.igb.illinois.edu/efi-est/, accessed on 30 April 2025), and the SSN is visualized by Cytoscape 3.9.1 [72]. The genome neighbor of TqaFM-*th* homologues are analyzed using EFI-GNT (https://efi.igb.illinois.edu/efi-gnt/, accessed on 30 April 2025) [73,74].

### 4.7. Feature-Based Molecular Network (FBMN)

HPLC fractionation of mycelium extract was performed on Waters Xbridge Peptide BEH 300 C18 5 μm (10 × 250 mm); peptaibols with a retention time of 9–12 min were analyzed. Gradient elution: 50–95% B in 15 min and isocratic elution of 95% B in 5 min with 5 mL/min flow rate. HRESIMS-MS was processed using mzwizard tool in mzMine package ver. 4.3.0 using standard parameters for the HPLC-DAD-DDA combo; resulted files were uploaded to GNPS FBMN (https://gnps.ucsd.edu/ProteoSAFe/status.jsp?task=f2ee5924e25b426f8c71ba6580a7efde) and then, using Cytoscape 3.9.1 [72], FBMN was plotted. The resulted picture was cleaned up and reinforced with annotations using the Adobe Illustrator 2024 28.0 product.

### 4.8. Amino Acid Supplementation Experiment

The strain *T. hombus* SK1-7 was cultured on potato-dextrose broth in 250 mL Erlenmeyer flasks with 100 mL of medium each. Cultivation was carried out in a thermostat at 28 °C without stirring for 25 days. Amino acids were added to the nutrient medium before it was sterilized. α-Aminobutyric acid (Aib) and isovaline (iVal) were used as amino acid additives. There were a total of seven cultivation variations, with several concentrations of amino acids per liter: control without additives, 2 mM Aib, 5 mM Aib, 2 mM iVal, 5 mM iVal, 2 mM Aib + 2 mM iVal, 5 mM Aib + 5 mM iVal.

Biomass was extracted with ethanol, evaporated under reduced pressure, extracted with methanol, diluted with 50% water, passed through 50 mg of Technosorbent LPS-500H and eluted with 5 mL of acetonitrile and 5 mL of MeOH. Combined organic fractions were dried and redissolved in 10 mL of methanol to produce total peptaibol fraction. An amount of 0.5 mL of the solution was applied on 1 mL of 2,5-dihydrohybenzoic acid (Aldrich, 40 mg/mL in 30% acetonitrile and 0.5% TFA in water) and dried out. Mass spectra were recorded on the MALDI-TOF-TOF (spectrometer UltrafleXtreme (BrukerDaltonics (DE), Bremen, Germany)), with UV-laser(Nd) in positive mode with usage of reflectron; accuracy of monoisotopic molecular masses ([M+H]^+^) was in the range ±0.2 Da. Spectra were recorded in the range 600–6000 *m*/*z*, optimizing laser power for best possible resolution.

### 4.9. Bacterial Strains for Antimicrobial Activity Testing

A bacterial collection of clinical isolates including *Micrococcus luteus* ATCC 4698, *Bacillus cereus* X1, *Lactococcus lactis* 61, *Enterococcus faecium* 40, *Enterococcus faecalis* 125, *Macrococcus caseolyticus* 107, *Staphylococcus epidermidis* 39, *Staphylococcus haemolyticus* 515, *Pseudomonas aeruginosa* 51911 was kindly provided by Lytech Co., Ltd. (Moscow, Russia). *Staphylococcus aureus* constitutively producing GFP was kindly provided by Andrey Shkoporov from the Department of Microbiology and Virology, Russian National Research Medical University, Moscow. *E. coli* lptD^mut^ and *E. coli* ΔtolC KanR were kindly provided by Professor I.A. Osterman. Strains *Arthrobacter* sp. ATCC 21022, *Bacillus subtilis* 168 and *Escherichia coli* BL21(DE3) were purchased from Invitrogen Corp. (Thermo Fisher Scientific, Waltham, MA, USA).

### 4.10. Antibacterial Activity Assessment

Individual colonies of bacteria from the plate with 2YT agar medium (10 g/L yeast extract, 16 g/L tryptone, 5 g/L NaCl, 15 g/L agar) were transferred to 5 mL of 2YT nutrient medium (10 g/L yeast extract, 16 g/L tryptone, 5 g/L NaCl) and incubated at 37 °C overnight. The resulting overnight cultures were transferred to fresh nutrient medium in a ratio of 1:100 and incubated at 37 °C for 3 h. Next, the cultures were diluted so that the optical density of the final solution at 600 nm was ~0.001 a.u. This cell culture was used to study the spectrum of activity of antibiotics. Antibiotic MICs were determined after 16 h of incubation in a 96-well plate visually and at a wavelength of 600 nm using a Varioskan multimodal reader (Thermo Fisher Scientific, Waltham, MA, USA). An amount of 50 μg/mL was chosen as the initial concentration for antibiotics with subsequent two-fold serial dilutions.

Fractional inhibitory concentration of antimicrobial agent X in the presence of Y (FIC_Y-X_) was calculated as a ratio between the inhibitory concentration of X alone (MIC_X_) and the inhibitory concentration of X in combination with Y (C_X_) based on checkerboard assay.

### 4.11. Cell Viability Assay

To prepare the MTT solution, a dry sample of thiazolyl blue tetrazolium bromide was dissolved in phosphate-buffered saline, pH = 7.4 (DPBS) to a concentration of 5 mg/mL. The resulting solution was sterile-filtered through a filter with a pore diameter of 0.2 μm; the filtrate was collected in a sterile container protected from light. To prepare the solubilizing solution, 40% (vol.) dimethylformamide (DMF) was diluted in 2% (vol.) glacial acetic acid. To the resulting solution, 16% (*w*/*v*) sodium dodecyl sulfate (SDS) was added. Using 2% (vol.) glacial acetic acid, the pH of the solution was adjusted to 4.7.

Two-fold dilutions of the test compounds in DMSO were prepared as a negative control in the culture medium. A series of final concentrations of eight two-fold dilutions began with 6.4 μg/mL. Incubation was carried out in a humidified atmosphere at 37 °C with 8% CO_2_ for 72 h. Cytotoxicity was assessed on HEK293T cells.

Cell viability was determined by colorimetric assessment of the metabolic activity of cells (MTT). The analysis was carried out after incubation for 72 h. All measurements were performed in three biological replicates. An amount of 10 μL of MTT solution was added to the test samples per well to a final concentration of 0.45 mg/mL. Incubation was carried out for an hour at 37 °C. Then, 100 μL of solubilizing solution was added to each well to dissolve formazan crystals. The result was measured using a Varioskan multimodal reader (Thermo Scientific, Waltham, MA, USA) at an absorption of 570 nm. The obtained results were analyzed using GraphPad Prism ver. 10.4.0. Based on the MTT test results, a graph was constructed of the dependence of the average survival rate on the concentration of the test substance on a logarithmic scale. A well with cells that did not contain the test compounds was taken as 100% viability. Relative survival was calculated using the following formula: (absorption for a given well)/(absorption for a well without adding the drug) × 100%. The cytotoxic concentration for each compound (IC_50_) was determined from the obtained inverse sigmoid as the concentration value at which a cytotoxic effect of 50% of the cells in the monolayer was induced.

### 4.12. Membrane Premeability Assay

Bacteria were grown in flasks containing 2YT in a shaker-incubator at 28 °C overnight. The grown cultures were diluted to 10^6^ cell per mL in PBS buffer (10 mM, pH = 7.2, 0.22 μm pore size filtered) and treated with tested compounds for 30 min at room temperature and dyed by SYTOX (5 nM) (S34859, Invitrogen) for 15 min at room temperature. As a positive control, the lantibiotic nisin was used; as a negative control, dyed cells without treatment with compounds. Stained bacterial cells were assayed in a LongCyte flow cytometer (Beijing Challen Biotechnology Co., Ltd.) with a laser emitting at 638 nm. The threshold was based on side scatter (SSC) and forward scatter (FSC) and amounted to 500. Initial instrument settings were installed as follows: the photomultiplier tube (PMT) voltage values were FSC, SSC—400 V, RL1—500 V, compensation was not used. RL1 (red channel) was fitted with a 660/20 nm band pass filter. The doublets and singlets were separated based on FSC-H—FSC-A intensities. The red channel results were analyzed ratiometrically, and normalization was applied to the untreated samples.

## Data Availability

The data presented in this study are openly available in NCBI at BioProject number PRJNA1251403. The results of the Augustus and antiSMASH annotation are available on Zenodo via the link https://zenodo.org/uploads/15277528. The NMR data for this study is available from the corresponding author at alferovava@gmail.com or sterekhoff@gmail.com upon request.

## Supplementary Materials

The following supporting information can be downloaded at https://www.mdpi.com/article/10.3390/ijms26125599/s1. References [75,76,77,78,79,80,81,82,83,84,85,86,87,88,89,90,91] are cited in the Supplementary Materials.
