# Supplementary material for: Genome-Guided Metabolomic Profiling of Peptaibol-Producing Trichoderma
Source: Int J Mol Sci. 2025 Jun 11;26(12):5599. doi: 10.3390/ijms26125599 (PMC12192607; doi:10.3390/ijms26125599)
Supplement: Supplementary file 1 [file ijms-26-05599-s001.zip › ijms-3647584-SM.pdf]

Genome-guided metabolomic profiling of peptaibol-producing *Trichoderma*

Arseniy A Sinichich[1,2,†], Danil V. Krivonos[3,4,†], Anna A. Baranova[1], Mikhail Y. Zhitlov[1,2], Olga A. Belozerova[1], Vladislav A. Lushpa[1,3], Andrey V. Vvedensky[4], Marina V. Serebryakova[5], Anastasia I. Kalganova[1], Arsen M. Kudzhaev[1], Yuri A. Prokopenko[1], Sofiia S. Sinelnikova[1], Ekaterina A. Trusova[1], Sergey I. Kovalchuk[1], Elena N. Ilina[4], Stanislav S. Terekhov[1,*], Vera A. Alferova[1,5,*]

[1] Shemyakin-Ovchinnikov Institute of Bioorganic Chemistry, Miklukho-Maklaya 16/10, Moscow 117997, Russia; alferovava@gmail.com (V.A.A.); anjabaranowa@list.ru (A.A.B.); o.belozyorova@gmail.com (O.A.B.); kudzhaev_arsen@mail.ru (A.M.K.); .); tetrahydrofuran@mail.ru (Y.A.P.); xerx222@gmail.com (S.I.K.); sterekhoff@gmail.com (S.S.T.); l; katatrusova532@gmail.com (E.A.T.); sinelnikowa.sofia@yandex.ru (S.S.S.)

[2] Department of Chemistry, Lomonosov Moscow State University, Moscow 119992, Russia; droplbox38@gmail.com (M.Y.Z.); asinichich@yandex.ru (A.A.S.);

[3] Moscow Center for Advanced Studies, Kulakova str. 20, Moscow 123592, Russia; lushpa1696@gmail.com (V.A.L.); danil01060106@gmail.com (D.V.K)

[4] Research Institute for Systems Biology and Medicine (RISBM), 18, Nauchniy Proezd, 117246 Moscow, Russia; vvedenskiia@sysbiomed.ru (A.V.V.); ilinaen@gmail.com (E.N.I.)

[5] Belozersky Institute of Physico-Chemical Biology, Lomonosov Moscow State University, Moscow 119991, Russia; mserebr@mail.ru (M.V.S.)

* Correspondence: alferovava@gmail.com (V.A.A.); sterekhoff@gmail.com (S.S.T.)

† These authors contributed equally to this work.

Supplementary materials

Contents

Figure S1. Trichorozin IV (**1**) MS data ... 4

Figure S2. Trichorozin V (**2**) MS data ... 5

Table S1. Assignment of **1** (trichorosin IV) signals. ... 6

Table S2. Assignment of **2** (trichorosin V) signals. ... 10

Supplement S1. 2D-NMR data ... 14

Figure S3. Phylogenetic analysis of *Trichoderma* sp. SK1-7. ... 20

Table S3. Sequences used in the phylogenetic analysis. ... 21

Figure S4. Phylogenetic tree constructed by maximum likelihood mode based on *tef1+rpb2*concatenate ... 37

Figure S5. MS data for major 18-residue peptaibol **3** ... 38

Figure S6. MS data for major 18-residue peptaibol **4** ... 39

Table S4. Annotation of *tho1* BGC ... 42

Table S5. Annotation of *Tho2* BGC ... 43

Table S6. Sequences used for comparative analysis of *tho1* and *tho2* BGCs ... 44

Figure S7. Comparison of long peptaibol-synthase contacting clusters in related *Trichoderma* ... 46

Table S7. Substrate specificity analysis of adenylation domains ... 47

Table S8. Aib-biosynthesis associated genes analysis ... 49

Figure S8. Sequence similarity network (SSN) analysis of *tqaL-th* homologues ... 50

Figure S9. AlphaFold prediction for TqaL-th structure ... 51

Table S9. Antimicrobial activity of **1** and **2** 52

Figure S10. Evaluation of cytotoxicity for **1** and **2**. 53

Figure S1. Trichorozin IV (**1**) MS data

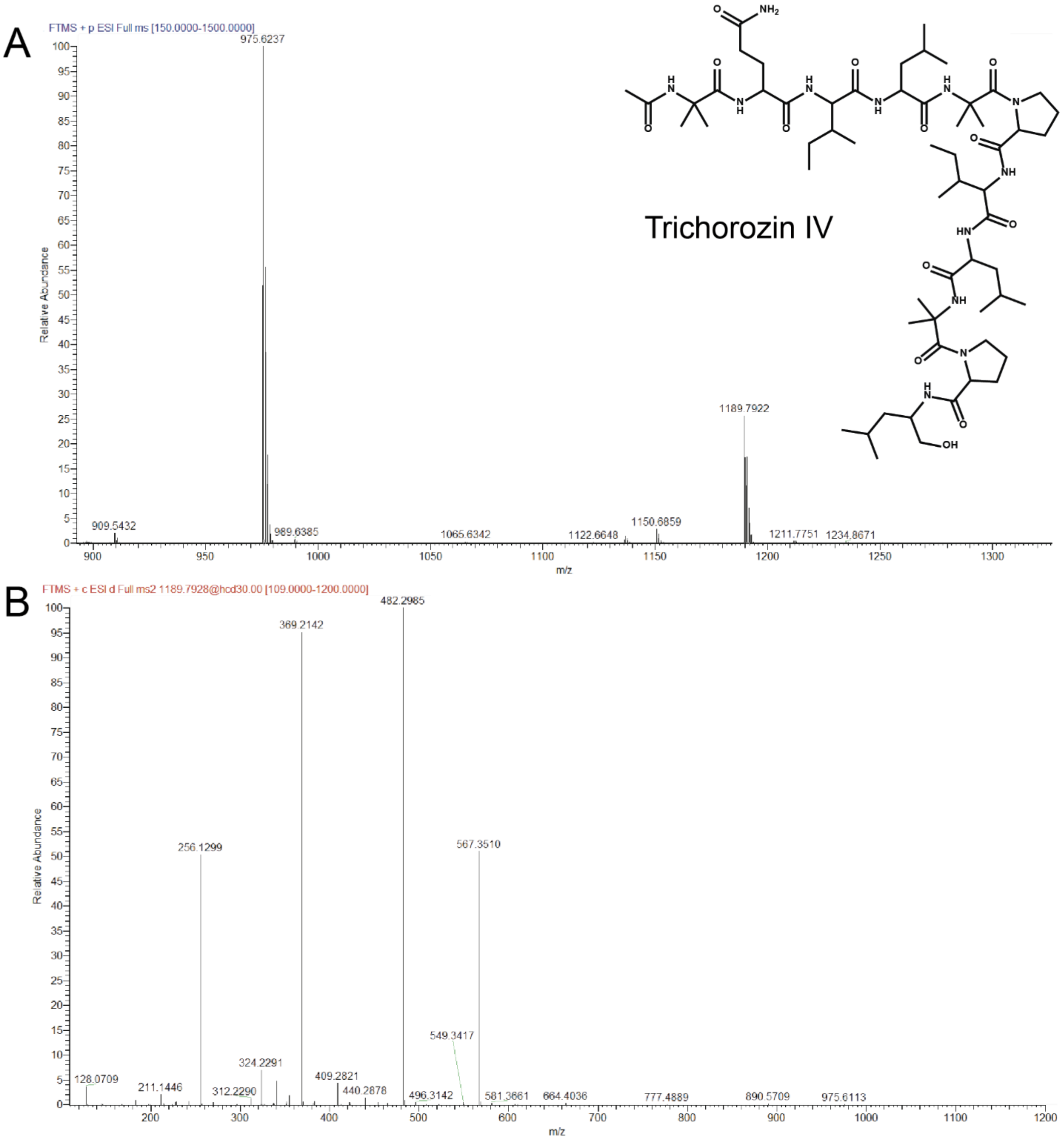

Figure S1. (A) Positive ion mode MS^1 spectrum of trichorozin IV (**1**); (B) HCD mass spectra of for parent ion $[M+H]^+$ at *m/z* 1189.7922.

Figure S2. Trichorozin V (**2**) MS data

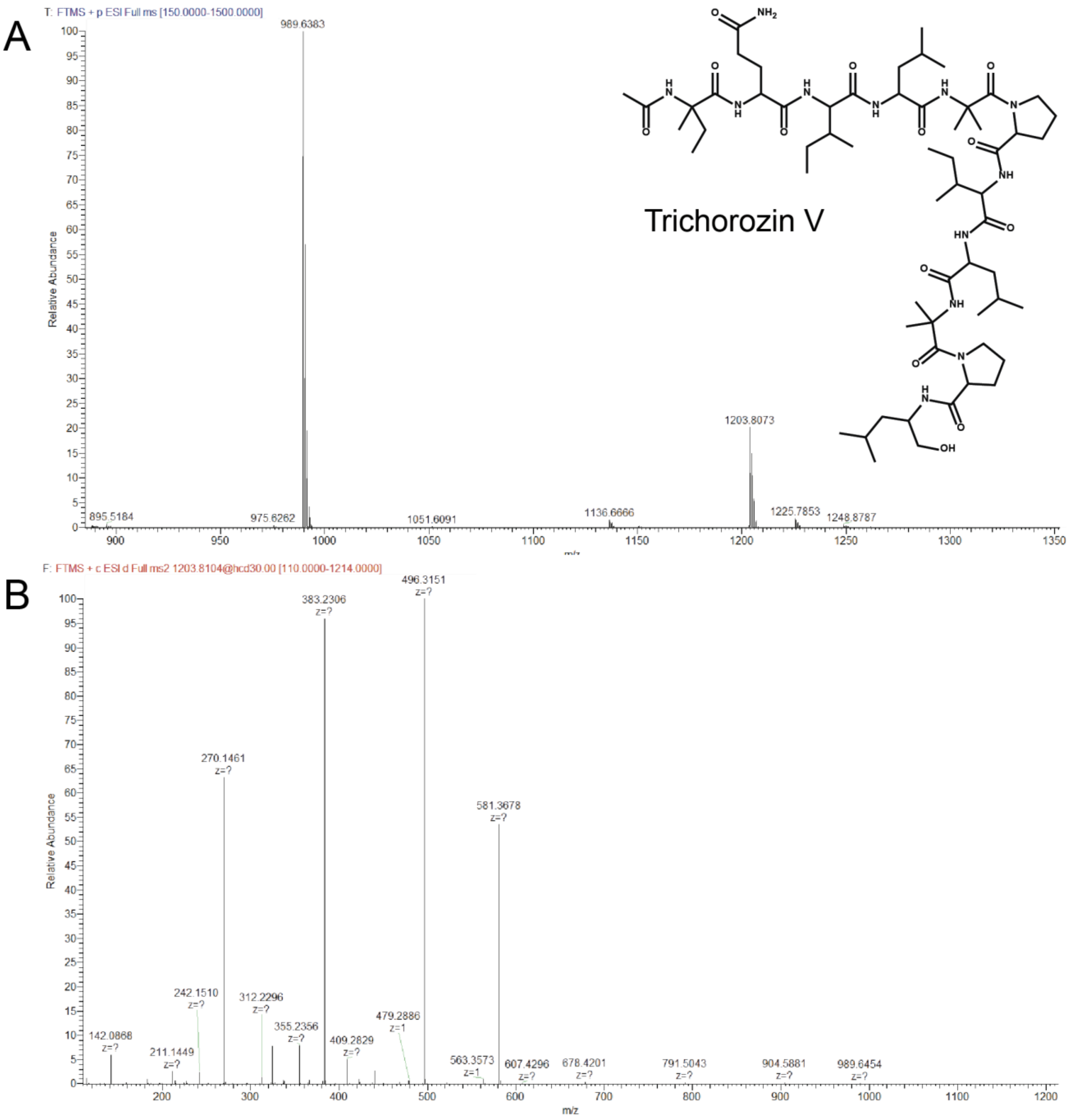

Figure S2. (A) Positive ion mode MS^1 spectrum of trichorozin V (**2**); (B) HCD mass spectra of for parent ion $[M+H]^+$ at *m/z* 1203.8073.

Table S1. Assignment of **1** (trichorosin IV) signals.

			δ, ppm	
№ aa	amino acid residue	chemical group	$^{13}C/^{15}N$	^{1}H
1	Aib	NH	-	-
		Cα	57.88	
		Cβ2/Cβ3	1.32/1.34	23.99/27.09
		CO	-	
2	Gln	NH	112.256	8.765
		Cα	56.167	3.950
		Cβ	26.123	1.935
		Cγ	31.902	2.181/2.258
		CO		
3	Ile	NH	115.605	7.617
		Cα	59.887	3.842
		Cβ	35.369	1.872
		Cγ1	25.928	1.238
		Cγ2	23.339	1.500
		Cδ1	15.823	0.868
		CO	-	
4	Leu	NH	113.253	7.457

		Cα	51.939	4.208
		Cβ	39.960	1.627
		Cγ	39.785	1.467
		Cδ1	23.268	0.840
		Cδ2	20.688	0.776
		CO	-	
5	Aib	NH	131.647	7.792
		Cα	-	
		Cβ2/Cβ3	25.831	1.369
		CO	-	
6	Pro	N	-	
		Cα	63.691	4.217
		Cβ	29.112	2.233/1.643
		Cγ	26.082	1.874
		Cδ	48.727	3.659/3.514
7	Ile	NH	110.455	7.299
		Cα	59.508	3.838
		Cβ	35.538	1.940
		Cγ1	29.349	1.239
		Cγ2	23.393	1.525

		Cδ1	11.363	0.865
		CO	-	
8	Leu	NH	112.871	7.184
		Cα	51.855	4.245
		Cβ	39.960	1.528
		Cγ	24.514	1.654
		Cδ1	23.469	0.812
		Cδ2	7.830	0.764
		CO	-	
9	Aib	NH	130.180	7.584
		Cα	-	
		Cβ2/Cβ3	24.122/26.063	1.367/1.334
		CO	-	
10	Pro	N	-	
		Cα	62.508	4.255
		Cβ	29.038	2.074/1.636
		Cγ	25.842	1.739
		Cδ	48.389	3.595/3.262
11	Leu	NH	115.351	7.065
		Cα	49.234	3.722

		Cβ	39.890	1.378
		Cγ1	24.799	1.592
		Cδ11	24.158	0.884
		Cδ12	22.028	0.803
		CO	-	
		Cγ2	64.452	3.321/3.130

Table S2. Assignment of **2** (trichorosin V) signals.

			δ, ppm	
№ aa	amino acid residue	chemical group	$^{13}C/^{15}N$	^{1}H
1	Iva	NH	-	
		Cα	58.88	
		Cβ1	27.22	1.690/1.976
		Cβ2	7.99	0.760
		Cγ1	23.32	1.280
		CO		
2	Gln	NH	113.150	8.778
		Cα	56.167	3.965
		Cβ	26.123	1.945
		CO	-	
3	Ile	NH	115.605	7.647
		Cα	59.887	3.845
		Cβ	35.369	1.866
		Cγ1	25.928	1.232
		Cγ2	23.339	1.500
		Cδ1	15.823	0.874
		CO	-	

4	Leu	NH	113.253	7.448
		Cα	51.939	4.214
		Cβ	39.960	1.612
		Cγ	39.785	1.467
		Cδ1	23.268	0.840
		Cδ2	20.688	0.776
		CO	-	
5	Aib	NH	131.647	7.785
		Cα	-	
		Cβ2/Cβ3	25.759	1.381
		CO	-	
6	Pro	N	-	
		Cα	63.691	4.217
		Cβ	29.112	2.233/1.643
		Cγ	26.082	1.874
		Cδ	48.727	3.666/3.514
7	Ile	NH	110.455	7.314
		Cα	59.508	3.850
		Cβ	35.538	1.940
		Cγ1	29.349	1.239

		Cγ2	23.393	1.513
		Cδ1	11.363	0.865
		CO	-	
8	Leu	NH	112.871	7.194
		Cα	51.855	4.245
		Cβ	39.960	1.528
		Cγ	24.514	1.654
		Cδ1	23.469	0.812
		Cδ2	7.830	0.764
		CO	-	
9	Aib	NH	130.106	7.602
		Cα	-	
		Cβ2/Cβ3	24.210/26.047	1.375/1.342
		CO	-	
10	Pro	N	-	
		Cα	62.508	4.255
		Cβ	29.038	2.074/1.636
		Cγ	25.842	1.739
		Cδ	48.389	3.595/3.262
11	Leu	NH	115.351	7.078

		Cα	49.234	3.729
		Cβ	39.890	1.378
		Cγ1	24.714	1.592
		Cδ11	24.157	0.892
		Cδ12	22.074	0.808
		CO	-	
		Cγ2	64.452	3.327/3.123

Supplement S1. 2D-NMR data

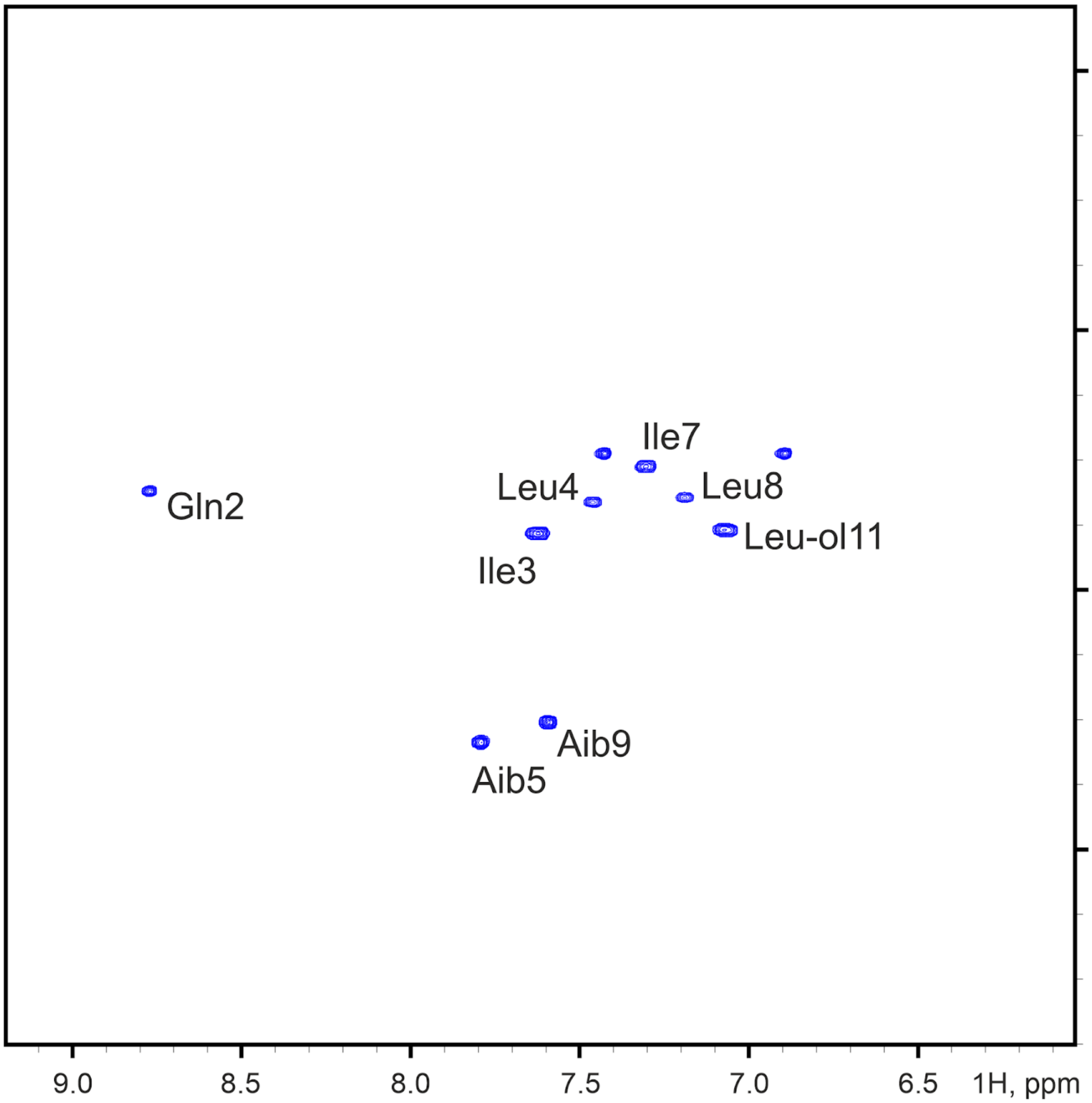

$^{1}H,^{15}N$- HSQC spectra of trichorozin IV. The assignment of NH signals to the protein backbone is shown.

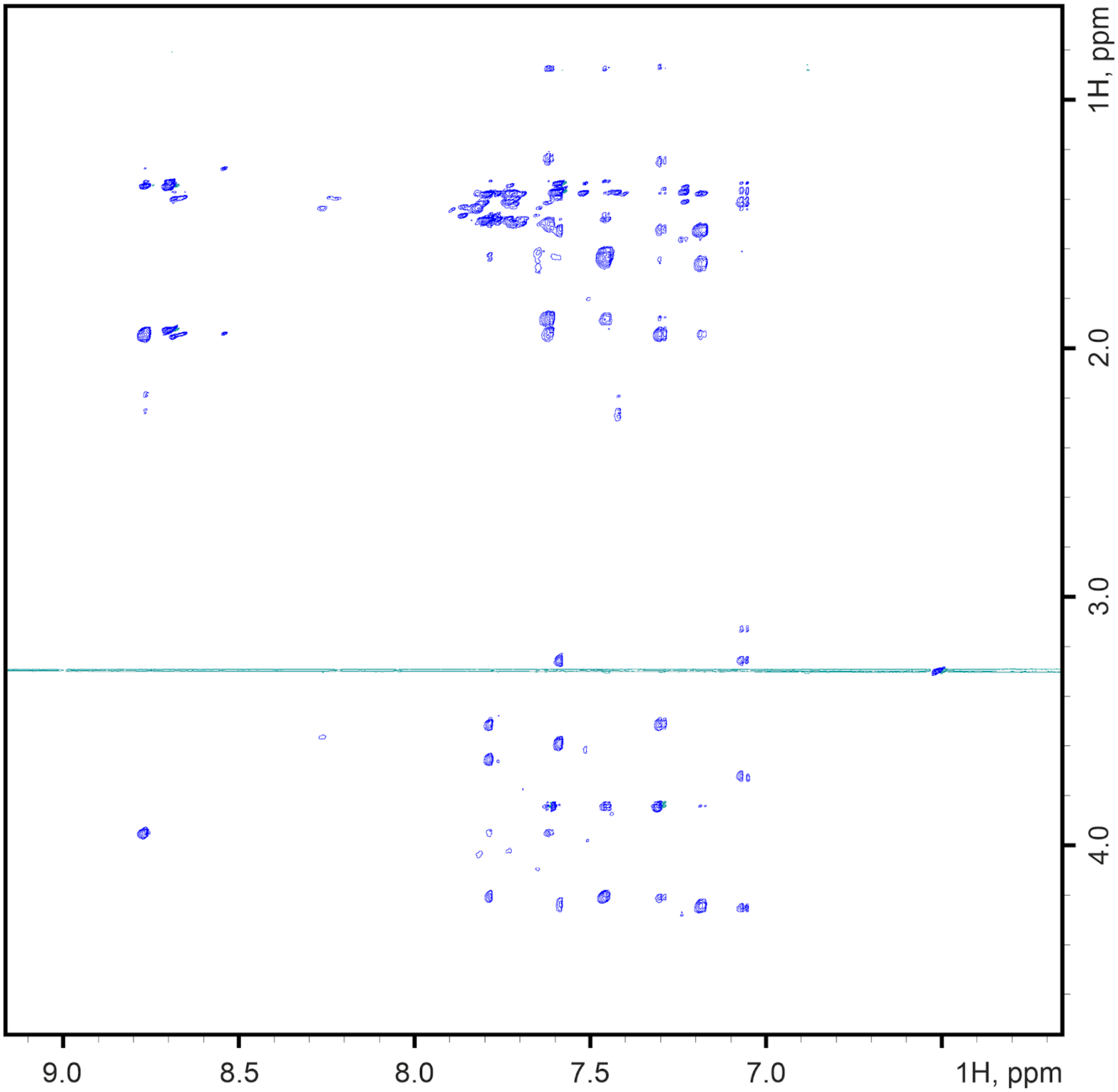

Fragment of $^{1}H,^{1}H$-NOESY spectra of trichorozin IV.

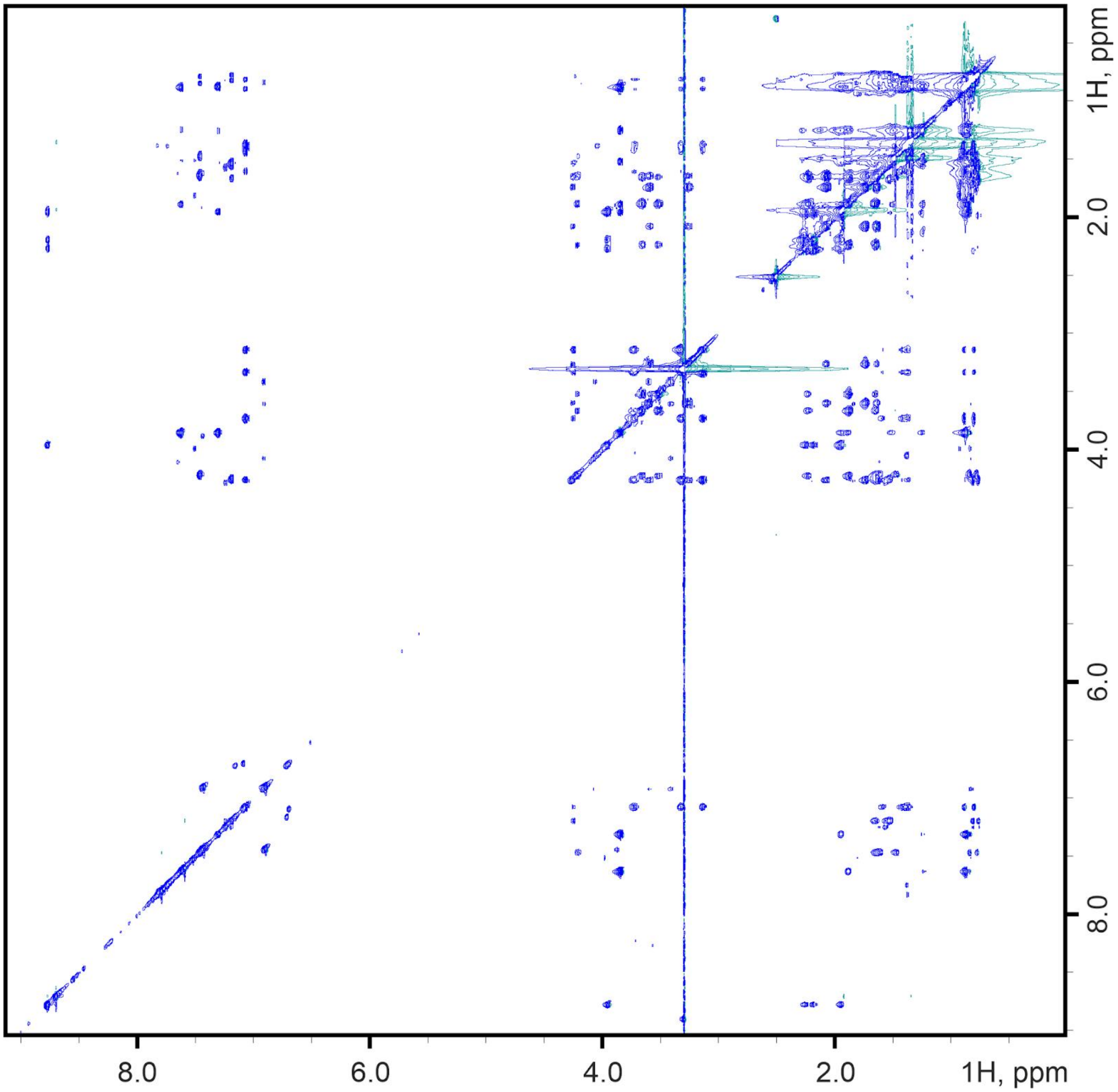

$^{1}H,^{1}H$-TOCSY spectra of trichorozin IV.

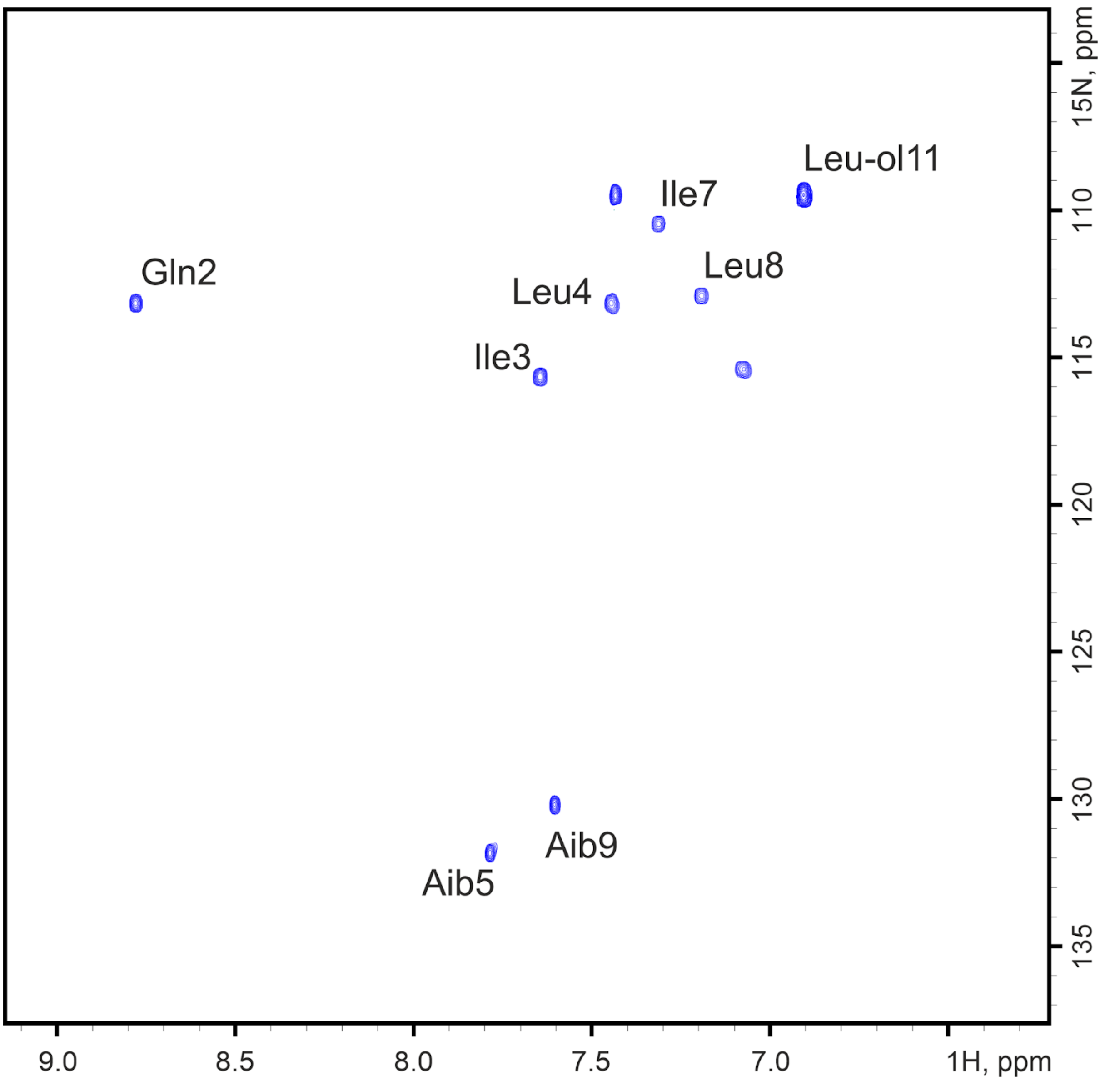

$^{1}H,^{15}N$- HSQC spectra of trichorozin V. The assignment of NH signals to the protein backbone is shown.

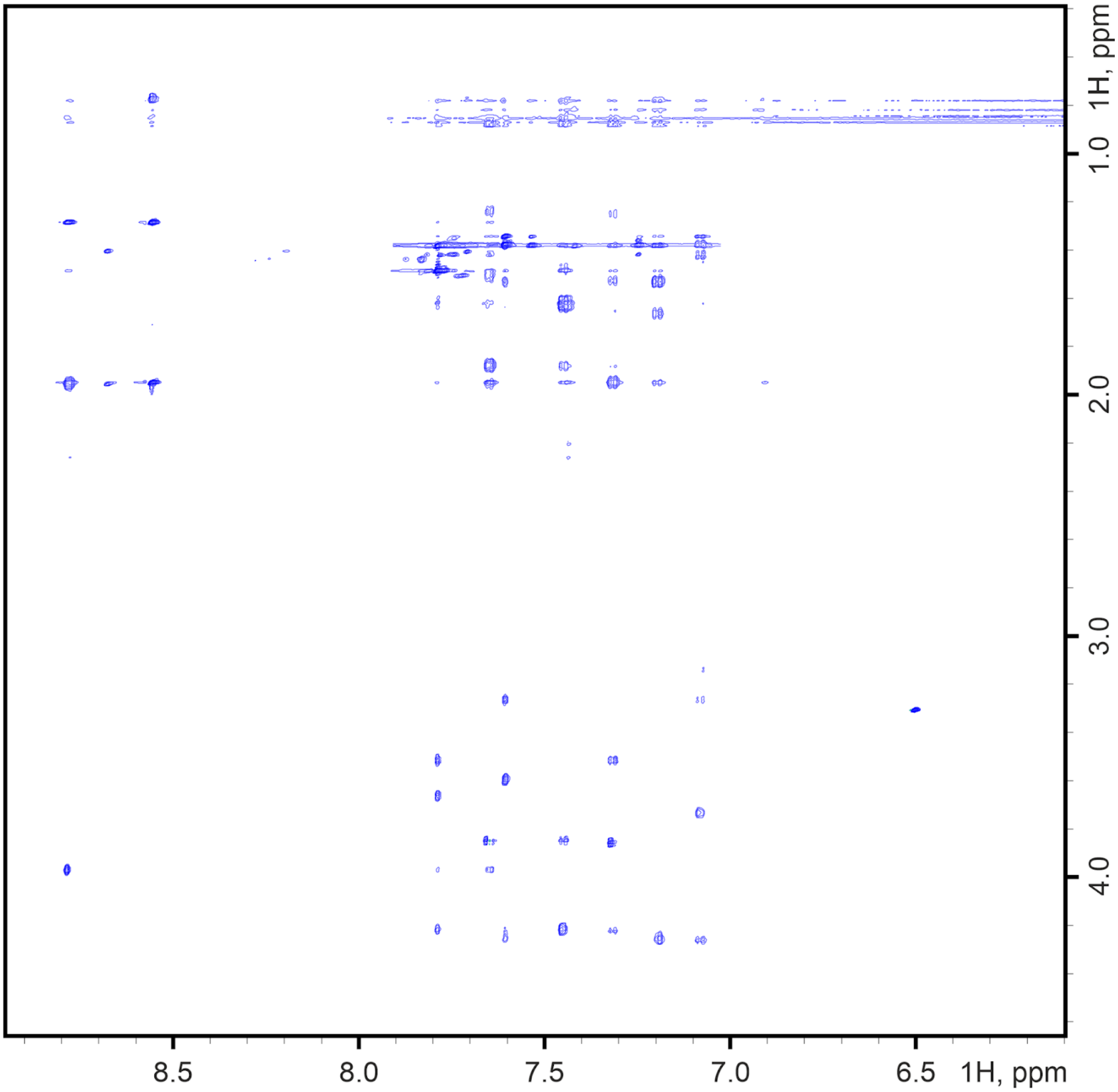

Fragment of $^{1}H,^{1}H$-NOESY spectra of trichorozin V.

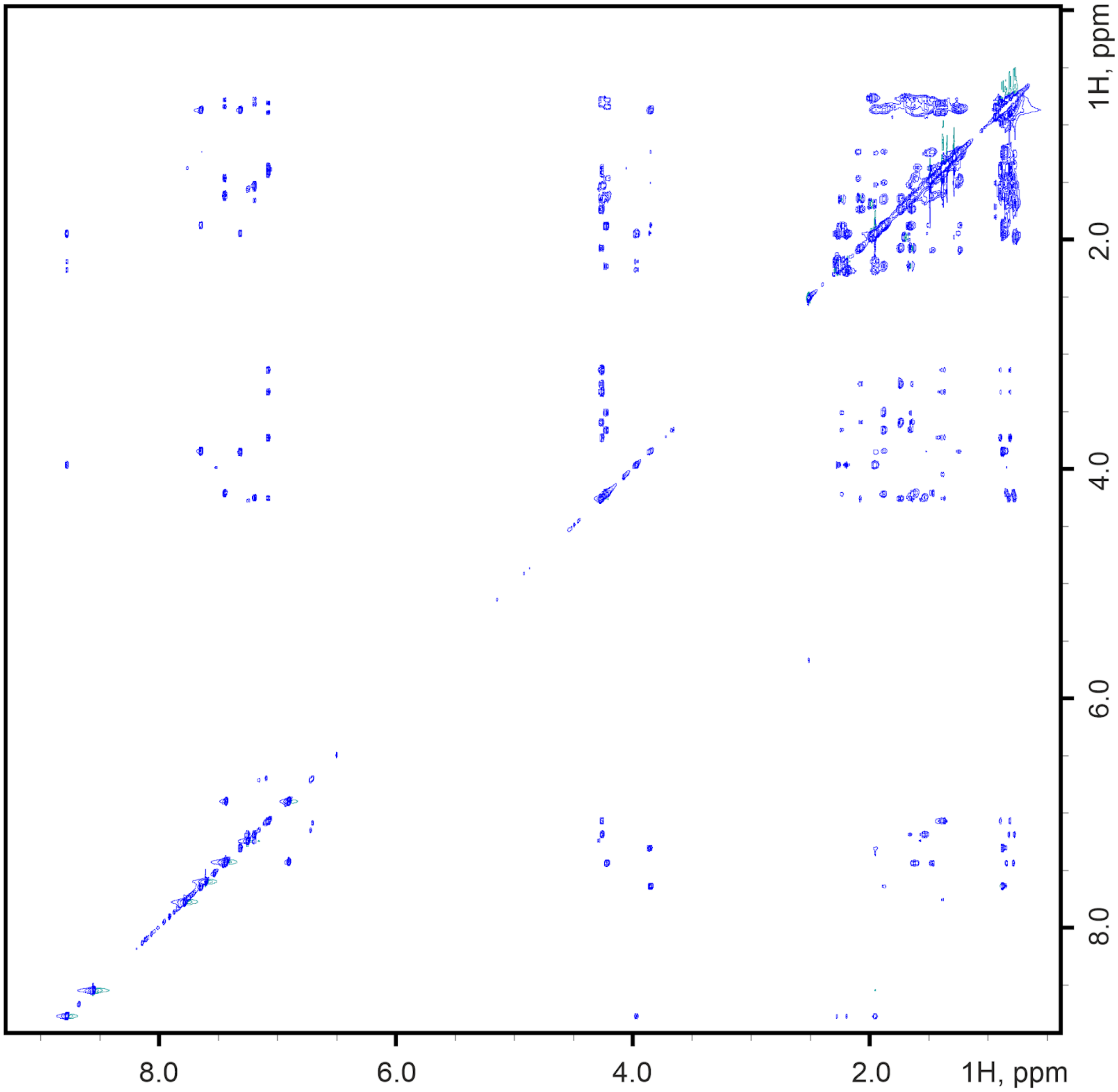

^{1}H,^{1}H-TOCSY spectra of trichorozin V.

Figure S3. Phylogenetic analysis of *Trichoderma* sp. SK1-7.

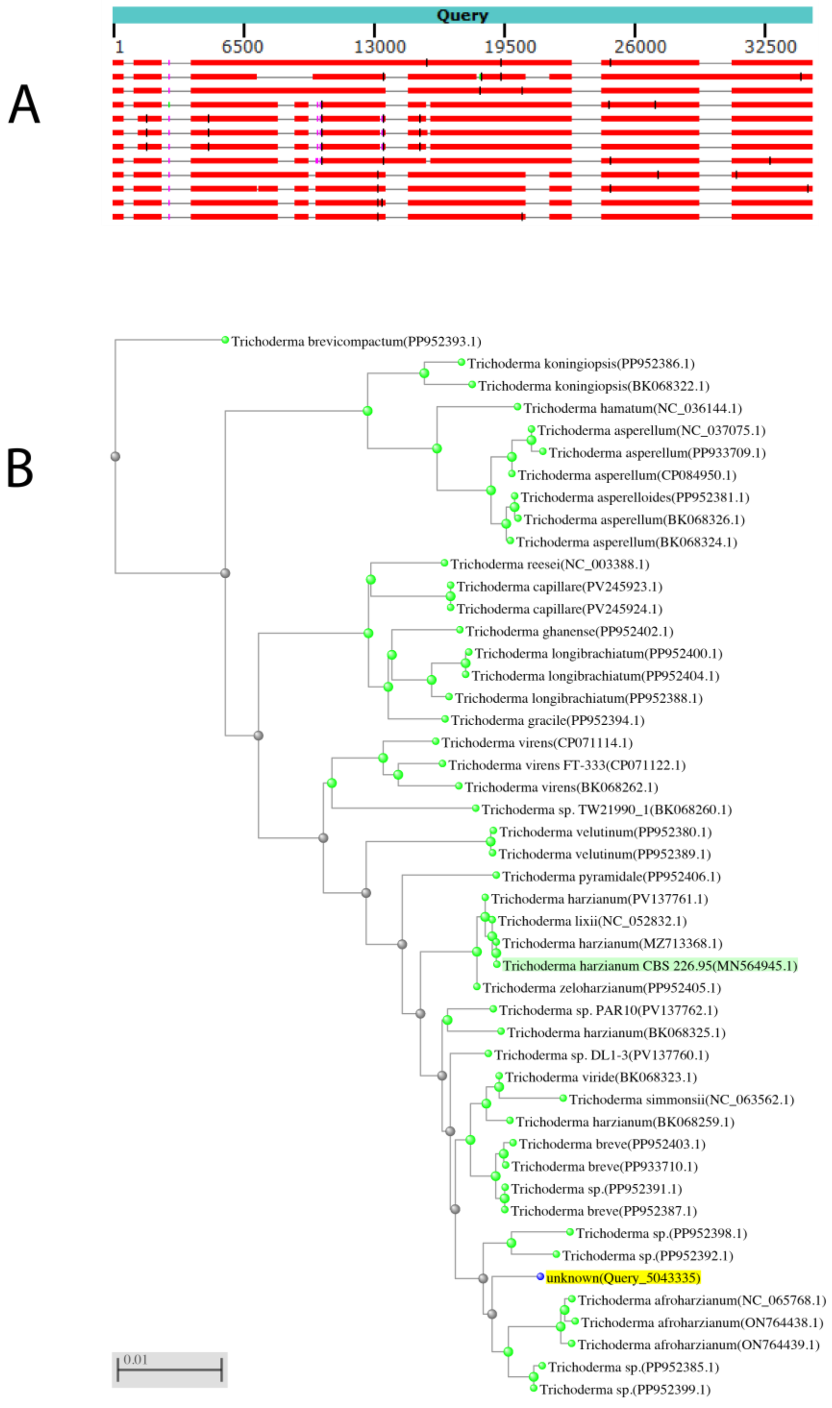

Figure S3. (A) Graphic summary results from BLASTn obtained by aligning mitochondrial DNA to base nt (top 50 hits); (B) Tree obtained in BLAST for top 50 hits (fast maximum evolution method, Max seq difference 0.75).

Table S3. Sequences used in the phylogenetic analysis.

Species	Strain	ITS	tef1	rpb2
T. acremonioides	11591		MH612375	MH612369
T. adaptatum	TC408		KX428024	KX428042
T. aeroaquaticum	NBRC 108034	NR_134333	AB646530	AB646526
T. aeroaquaticum	NBRC 108035	AB646523	AB646531	AB646527
T. aerugineum	CBS 120541	NR_134379	FJ860608	FJ860516
T. aethiopicum	CBS 130628	MH865819	EU401615	HM182986
T. aethiopicum	PPRC H5		EU401616	
T. aethiopicum	PPRC J11		EU401614	
T. afarasin	CBS 130755	NR_137301	AF348093	
T. afarasin	DIS377A		FJ463322	FJ442799
T. afarasin	DIS314F	FJ442259	FJ463400	FJ442778
T. afroharzianum	G.J.S. 04-186	FJ442265	FJ463301	FJ442691
T. afroharzianum	G.J.S. 00-24	AF443922	AF443940	FJ442726
T. aggregatum	TC927	NR_154582	KY688062	KY688001
T. aggressivum	DAOM 222156	AF443911	AF348098	FJ442752
T. aggressivum	CBS 450.95	KP009379	KP008999	KP009170
T. aggressivum f. sp. europaeum	CBS 433.95	FJ442605	AF348097	FJ442704
T. aggressivum f. sp. europaeum	CBS 435.95	KP009305	KP008998	KP009169
T. aggressivum f. sp. europaeum	CBS 100526	NR_145035	KP008993	KP009166
T. aggressivum f. sp. europaeum	CBS 100525	AF057600	AF348095	AF545541
T. albocorneum	G.J.S. 97-28		AY937440	
T. albofulvopsis	9930		KU529127	KU529138
T. albofulvum	G.J.S. 01-265		DQ835494	KR094870
T. albolutescens	CBS 119286	NR_134380	FJ860609	FJ860517
T. albolutescens	CBS 131489		KJ665354	KJ665240
T. alboviride	TC916		MF371230	MF371215
T. alcalifuscescens	TFC2000-36	NR_134381	FJ860610	
T. alcalifuscescens	TFC181548		DQ834455	DQ834462
T. alni	CBS 120633	EU518651	EU498312	EU498349
T. alni	C.P.K. 2494	EU518652	EU498313	EU498350
T. alpinum	TC20	NR_154561	KY688012	KY687958
T. alutaceum	CBS 120535	NR_134382	FJ179567	FJ179600
T. alutaceum	CBS 332.69	FJ860724	FJ179568	FJ179601

T. amazonicum	CBS 126898	NR_111529	HM142376	HM142367
T. amazonicum	IB46	HM142355	HM142374	HM142365
T. americanum	G.J.S. 92-93	DQ835410	DQ835489	DQ835455
T. americanum	G.J.S. 94-79	DQ835408	DQ835432	DQ835456
T. andinense	G.J.S. 09-62		JN175587	JN175533
T. andinense	G.J.S. 90-140	X93957	AY956321	JN175531
T. angustum	9572		KX026959	KX026967
T. appalachiense	G.J.S. 97-243	NR_134340	DQ307503	
T. appalachiense	G.J.S. 00-67	DQ315418	DQ307502	
T. appalachiense	TC226		MF095875	MF095868
T. applanatum	7792	NR_134443	KJ634759	KJ634726
T. applanatum	7781	KJ783289	KJ634757	KJ634724
T. arundinaceum	ATCC 90237	EU330927	EU338291	EU338326
T. arundinaceum	G.J.S. 05-183	EU330926	EU338274	EU338302
T. asperelloides	G.J.S. 04-116	GU198301	GU248412	GU248411
T. asperelloides	G.J.S. 04-187	JN133553	JN133571	JN133560
T. asperellum	CBS 433.97	AF278788	AF456907	EU248617
T. asperellum	G.J.S. 90-7	EU330956	EU338333	EU338337
T. asperellum	CBS 433.97	NR_130668		
T. asterineum	8892		KT224465	KT224469
T. atlanticum	CBS 120632	NR_134397	FJ860649	FJ860546
T. atlanticum	C.P.K. 1896	FJ860780	FJ860648	FJ860545
T. atrobrunneum	G.J.S. 05-101	FJ442677	FJ463392	FJ442745
T. atrobrunneum	G.J.S. 90-254	AF443926	AF443943	FJ442735
T. atrobrunneum	BPI 802854	NR_137298	AF443942	
T. atrobrunneum	CBS 130440	FJ442273	FJ463360	FJ442724
T. atrobrunneum	S3		KJ665376	KJ665241
T. atrogelatinosum	CBS 237.63	AF400751		KJ842201
T. atrogelatinosum	DAOM 167632		KJ871083	
T. atrogelatinosum	LU498		KJ871087	KJ842176
T. atroviride	CBS 142.95	AF456917	AF456891	EU341801
T. atroviride	CBS 119499	FJ860726	FJ860611	FJ860518
T. attinorum	LESF 236	NR_137307	KT279039	KT278971
T. auranteffusum	CBS 119284	FJ860728	FJ860613	FJ860520
T. auranteffusum	C.P.K. 3119	FJ860730	FJ860614	FJ860521
T. aureoviride	CBS 120536	FJ860732		FJ179602
T. aureoviride	C.P.K. 2849		FJ860616	
T. aureoviride	C.P.K. 2848	FJ860733	FJ860615	JQ685882
T. austriacum	CBS 122494	FJ860735	FJ860619	FJ860525

T. austriacum	CBS 122770	FJ860734	FJ860618	
T. austrokoningii	G.J.S. 99-146	DQ323423	DQ307561	
T. austrokoningii	CBS 119092			KJ842161
T. austrokoningii	CBS 247.63	DQ315470	DQ307568	FJ442772
T. avellaneum	CTR77-155		AY225857	AF545562
T. balearicum	CBS 133222		KJ665434	KJ665242
T. bannaense	TC564	NR_154570	KY688037	KY687979
T. barbatum	G.J.S. 04-308	HQ342417	HQ342223	HQ342286
T. barbatum	DAOM 230008	HQ342418	HQ342224	HQ342287
T. bavaricum	CBS 120538	FJ860737	FJ860621	FJ860527
T. bavaricum	C.P.K. 2021	FJ860736	FJ860620	FJ860526
T. beijingense	TC727		KX428025	KX428043
T. beinartii	PPRI 19281	KX267803	KX267782	
T. bifurcatum	TC162		KX428018	KX428036
T. bissettii	UTHSC:08-2443	NR_134442	HG931266	
T. bissettii	S43			KJ665243
T. bissettii	UTHSC:07-2998	KJ174233	HG931264	
T. breve	TC735	NR_154574	KY688045	KY687983
T. brevicompactum	G.J.S. 04-381	EU330941	EU338299	EU338317
T. brevicompactum	CBS 112444	EU330938	EU338296	EU338314
T. brevicompactum	TUBF-1076	NR_138434	AY857297	DQ857347
T. brevicrassum	TC967	NR_154583	KY688064	KY688008
T. britannicum	SB1	NR_160088	KF134796	KF134787
T. britdaniae	WU31610	NR_138454	JQ685866	JQ685880
T. britdaniae	8157	KJ783306	KJ634774	KJ634741
T. brunneoviride	CBS 121130	EU518659	EU498316	EU498357
T. brunneoviride	CBS 120928	EU518661	EU498318	EU498358
T. byssinum	TC554	NR_154569	KY688035	KY687977
T. caeruleimontis	PPRI 23903	KX267813	KX267792	
T. caerulescens	S195	JN715589	JN715621	JN715604
T. caerulescens	S2	JN715591	JN715615	JN715602
T. caesareum	G.J.S. 01-225	HQ342410	HQ342216	HQ342279
T. calamagrostidis	CBS 121133	FJ860739	FJ860622	FJ860528
T. camerunense	CBS 138272	NR_137300	AF348107	
T. camerunense	G.J.S. 99-231	AY027783	AF348108	
T. capillare	G.J.S. 06-66		JN175585	JN175530
T. capillare	C.P.K. 2883		JN182283	JN182312

T. caribbaeum	CBS 119093	NR_166015	KJ665443	KJ665246
T. caribbaeum	G.J.S. 98-43	DQ313139	DQ284976	FJ442723
T. caribbaeum var.aequatoriale	Dis320c	DQ323436	DQ289010	KT028596
T. catoptron	G.J.S. 02-76	AY737766	AY737726	AY391900
T. catoptron	DAOM 232830		KJ871245	KJ842166
T. ceciliae	CBS 130010	NR_160253	KJ665444	KJ665247
T. centrosinicum	9828		KX066257	KX066267
T. ceraceum	G.J.S. 95-159	EU330953	AY937437	AF545508
T. ceraceum	G.J.S. 88-28		AY391964	AY391901
T. ceramicum	CBS 114576	FJ860743	FJ860628	FJ860531
T. ceramicum	S353		KJ665445	KJ665248
T. cerebriforme	G.J.S. 85-245	NR_134447	KP109824	
T. cerinum	ND	NR_111835		
T. cerinum	S357		KF134797	KF134788
T. cerinum	DAOM 230012		KJ871242	KJ842184
T. changbaiense	TC 610		MF371224	MF371209
T. chetii	PPRI 19363	KX267805	KX267784	
T. chlamydosporicum	TC 794	NR_154577	KY688052	KY687989
T. chlorosporum	G.J.S 98-1	AY737762	AY391968	AY391906
T. chlorosporum	G.J.S. 88-33		AY391966	AY391903
T. christiani	CBS 132572		KJ665439	KJ665244
T. christiani	S93		KJ665442	KJ665245
T. chromospermum	G.J.S. 94-67	AY737774	AY391973	AY391912
T. chromospermum	G.J.S. 94-68		AY391974	AY391913
T. cinnamomeum	G.J.S. 97-237	AY737759	AY391979	AY391920
T. cinnamomeum	G.J.S. 97-233		AY391978	AY391919
T. citrinoviride	DAOM 172792	NR_077178	EU280036	KJ842210
T. citrinoviride	CBS 121275	FJ860830		FJ860586
T. citrinoviride	CBS 258.85	MH861877	AY865637	
T. citrinoviride	S20		KJ665449	KJ665250
T. citrinum	CBS 894.85	NR_134368	DQ835481	AF545561
T. citrinum	G.J.S. 89-145	DQ000622	DQ835438	DQ835457
T. compactum	CBS 121218	NR_138435	KF134798	KF134789
T. composticola	CBS 439.95	DQ315439	AY937413	KC285753
T. composticola	CBS 438.95	DQ315438	DQ307522	EU341806
T. composticola	CBS 133497		KC285631	KC285754
T. concentricum	TC295	NR_154564	KY688027	KY687971
T. confertum	TC139		MF371220	MF371205

T. confluens	9649		KT001959	KT001964
T. corneum	G.J.S. 97-82		KJ665455	KJ665252
T. costaricense	PC21	AY737754	AY391980	AY391921
T. crassum	DAOM 164916	NR_134370	EU280048	KJ842185
T. crassum	TRS113	KP009300	KP008865	KP009102
T. crassum	DAOM 164916			AF545542
T. cremeoides	S112		KJ665456	KJ665253
T. cremeoides	S192		KJ665460	KJ665254
T. cremeum	G.J.S. 91-125	AY737760	AF534598	AF545511
T. croceum	DAOM 167068	AF400259	AY750879	
T. crystalligenum	CBS 118980	DQ344490	DQ345342	DQ345347
T. crystalligenum	Hypo167		DQ345344	DQ345348
T. cyanodichotomus	CGMCC 12161	KY189312	KY707174	KY707175
T. dacrymycellum	WU29044	FJ860749	FJ860633	FJ860533
T. danicum	CBS 121273	FJ860750	FJ860634	FJ860534
T. danicum	S553		KJ665465	KJ665255
T. decipiens	G.J.S. 91-101	JN133554		
T. decipiens	CBS 121307		FJ860635	
T. decipiens	S372		KJ665466	KJ665256
T. delicatulum	CBS 120631	FJ860751	FJ860636	FJ860535
T. deliquescens	CBS 121131	FJ860771	FJ860644	FJ179609
T. densum	10165		KU529126	KU529137
T. dimorphum	TC614		MF371225	MF371210
T. dingleyae	CBS 119056	NR_138443	KJ665467	KJ665257
T. dingleyae	G.J.S. 99-105	DQ313151	AF348117	EU341803
T. dorotheae	G.J.S. 99-202	MH863050	DQ307536	EU248602
T. dorotheae	S231		KJ665468	KJ665258
T. effusum	DAOM 230007	DQ083008	KJ665473	KJ665260
T. effusum	TUBF-354	AF149858	AF510432	
T. eijii	TUFC 100002	JX238476	JX684011	JX238484
T. eijii	8139	KJ783301	KJ634769	KJ634736
T. endophyticum	CBS 130730	FJ442242	FJ463314	FJ442721
T. endophyticum	CBS 130729	FJ442243	FJ463319	
T. epimyces	CBS 120534	NR_134377	EU498320	EU498360
T. epimyces	C.P.K. 2487	EU518665	EU498322	EU498361
T. epimyces	Hypo175	EU518662	EU498319	EU498359
T. erinaceus	DAOM 230019	NR_111837	AY750880	EU248603
T. erinaceus	DIS7	DQ109534	DQ109547	EU248604
T. estonicum	G.J.S. 96-129	AY737767	FJ860638	AF545514

T. estonicum	ND	FJ860754		
T. estonicum	CBS 121556		FJ860637	FJ860536
T. eucorticioides	G.J.S. 99-61	JN133555	DQ835474	DQ835518
T. eucorticioides	LESF208	KT278867	KT279045	KT278973
T. europaeum	Hypo64		KJ665476	KJ665264
T. europaeum	S134		KJ665481	KJ665265
T. europaeum	CBS 121276		FJ179574	FJ179610
T. euskadiense	CBS 130013		KJ665492	KJ665269
T. evansii	DIS341HI	EU883568	EU883566	EU883558
T. evansii	Dis282d	EU856294	EU856319	FJ150784
T. fassatiae	CBS 140105	LN866275	LN866277	LN866276
T. fertile	DAOM 167161	NR_134336	AF534618	AF545546
T. fertile	DAOM 167070		AF534617	AF545545
T. flagellatum	C.P.K. 3334		FJ763149	JN258688
T. flagellatum	C.P.K. 3525	MH865822	FJ763184	KR297247
T. flavescens	HMJAU34730		KU235485	KU235486
T. flaviconidium	G.J.S. 99-49	DQ023301	AY665710	EU883557
T. flaviconidium	G.J.S. 99-57	AY665701	AY665711	
T. flavipes	G.J.S. 92-102		DQ834454	DQ834461
T. floccosum	G.J.S. 01-238	HQ342412	HQ342218	HQ342281
T. floccosum	TC633		KX266245	KX266251
T. foliicola	Hypo645	JQ685871	JQ685862	JQ685876
T. foliicola	Hypo650	JQ685872	JQ685863	JQ685877
T. fomiticola	CBS 121136	NR_134391	FJ860639	FJ860538
T. fomiticola	C.P.K. 3137	FJ860756	FJ860640	FJ860539
T. fructicola	HMAS 275663		MG383490	MG383484
T. fujianense	230		MF374811	MF374808
T. gamsii	G.J.S. 04-09	DQ315459	DQ307541	JN133561
T. gamsii	G.J.S. 05-111	DQ841730	DQ841722	KT028597
T. gamsii	FMR12636	NR_131317	HG931239	
T. ganodermatis	TC877	NR_154581	KY688060	KY687995
T. gelatinosum	G.J.S. 98-184		AY391983	AY391924
T. gelatinosum	C.P.K. 1618		FJ179569	FJ179604
T. gelatinosum	G.J.S. 88-17	NR_138433	AF534579	AF545516
T. ghanense	DAOM 220800	EU280100	EU280043	KJ842206
T. ghanense	G.J.S. 95-137	NR_120299	AY937423	JN175559
T. gillesii	G.J.S. 00-72		JN175583	JN175527
T. gliocladium	CBS 130009	NR_160252	KJ665502	KJ665271
T. gliocladium	S83		KJ665503	KJ665272

T. globoides	10648		KX026955	KX026963
T. gracile	G.J.S. 10-263		JN175598	JN175547
T. grande	10042		KX066254	KX066266
T. gregarium	TC11		MF371217	MF371202
T. guizhouense	HGUP0038	JN191311	JN215484	JQ901400
T. guizhouense	HGUP0039	JX089584	JX089585	JQ901401
T. guizhouense	S628		KJ665511	KJ665273
T. hainanense	TC468	NR_154568	KY688033	KY687976
T. hamatum	DAOM 167057	EU280124	AF534620	AF545548
T. hamatum	G.J.S. 05-334	EU856291	EU856316	FJ150781
T. harzianum	CBS 226.95	AY605713	AF348101	AF545549
T. harzianum	G.J.S. 05-107	FJ442679	FJ463329	FJ442708
T. hausknechtii	CBS 133493		KJ665515	KJ665276
T. hebeiense	TC472	NR_153278	KX344434	KX344439
T. helicolixii	CBS 133499		KJ665517	KJ665278
T. helicolixii	CBS 135583		KJ665516	KJ665277
T. helicum	DAOM 230016	DQ083022	EU280055	KJ842200
T. helicum	DAOM 230021		KJ871125	DQ087239
T. henanense	8889		KT224464	KT224467
T. hengshanicum	TC842	NR_154578	KY688054	KY687991
T. hirsutum	TC334	NR_154565	KY688029	KY687972
T. hispanicum	S453	JN715595	JN715659	JN715600
T. hispanicum	S172	JN715597	JN715655	
T. hongkongense	HMAS 273832	NR_154954	KX495364	KX980154
T. hubeiense	9493		KT001956	KT001961
T. hunanense	TC579	NR_154571	KY688039	KY687980
T. hunua	CBS 238.63	NR_160091	KJ665519	KJ665279
T. hypoxylon	CGMCC 3.17906	NR_153269	KU974001	KX500395
T. ingratum	TC34	NR_154566	KY688018	KY687973
T. inhamatum	CBS 273.78	FJ442680	AF348099	FJ442725
T. intricatum	G.J.S. 02-78	EU264002	EU248630	EU241505
T. intricatum	G.J.S. 97-88	NR_134343	AY376060	
T. istrianum	CBS 130539		KJ665523	KJ665281
T. istrianum	S123		KJ665521	KJ665280
T. italicum	CBS 132567		KJ665525	KJ665282
T. italicum	S15		KJ665526	KJ665283
T. ivoriense	G.J.S. 01-312	HQ342411	HQ342217	HQ342280
T. junci	CBS 120926	NR_134392	FJ860641	FJ860540

T. konilangbra	G.J.S. 96-147	DQ083021	AY937425	
T. konilangbra	C.P.K. 132		JN258681	KJ665284
T. koningii	ATCC 64262	NR_138456	AY376046	
T. koningii	G.J.S. 00-168			FJ442761
T. koningii	G.J.S. 90-18	DQ323409	DQ289007	EU248600
T. koningii	Hypo51		KC285594	FJ860541
T. koningiopsis	G.J.S. 97-273	DQ109537	FJ463277	FJ442795
T. koningiopsis	DAOM 222105	DQ313146	AY376042	EU341810
T. koningiopsis	G.J.S. 93-20	NR_131281	DQ284966	EU241506
T. kunigamense	TAMA193	NR_134334	AB807645	AB807657
T. kunmingense	YMF1.02659	KJ742800	KJ742802	KJ742801
T. lacuwombatense	G.J.S. 99-198	NR_134356	KJ665547	KJ665286
T. lacuwombatense	DAOM 231833		KJ871228	KJ842157
T. laevisporum	9481		KU529128	KU529139
T. lanuginosum	G.J.S. 01-176	NR_134429	HQ342221	HQ342284
T. lanuginosum	G.J.S. 01-174	HQ342414	HQ342220	HQ342283
T. leguminosarum	S494		KJ665551	KJ665288
T. leguminosarum	S503		KJ665552	KJ665289
T. lentiforme	G.J.S. 00-22	AF443912	AF443930	FJ442687
T. lentiforme	DIS94D	FJ442615	FJ463379	
T. lentiforme	BPI744709	NR_144868	AF469195	
T. leucopus	CBS 122499	FJ860764	FJ179571	FJ179605
T. leucopus	CBS 122495	FJ860765	FJ179570	FJ179606
T. liberatum	TC253	NR_154563	KY688025	KY687969
T. lieckfeldtiae	G.J.S. 00-14	DQ109528	EU856326	EU883562
T. lieckfeldtiae	G.J.S. 04-196	EU856299	EU856324	FJ150790
T. limonium	10037		KX066247	KX066259
T. linzhiense	TC742	NR_154575	KY688047	KY687985
T. lixii	CBS 110080	NR_131264	AF443938	KJ665290
T. longibrachiatum	CBS 816.68	EU401556	EU401591	DQ087242
T. longibrachiatum	DAOM 166989	EU330961	EU338335	EU338339
T. longibrachiatum	CBS 816.68	NR_120298	AY865640	HQ260615
T. longibrachiatum	S328	JQ685875	JQ685867	JQ685883
T. longifialidicum	LESF552	NR_137309	KT279020	KT278955
T. longipile	DAOM 177227	NR_134354	AF534622	AF545550
T. longipile	CBS 120953	FJ860770	FJ860643	FJ860542
T. longisporum	TC673	NR_154573	KY688043	KY687982
T. luteffusum	CBS 120537	FJ860773	FJ860645	FJ860543
T. luteocrystallinum	CBS 123828	NR_134396	FJ860646	FJ860544

T. lycogaloides	CBS 123493		KF134800	KF134792
T. mangshanicum	TC860		KX428032	KX428050
T. margaretense	C.P.K. 3127	FJ860741	FJ860625	FJ860529
T. margaretense	C.P.K. 3128		FJ860626	FJ860530
T. martiale	G.J.S. 04-40	NR_134363	EU248618	EU248597
T. matsushimae	IMI 266915		AB646539	
T. matsushimae	IMI		AB646534	
T. mediterraneum	CBS 136469		KJ665568	KJ665296
T. mediterraneum	S12		KJ665562	KJ665293
T. medogense	11058		MG383491	MG383486
T. medusae	G.J.S. 01-171	NR_134426	HQ342214	HQ342277
T. medusae	G.J.S. 01-166	HQ342409	HQ342215	HQ342278
T. megalocitrinum	BEO00-09	DQ835511	AY225855	AF545563
T. melanomagnum	G.J.S. 99-153	AY737770	AY391985	AY391926
T. microcitrinum	G.J.S. 91-61	DQ835426	DQ835478	DQ835460
T. microcitrinum	G.J.S. 97-248	DQ835424	DQ835479	DQ835462
T. mienum	CBS 132690	NR_134433	AB856681	AB856754
T. mienum	TUFC 61517	JQ621974	JQ621975	JQ621965
T. minutisporum	DAOM 167069	NR_111192	KJ665612	KJ665314
T. minutisporum	CBS 112255		KJ665618	KJ665316
T. moravicum	CBS 120539	NR_134398	FJ860651	
T. moravicum	C.P.K. 2419			FJ860548
T. moravicum	C.P.K. 2489	FJ860783		FJ860549
T. neocrassum	BPI 843647		JN133572	AY481587
T. neokoningii	CBS 120070	NR_138446	KJ665620	KJ665318
T. neorufoides	CBS 119506	NR_134399	FJ860657	
T. neorufoides	C.P.K. 1900			FJ860553
T. neorufoides	C.P.K. 1904	FJ860790	FJ860658	FJ860554
T. neorufum	CBS 119498		FJ860653	FJ860550
T. neorufum	C.P.K. 1613	FJ860785	FJ860655	FJ860551
T. neorufum	CBS 111144	NR_077132	AF487670	KJ842140
T. neosinense	G.J.S. 94-11	NR_134362	KJ665624	KC285777
T. neosinense	G.J.S. 94-9	DQ315421	DQ307507	EU241496
T. neotropicale	PP136	FJ884107	FJ967803	
T. neotropicale	LA11	HQ022407	HQ022771	
T. nothescens	G.J.S. 99-142	DQ315427	DQ307512	EU241498
T. nothescens	G.J.S. 99-86		DQ307516	EU241497
T. novae-zelandiae	CBS 639.92	NR_111193	AY937448	JN133563
T. novae-zelandiae	G.J.S. 99-113		JN175582	JN175526

T. nybergianum	CBS 122500	NR_134400	FJ179575	FJ179611
T. nybergianum	CBS 122496	FJ860792	FJ179576	FJ179612
T. oblongisporum	DAOM 167085	NR_138437	AF534623	AF545551
T. oblongisporum	DAOM 176226		KJ871142	KJ842199
T. ochroleucum	CBS 119502	NR_134401	FJ860659	FJ860556
T. odoratum	10035		KT224463	KT224468
T. oligosporum	7890	KJ783296	KJ634764	KJ634731
T. oligosporum	7718	KJ783283	KJ634751	KJ634718
T. olivascens	CBS 119322	DQ677650		
T. olivascens	S34		KC285615	KC285751
T. olivascens	S475		KC285624	KC285752
T. orientale	S187	JQ685873	JQ685868	JQ685884
T. orientale	G.J.S. 09-784		JN175578	JN175522
T. orientale	G.J.S. 88-81	NR_111317	EU401581	
T. ovalisporum	G.J.S. 04-113	FJ442614	FJ463281	FJ442781
T. ovalisporum	Dis70a	AY380897	AY376037	FJ442742
T. pachypallidum	CBS 122126	NR_134402	FJ860662	JQ685879
T. pachypallidum	CBS 120533	FJ860797		FJ860559
T. palidulum	HMAS 275665		MG383493	MG383487
T. parapiluliferum	G.J.S. 91-60	NR_134341	AY937444	
T. parapiluliferum	CBS 112264			KJ842158
T. parapiluliferum	CBS 120921	FJ860799	FJ179578	FJ179614
T. parareesei	CBS 125925	NR_138453	GQ354353	HM182963
T. parareesei	G.J.S. 07-26		GQ354373	HM182966
T. pararogersonii	CBS 133496		KJ665625	KJ665320
T. paratroviride	CBS 136489		KJ665627	KJ665321
T. paratroviride	S489		KJ665628	KJ665322
T. paraviridescens	CBS 119321	NR_134367	DQ672610	KC285763
T. paraviridescens	CBS 274.79	DQ315428	DQ307513	EU252010
T. parepimyces	CBS 122769	NR_134403	FJ860664	FJ860562
T. parepimyces	CBS 122768	FJ860801	FJ860665	FJ860563
T. parestonicum	CBS 120636	NR_134404	FJ860667	FJ860565
T. parestonicum	C.P.K. 2427	FJ860802	FJ860666	FJ860564
T. parmastoi	TFC97-143	NR_134405	FJ860669	DQ834463
T. parmastoi	CBS 121139		FJ860668	FJ860567
T. patella	G.J.S. 91-141	NR_134338	KJ665630	KJ665323
T. patellotropicum	BPI744562	NR_134339	AY937428	
T. paucisporum	G.J.S. 01-13	NR_134360	DQ109540	FJ150787
T. paucisporum	G.J.S. 03-69	DQ109527	DQ109541	EU883560

T. peltatum	G.J.S. 08-207	NR_134422	KR135819	HQ260610
T. peltatum	J.D.Rogers1	EF392732	EF392731	EF392733
T. perviride	9757		KX026954	KX026962
T. petersenii	CBS 119507	FJ860806	FJ860670	FJ860568
T. petersenii	G.J.S. 04-164	DQ323442	DQ289004	FJ442783
T. petersenii	G.J.S. 04-355	DQ323441	DQ284980	
T. pezizoides	G.J.S. 01-257	DQ000632	AY937438	EU248608
T. phellinicola	CBS 119283	NR_134406	FJ860672	FJ860569
T. phyllostachydis	CBS 114071	NR_103608	FJ860673	FJ860570
T. phyllostachydis	G.J.S. 92-81		AY391986	AY391927
T. piluliferum	CBS 120927	FJ860810	FJ860674	FJ179615
T. piluliferum	C.P.K. 3143	FJ860811	FJ179579	FJ860571
T. pinnatum	G.J.S. 04-100		JN175571	JN175515
T. pinnatum	G.J.S. 02-120		JN175572	JN175516
T. placentula	CBS 120924	NR_134408	FJ179580	FJ179616
T. placentula	CBS 121134	FJ860815		FJ860572
T. pleuroti	CBS 124387	NR_134421	HM142382	HM142372
T. pleuroti	C.P.K. 2117	EU280069	EU279975	
T. pleuroticola	CBS 124383	NR_134420	HM142381	HM142371
T. pleuroticola	G.J.S. 95-81	AF345948	AF348102	
T. polyalthiae	TBRC8737	MF135132	LC328976	LC373011
T. polypori	TC876	NR_154580	KY688058	KY687994
T. polysporum	CBS 820.68	NR_134448	AY605810	DQ087238
T. polysporum	C.P.K. 3131		FJ860661	JQ685878
T. polysporum	S258		KJ665677	KJ665330
T. poronioideum	G.J.S. 01-203	NR_134446	KP109823	
T. priscilae	CBS 131487		KJ665691	KJ665333
T. priscilae	S129		KJ665689	KJ665332
T. protopulvinatum	CBS 739.83	NR_134409	FJ860679	DQ835463
T. protopulvinatum	C.P.K. 2434		FJ860677	FJ860574
T. protrudens	DIS119F	EU330946	EU338289	EU338322
T. pruinosum	TC864		MF371227	MF371212
T. pseudobritdaniae	8663		KT224462	KT224466
T. pseudocandidum	PC59	AY737757	AY391962	AY391899
T. pseudodensum	TC222	NR_154562	KY688023	KY687967
T. pseudogelatinosum	TUFC 60186	NR_144878	JQ797397	JQ797405
T. pseudogelatinosum	CNUN309	HM769754	HM920202	HM920173
T. pseudokoningii	DAOM 167678	NR_120296	KJ713204	KJ842214
T. pseudokoningii	G.J.S. 81-300	DQ083025	AY937429	JN175534

T. pseudolacteum	TUFC 61490	JX238469	JX238493	JX238478
T. pseudolacteum	TUFC 61509	JX238473	JX238497	JX238482
T. pseudonigrovirens	G.J.S. 99-64	NR_134353	AF534582	AF545518
T. pseudonigrovirens	DAOM 232837		KJ871128	KJ842193
T. pseudostramineum	TUFC 60104	NR_134435	JQ797400	JQ797408
T. psychrophilum	C.P.K. 1602	FJ860818	FJ860680	FJ860575
T. psychrophilum	HY8	EU330957	AF534584	AF545520
T. psychrophilum	CBS 119129		FJ860681	
T. pubescens	DAOM 166162	NR_077179	AF534624	EU248613
T. pubescens	G.J.S. 01-207	EU856280	EU856304	FJ150768
T. pubescens	DAOM 166162		EU279963	EU248613
T. pulvinatum	CBS 121279	NR_134410	FJ860683	FJ860577
T. pulvinatum	G.J.S. 98-104	AF487661	DQ835490	AF545559
T. purpureum	7870		KX026953	KX026961
T. pyramidale	CBS 135574		KJ665699	KJ665334
T. reesei	DAOM 167654	NR_120297	KJ713193	KJ842213
T. reesei	ATCC 24449	DQ000625	DQ025754	HQ260618
T. reesei	G.J.S. 00-89		JN175599	JN175548
T. reesei	QM6a	MH861154	Z23012	HM182969
T. restrictum	PPRI 19367	KX267815	KX267794	
T. rhododendri	CBS 119288	NR_134411	FJ860685	FJ860578
T. rifaii	DIS337F	FJ442621	FJ463321	FJ442720
T. rifaii	DIS355B	FJ442663	FJ463324	
T. rodmanii	CBS 121553	FJ860824	FJ860687	FJ860580
T. rodmanii	G.J.S. 91-88	NR_134374	EU338286	EU338324
T. rogersonii	CBS 119503	FJ860826	FJ860690	FJ860583
T. rogersonii	G.J.S. 04-157	DQ323415	DQ307558	JN133566
T. rossicum	DAOM 230011	HQ342419	AY937441	HQ342288
T. rossicum	G.J.S. 07-72	HQ342416	HQ342222	HQ342285
T. rosulatum	7752	NR_134438	KF729984	KF730005
T. rubi	CBS 127380		KJ665704	KJ665336
T. rufobrunneum	8155	NR_134439	KF729992	KF730007
T. rufobrunneum	8084	KF729998	KF729989	KF730010
T. rugosum	11325		MH612379	MH612373
T. sambuci	WU29467	NR_134412	FJ860693	FJ860585
T. samuelsii	S5	NR_138452	JN715651	JN715599
T. samuelsii	S42	JN715593	JN715652	JN715598
T. saturnisporopsis	S19	NR_138455	JQ685869	JQ685885
T. saturnisporopsis	TR175			DQ857348

T. saturnisporum	CBS 330.70	NR_103704	EU280044	DQ087243
T. saturnisporum	ATCC 28023		JN388897	JN175524
T. scalesiae	G.J.S. 03-74	NR_144876	DQ841726	EU252007
T. semiorbis	G.J.S. 99-108	NR_134423	JN133576	JN133567
T. semiorbis	DAOM 167636	AY737758	KJ871120	AF545522
T. sempervirentis	S599		KC285632	KC285755
T. sempervirentis	S601		KC285633	KC285756
T. seppoi	CBS 122498	NR_134413	FJ179581	FJ179617
T. seppoi	CBS 122497	FJ860834	FJ179582	FJ179618
T. shaoguanicum	TC826		KX428031	KX428049
T. shennongjianum	TC376	NR_144880	KT735253	KT735259
T. sichuanense	TC143	NR_153277	KX344428	KX344437
T. silvae-virgineae	CBS 120922	NR_134414	FJ860696	FJ860587
T. silvae-virgineae	C.P.K. 974	FJ860835	FJ860695	
T. simmonsii	G.J.S. 91-138	NR_137297	AF443935	FJ442757
T. simmonsii	G.J.S. 90-127	AF443918	AF443936	FJ442798
T. simmonsii	S7		KJ665719	KJ665337
T. simplex	TC671	NR_154572	KY688041	KY687981
T. sinense	DAOM 230004	NR_134425	KJ713191	JN175528
T. sinense	C.P.K. 530		JN182273	JN182310
T. sinokoningii	8849		KU529130	KU529141
T. sinoluteum	8205	NR_134445	KJ634777	KJ634744
T. sinoluteum	8210	KJ783310	KJ634778	KJ634745
T. sinuosum	PC8	NR_144872	AY391997	AY391942
T. sinuosum	C.P.K. 1595	FJ860838	FJ860697	FJ179619
T. solani	G.J.S. 08-81		JN175597	JN175546
T. solum	TC781	NR_154576	KY688050	KY687987
T. songyi	CBS 138099	MG491505	KJ636525	KJ636518
T. sp. S138	S138		KJ665730	KJ665343
T. sp. S169	S169		KJ665731	KJ665344
T. sp. S222	S222		KJ665732	KJ665345
T. sp. S404	S404		KJ665733	KJ665346
T. sp. S466	S466			KJ665734
T. sp. S467	S467			KJ665735
T. sp. S605	S605			KJ665736
T. sp. S610	S610			KJ665737
T. sp. S624	S624		KJ665738	KJ665347
T. sp. S637	S637			KJ665739
T. sparsum	10122		KU529136	KU529147

T. speciosum	YMF1.00205	MH113929	MH183184	MH155270
T. sphaerosporum	9755		KU529134	KU529145
T. spinulosum	CBS 311.50	NR_134415	FJ860701	FJ860591
T. spinulosum	CBS 121280	FJ860842	FJ860699	FJ860589
T. spirale	DAOM 183974	NR_077177	EU280049	AF545553
T. spirale	DIS311D	FJ442232	FJ463369	FJ442694
T. spirale	S212		KJ665740	KJ665348
T. stellatum	CBS 112265	DQ083011	AY937445+	KJ665349
T. stercorarium	CBS 148.85	NR_134355		EF469103
T. stilbohypoxyli	CBS 119501	FJ860846	FJ860703	FJ860593
T. stilbohypoxyli	G.J.S. 96-32	AY380915	AY376062	EU341805
T. stilbohypoxyli	G.J.S.96-30	DQ109533	DQ109546	
T. stipitatum	8152	NR_134440	KF729991	KF730012
T. stipitatum	8151	KF730002	KF729990	KF730011
T. stramineum	G.J.S. 02-84	NR_134347	AY391999	AY391945
T. stramineum	G.J.S. 02-84		AY737746	
T. stramineum	TAMA0425	AB856609	AB856675	AB856748
T. strictipile	DAOM 172827		AF534628	AF545555
T. strictipile	CBS 347.93	NR_134337	AY865644	
T. strictipile	Hypo24		FJ860704	FJ860594
T. strigosellum	G.J.S. 05-02	EU263997	EU248631	EU248607
T. strigosellum	DAOM 229937	EU280139	EU280030	KJ842147
T. strigosellum	CBS 102817	NR_134437	JQ425705	
T. strigosum	DAOM 166121	NR_103571	EU280019	AF545556
T. strigosum	DIS173k	DQ109531	DQ109545	EU248606
T. stromaticum	G.J.S. 97-183	NR_077128	AY937418	HQ342245
T. stromaticum	G.J.S. 00-02	FJ442675	FJ463361	FJ442714
T. subalni	11017		MH612377	MH612371
T. subalpinum	C.P.K. 3126	FJ860851	FJ860706	FJ860596
T. subalpinum	CBS 119128		FJ860705	FJ860595
T. subeffusum	CBS 120929	NR_134416	FJ860707	FJ860597
T. subeffusum	C.P.K. 2864	FJ860853	FJ860708	FJ860598
T. subsulphureum	M-141	DQ835509	DQ835492	DQ835522
T. subviride	8658		KU529131	KU529142
T. sulawesense	G.J.S. 85-228	NR_134344	AY392002	AY391954
T. sulphureum	G.J.S. 95-190	DQ835425	DQ835448	AF545560
T. sulphureum	C.P.K. 1593		FJ860709	FJ860599
T. sulphureum	G.J.S. 95-176	NR_144869	DQ835498	
T. surrotundum	G.J.S. 88-73	NR_134350	AF534594	AF545540

T. taiwanense	G.J.S. 95-93	NR_144874	DQ284973	
T. taiwanense	C.P.K. 416			JN715608
T. tardum	TC199		KX428020	KX428038
T. tawa	G.J.S. 97-174	NR_138430	FJ463313	AY391956
T. taxi	ZJUF0986	NR_134366	DQ859029	DQ859032
T. tenue	9709		KX026952	KX026960
T. texanum	LESF551	NR_137308	KT278988	KT278920
T. thailandicum	G.J.S. 97-61	AY737772	AY392005	AY391957
T. thelephoricola	G.J.S. 95-135	NR_134352	AY392006	AY391958
T. thelephoricola	CBS 120925	FJ860858	FJ860711	FJ860600
T. theobromicola	Dis85f	NR_134359	EU856321	FJ007374
T. theobromicola	Dis376f	EU856296	EU856322	FJ150786
T. thermophilum	10129		KX066249	KX066261
T. tiantangzhaiense	7875	NR_156584	KJ634763	KJ634730
T. tibetense	TC407	NR_144881	KT735254	KT735261
T. tomentosum	DAOM 178713A	NR_134357	EU279969	AF545557
T. tomentosum	CBS 120637	FJ860744	FJ860629	FJ860532
T. tremelloides	CBS 121140	NR_134417	FJ860714	FJ860603
T. tremelloides	CBS 120634	FJ860860	FJ860713	FJ860602
T. trixiae	ATCC 32630	DQ315445	DQ307526	KC285770
T. trixiae	G.J.S. 92-11	DQ315442	DQ307524	KC285771
T. trixiae	CBS 134702	NR_138444	DQ672606	
T. tropicosinense	HMAS 252546	NR_134441	KF923286	KF923313
T. tsugarense	TAMA203	NR_134335	AB807647	AB807659
T. turrialbense	CBS 112445	NR_138448	EU338284	EU338321
T. turrialbense	BBA72294	EU330944	EU338282	EU338320
T. undatipile	TC873	NR_154579	KY688056	KY687993
T. undulatum	PPRI 19365	KX267810	KX267789	KX351319
T. valdunense	CBS 120923	NR_134418	FJ860717	FJ860605
T. velutinum	DAOM 230013		KJ665769	KF134794
T. velutinum	ATCC MYA-4841	NR_111836		
T. vermipilum	PPRI 3559	HQ342413	HQ342219	HQ342282
T. verticillatum	TC389	KY014091	KX344431	KX344438
T. victoriense	G.J.S. 99-200	NR_134369	DQ835473	DQ835517
T. victoriense	G.J.S. 99-130	EU330952	DQ835472	DQ835516
T. vinosum	G.J.S. 99-158	NR_144870	AY376047	KC285779
T. vinosum	G.J.S. 99-156	DQ315446	DQ307527	KC285778

T. virens	DAOM 167652	EU330955	AY750891	KJ842186
T. virens	CBS 249.59	AF099005	AF534631	
T. virens	CBS 249.59		AF400998	AF545558
T. virescentiflavum	PC278	NR_134349	AY392007	AY391959
T. virgineum	HMAS 275664		MG383495	MG383489
T. viridarium	G.J.S. 89-142	NR_138439	AY376049	EU241495
T. viridarium	S136		KC285658	KC285760
T. viride	G.J.S. 89-127	X93980	AF534585	AF545521
T. viride	G.J.S. 92-14	DQ313155	DQ288988	EU252006
T. viride	CBS 119325	NR_138441	DQ672615	EU711362
T. viridescens	S452		KC285646	KC285758
T. viridescens	CBS 433.34	NR_138429	AY376048	
T. viridescens	S1		KC285634	KC285757
T. viridialbum	S177		KC285705	KC285773
T. viridialbum	S250		KC285706	KC285774
T. viridulum	9767		KX026957	KX026965
T. virilente	S281		KC285692	KC285767
T. virilente	DAOM 234234	NR_138447	EU280009	
T. virilente	S517		KC285694	KC285768
T. voglmayrii	CBS 117711	NR_134358	DQ086146	FJ179622
T. voglmayrii	CBS 117710		DQ086147	DQ086151
T. vulgatum	TC194		KX428019	KX428037
T. xanthum	TC714		MF371226	MF371211
T. yui	7790b	NR_156583	KJ634758	KJ634725
T. yunnanense	CBS 121219	NR_134419	GU198243	GU198274
T. zayuense	TC442	NR_154567	KY688031	KY687974
T. zeloharzianum	YMF1.00268	MH113932	MH183181	MH158996
T. zonatum	220		MF374809	MF374806

Figure S4. Phylogenetic tree constructed by maximum likelihood mode based on *tef1+rpb2*concatenate

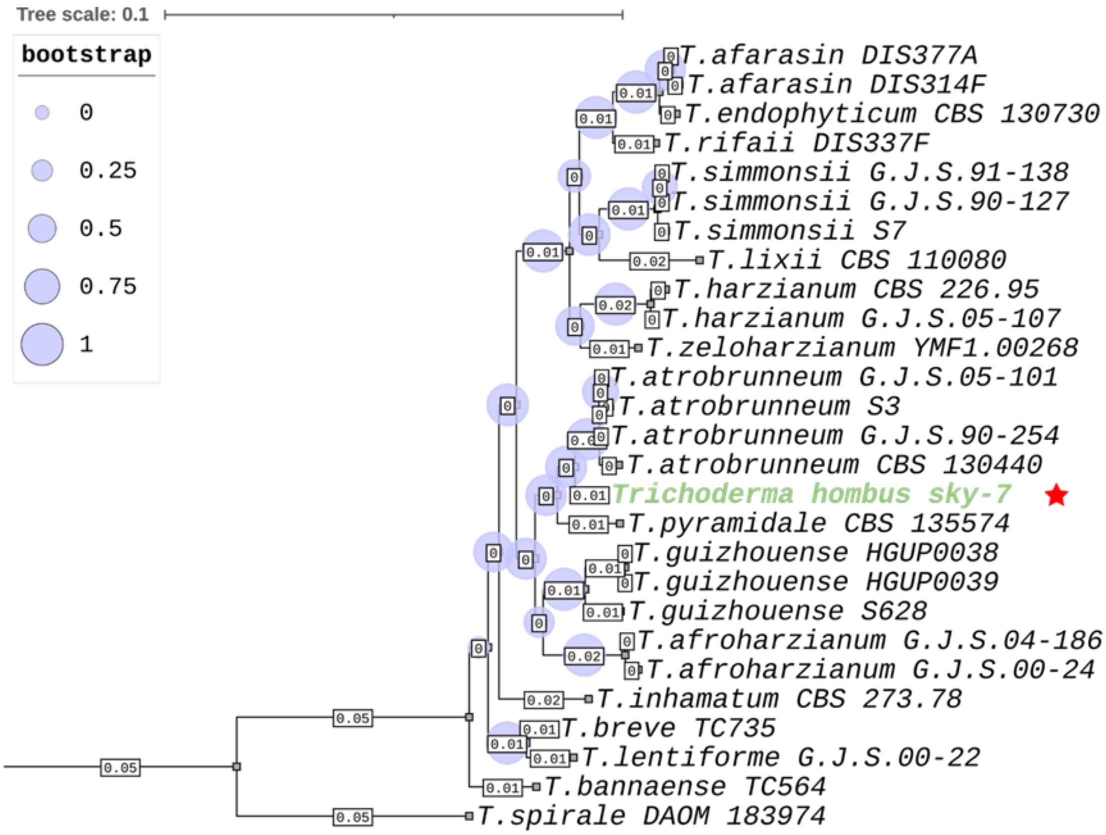

T.spirale DAOM was used as an outgroup

Figure S5. MS data for major 18-residue peptaibol **3**

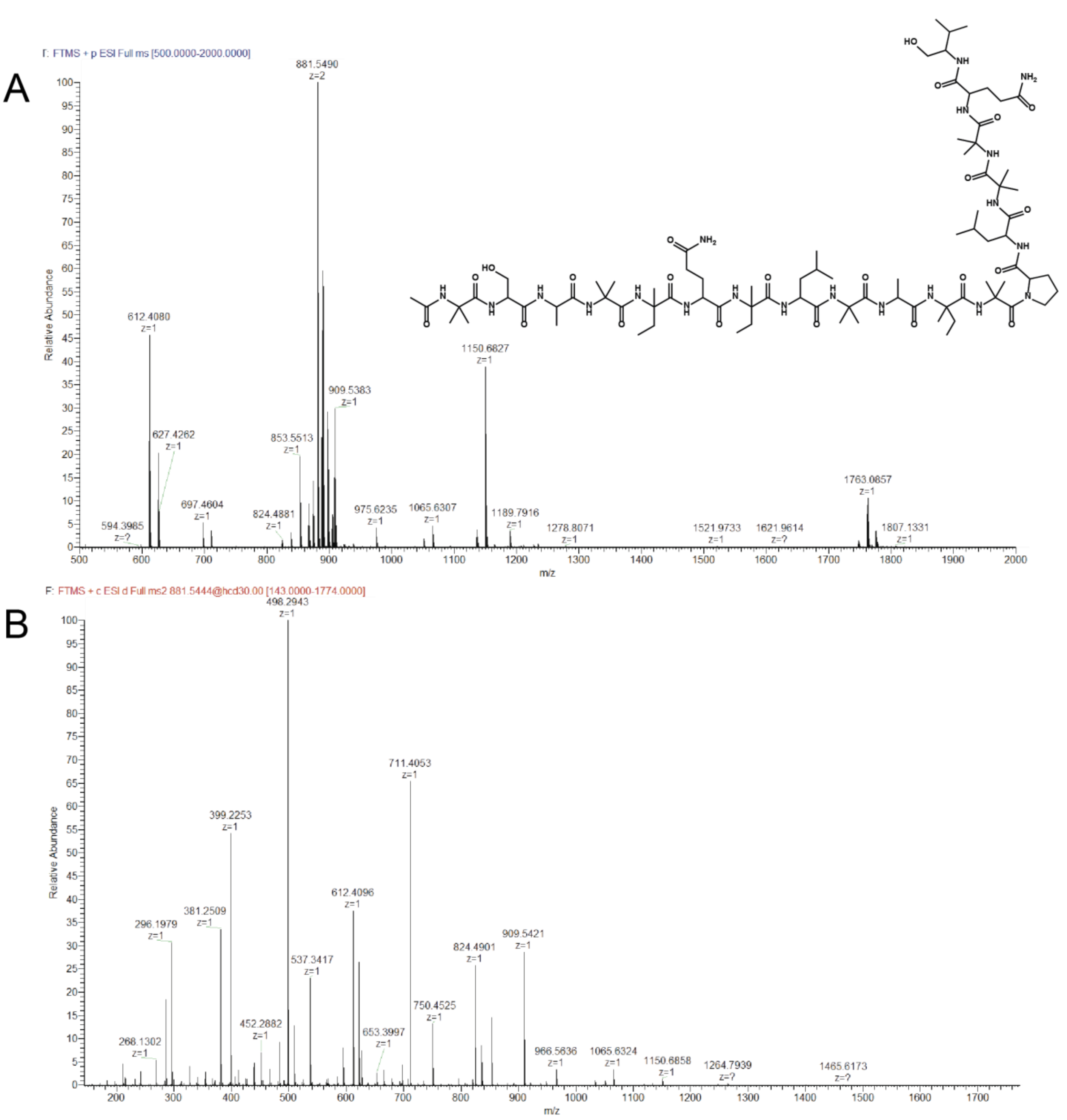

Figure S5. (A) Positive ion mode MS^1 spectrum of 18-residue trichokindin-type peptaibol **3** with the sequence Ac-Aib-Ser-Ala-Aib-Iva-Gln-Iva-Leu-Aib-Ala-Iva-Aib-Pro-Leu-Aib-Aib-Gln-Valol ; (B) HCD mass spectra of for parent ion $[M+2H]^{2+}$ at *m/z* 881.5529.

Figure S6. MS data for major 18-residue peptaibol **4**

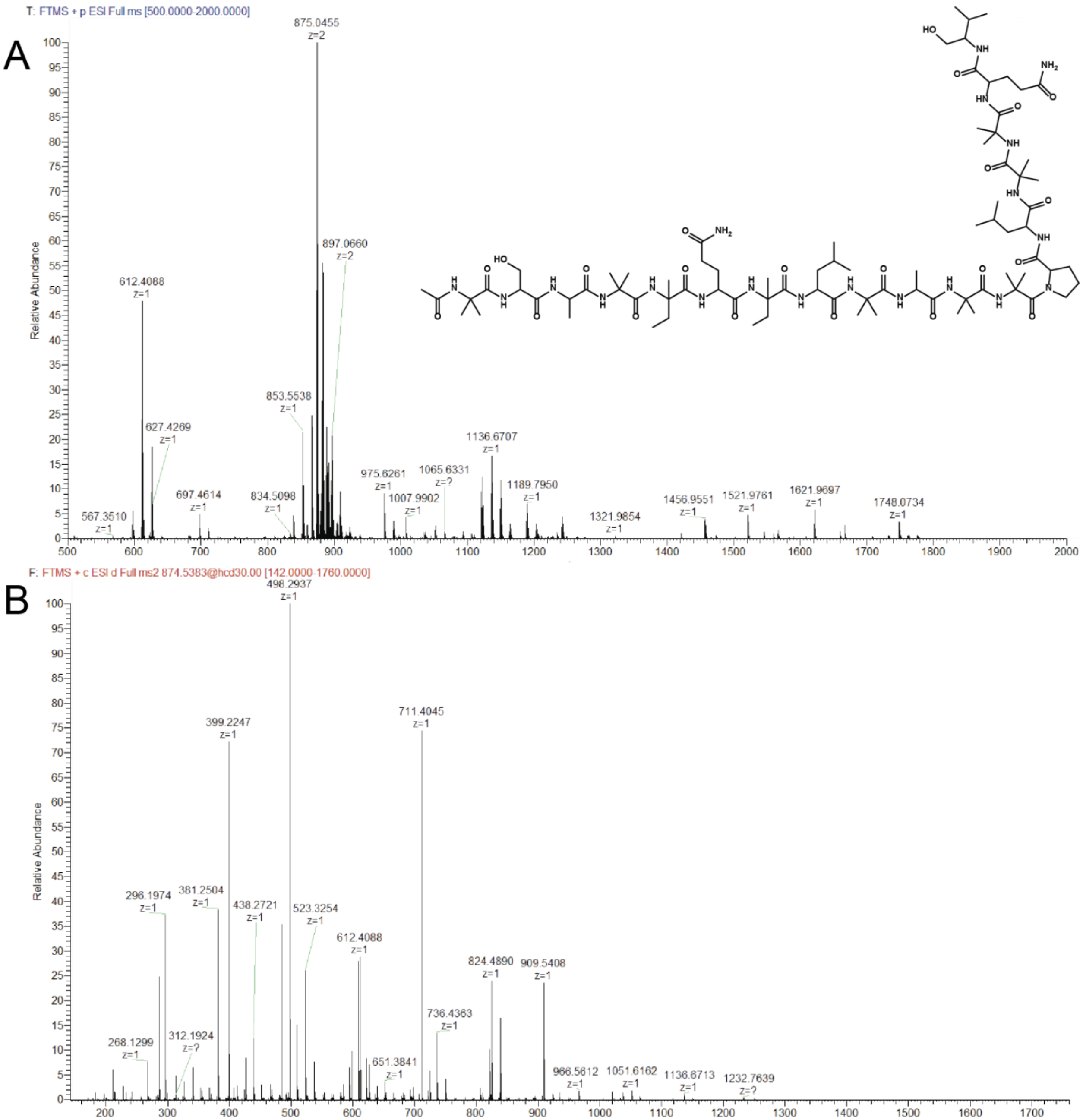

Figure S6. (A) Positive ion mode MS[1] spectrum of of 18-residue trichokindin-type peptaibol **4** with the sequence Ac-Aib-Ser-Ala-Aib-Iva-Gln-Iva-Leu-Aib-Ala-Aib-Aib-Pro-Leu-Aib-Aib-Gln-Valol ; (B) HCD mass spectra of for parent ion $[M+2H]^{2+}$ at *m/z* 874.5435.

Figure S7. MS-fragments assignment for trichokindin-type peptaibols.

	1	2	3	4	5	6	7	8	9	10	11	12	13	14	15	16	17	18
Ac	Aib	Ser	Ala	Aib	Iva	Gln	Iva	Leu	Aib	Ala	Iva	Aib	Pro	Leu	Aib	Aib	Gln	Valol

**Identity**	**Sequence**	**Measured, u**	**Theoretical, u**	**Accuracy, ppm**
2+	TK-type **3**	881.5488	881.5455	3.7
1+	TK-type **3**	1762.0841	1762.0837	0.2
y2	Gln-Valol	232.1658	232.1656	0.9
y3	Aib-Gln-Valol	317.2194	317.2183	3.5
y4	Aib-Aib-Gln-Valol	402.2720	402.2711	2.2
y5	Leu-Aib-Aib-Gln-Valol	515.3522	515.3542	3.9
y6	Pro-Leu-Aib-Aib-Gln-Valol	612.4083	612.4079	0.7
y7	Aib-Pro-Leu-Aib-Aib-Gln-Valol	697.4621	697.4607	2.0
y9	Ala-Iva-Aib-Pro-Leu-Aib-Aib-Gln-Valol	867.5634	867.5662	3.2
y10	Aib-Ala-Iva-Aib-Pro-Leu-Aib-Aib-Gln-Valol	952.6206	952.6190	1.7
b2	Ac-Aib-Ser	215.1042	215.1032	4.6
b3	Ac-Aib-Ser-Ala	286.1401	286.1403	0.7
b5	Ac-Aib-Ser-Ala-Aib-Iva	470.2623	470.2615	1.7
b6	Ac-Aib-Ser-Ala-Aib-Iva-Gln	598.3231	598.3201	5.0
b7	Ac-Aib-Ser-Ala-Aib-Iva-Gln-Iva	697.389	697.3885	0.7
b8	Ac-Aib-Ser-Ala-Aib-Iva-Gln-Iva-Leu	810.4719	810.4725	0.7
b9	Ac-Aib-Ser-Ala-Aib-Iva-Gln-Iva-Leu-Aib	895.5214	895.5253	4.4
b10	Ac-Aib-Ser-Ala-Aib-Iva-Gln-Iva-Leu-Aib-Ala	966.5610	966.5624	1.4
b11	Ac-Aib-Ser-Ala-Aib-Iva-Gln-Iva-Leu-Aib-Ala-Iva	1065.6335	1065.6308	2.5
b12	Ac-Aib-Ser-Ala-Aib-Iva-Gln-Iva-Leu-Aib-Ala-Iva-Aib	1150.6808	1150.6836	2.4
b13	Ac-Aib-Ser-Ala-Aib-Iva-Gln-Iva-Leu-Aib-Ala-Iva-Aib-Pro	1247.7377	1247.7363	1.1

Measured and theoretical m/z values for precursor and fragment ions of the TK-type **3**. **Red** color marks the amino acid residue that varies across the TK-type peptaibols identified in *Trichodema* sp. SK1-7

	1	2	3	4	5	6	7	8	9	10	11	12	13	14	15	16	17	18
Ac	Aib	Ser	Ala	Aib	Iva	Gln	Iva	Leu	Aib	Ala	Aib	Aib	Pro	Leu	Aib	Aib	Gln	Valol

**Identity**	**Sequence**	**Measured, u**	**Theoretical, u**	**Accuracy, ppm**
2+	TK-type **4**	874.5400	874.5377	2.6
1+	TK-type **4**	1748.0684	1748.0681	0.2
y2	Gln-Valol	232.1665	232.1656	3.9
y3	Aib-Gln-Valol	317.2198	317.2183	4.7
y4	Aib-Aib-Gln-Valol	402.2719	402.2711	2.0
y5	Leu-Aib-Aib-Gln-Valol	515.3569	515.3552	3.3
y6	Pro-Leu-Aib-Aib-Gln-Valol	612.4086	612.4079	1.1
y7	Aib-Pro-Leu-Aib-Aib-Gln-Valol	697.4633	697.4607	3.7
y9	Ala-Aib-Aib-Pro-Leu-Aib-Aib-Gln-Valol	853.5538	853.5506	3.7
y10	Aib-Ala-Aib-Aib-Pro-Leu-Aib-Aib-Gln-Valol	938.6072	938.6033	4.2
b2	Ac-Aib-Ser	215.1045	215.1032	6.0
b3	Ac-Aib-Ser-Ala	286.1402	286.1403	0.3
b7	Ac-Aib-Ser-Ala-Aib-Iva-Gln-Iva	697.3865	697.3885	2.9
b8	Ac-Aib-Ser-Ala-Aib-Iva-Gln-Iva-Leu	810.4701	810.4725	3.0
b9	Ac-Aib-Ser-Ala-Aib-Iva-Gln-Iva-Leu-Aib	895.5255	895.5253	0.2
b10	Ac-Aib-Ser-Ala-Aib-Iva-Gln-Iva-Leu-Aib-Ala	966.5594	966.5624	3.1
b11	Ac-Aib-Ser-Ala-Aib-Iva-Gln-Iva-Leu-Aib-Ala-Aib	1051.6157	1051.6152	0.5
b12	Ac-Aib-Ser-Ala-Aib-Iva-Gln-Iva-Leu-Aib-Ala-Aib-Aib	1136.6625	1136.6679	4.8

Measured and theoretical m/z values for precursor and fragment ions of the TK-type **4**. **Red** color marks the amino acid residue that varies across the TK-type peptaibols identified in *Trichodema* sp. SK1-7

Table S4. Annotation of *tho1* BGC

Gene name	Strand	Gene size (bp)	Protein [Organism]	Accession Number	Query cover, %	Protein identity, %
Orf1	-	1701	Apoptosis-inducing factor 2 [Trichoderma lentiforme]	KAF3068030.1	85	95
Orf2	-	726	hypothetical protein M431DRAFT_114494 [Trichoderma harzianum CBS 226.95]	XP_024775658.1	100	99
Orf3	+	1518	hypothetical protein CFAM422_007938 [Trichoderma lentiforme]	KAF3068244.1	100	99
Orf4	-	1500	hypothetical protein Trihar35433_3146 [Trichoderma harzianum]	KAK4073672.1	100	100
Orf5	-	1047	zinc-binding dehydrogenase domain-containing protein [Trichoderma breve]	XP_056030782.1	100	100
Orf6	+	375	hypothetical protein THARTR1_00290 [Trichoderma harzianum]	PNP60266.1	31	90
Orf7	+	915	hypothetical protein Trihar35433_3144 [Trichoderma harzianum]	KAK4073670.1	100	99
Tho1	+	49716	NRPS protein [Trichoderma simmonsii]	QYS95804.1	100	93
Orf9	-	882	Short chain dehydrogenase/reductase [Trichoderma simmonsii]	QYS95802.1	100	99
Orf10	-	1332	Anhydro-N-acetylmuramic acid kinase [Trichoderma lentiforme]	KAF3068247.1	100	99
Orf11	+	1638	hypothetical protein M431DRAFT_82789 [Trichoderma harzianum CBS 226.95]	XP_024775669.1	100	100
Orf12	-	1830	hypothetical protein N5P37_002609 [Trichoderma harzianum]	KAK0765131.1	100	95
Orf13	-	1059	hypothetical protein THAR02_00947 [Trichoderma harzianum]	KKP06967.1	100	97
Orf14	-	210	no hit	-	-	

Table S5. Annotation of *Tho2* BGC

Gene name	Strand	Gene size (bp)	Protein [Organism]	Accession Number	Query cover, %	Protein identity, %
Orf1	-	510	beta carbonic anhydrase clade D [Trichoderma guizhouense]	KAF3068030.1	85	95
Orf2	-	1638	hypothetical protein M431DRAFT_512491 [Trichoderma harzianum CBS 226.95]	XP_024775658.1	100	99
Orf3	+	1890	Retrograde regulation protein 2 [Trichoderma lentiforme]	KAF3068244.1	100	99
Tho2	-	62007	hypothetical protein Trihar35433_5584 [Trichoderma harzianum]	KAK4073672.1	100	100

Table S6. Sequences used for comparative analysis of *tho1* and *tho2* BGCs

Strain	Complete genome sequence accession number, reference for genome analysis	Number of adenylation domains in peptaibol synthase	Protein accession number	Metabolite analysis
Trichoderma lentiforme CFAM-422	GCA_011066345.1	14	KAF3068249.1	[1]
		18	KAF3066167.1	
Trichoderma virens Gv29-8	JGI project 1184795<br>[2]	14	XP_013957420.1	[1]
		18	XP_013953110.1	
Trichoderma sp. IMV00454	GCA_001931985.1	14		
		18		
Trichoderma simmonsii GH-Sj1	GCA_019565615.1	14	QYS95804.1	
		18	QYT04456.1	
Trichoderma gracile HK011-1	GCA_020002365.1	11	KAH0492734.1	
		20	KAH0491796.1	
Trichoderma arundinaceum IBT40837	[3]	11	RFU79003.1	
		20	RFU80400.1	
Trichoderma atroviride	https://mycocosm.jgi.doe.gov/Triat2/Triat2.home.html<br>[2]	14		[4]
		19		
Trichoderma citrinoviride TUCIM6016	JGI project 1013721<br>[5]	11	XP_024752339.1	
		20	XP_024748081.1	[6]
Trichoderma gamsii T6085	[7]	10	XP_024405290.1	
		19	XP_024405762.1	[8]
	[5]	14		

Trichoderma guizhouese NJAU4742		20		
Trichoderma harzianum CBS226.95	JGI project 403727 [5]	12	XP_024775665.1	[9–12]
		18	XP_024769971.1	
Trichoderma koningii JCM1883	GCA_001950475.1	12		
		20		[13]
Trichoderma lixii MUT3171	GCA_014468695.1	12		
		18		
Trichoderma longibrachiatum ATCC18648	JGI project 403728 [5]	12	PTB77615.1	
		20	PTB76898.1	[14–16]
Trichoderma parareesei CBS125925	GCA_001050175.1	14	OTA02976.1	
		20	OTA01063.1	
Trichoderma reesei QM6a	https://mycocosm.jgi.doe.gov/Trire_Chr/Trire_Chr.home.html [17,18]	14	XP_006968900.1	[4]
		20	XP_006968566.1	[19]

Figure S7. Comparison of long peptaibol-synthase contacting clusters in related *Trichoderma*

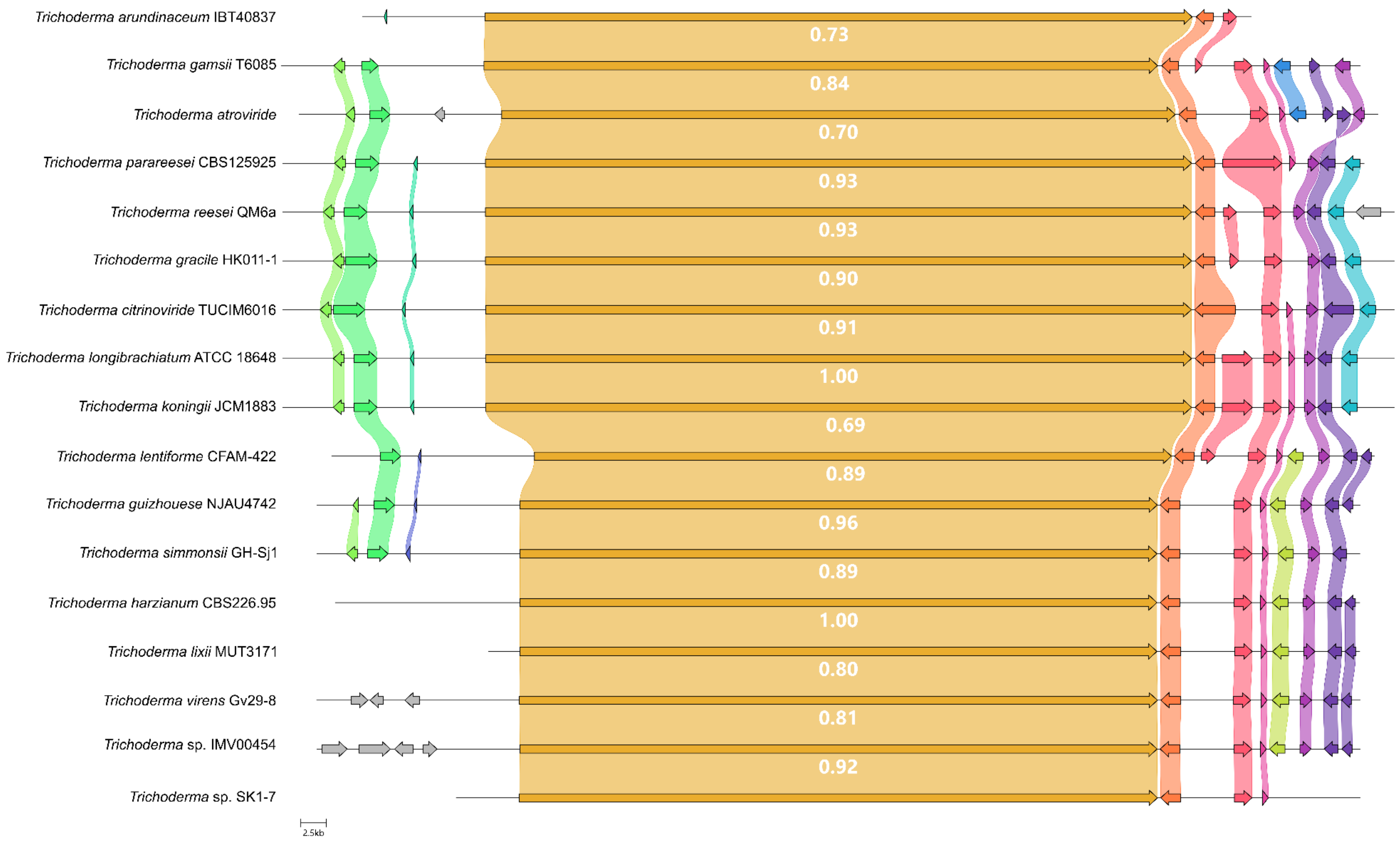

Table S7. Substrate specificity analysis of adenylation domains

Strain		Module number													
		1	2	3	4	5	6	7	8	9	10	11	12	13	14
T._parareesei CBS 125925	Code	DMGYLAGVFK	DVLCAGAVNK	DAFFTGVVIK	DLGYLCGVFK	DVLFCGLICK	DPTLVTGVIK	DAFFVGAVIK	DLGYLAGCFK	DVLFCGLICK	DMAFLGAVIK	DYSFAGVVMK	DLGFLAGVFK	DVLFCGLICK	DAFLLGAVVK
	Score	0.622	0.091	0.275	0.6	0.604	0.127	0.362	0.895	0.604	0.212	0.168	0.971	0.604	0.378
	Substr.	Aib	Trp	Leu	Aib	Pro	Ala	Leu	Aib	Pro	Aib	Aib	Aib	Pro	Leu
	C-domain	HHxxxDG	HHxxxDG	HHxxxDG	HHxxxDG	HHxxxDG	HHxxxDG	HHxxxDG	HHxxxDG	HHxxxDG	HHxxxDG	HHxxxDG	HHxxxDG	HHxxxDG	HHxxxDG
T. reesei QM6a	Code	DMGYLAGVFK	DVLCAGAVNK	DAFFTGVVIK	DLGYLCGVFK	DVLFCGLICK	DPTLVTGVIK	DAFFLGAVIK	DLGYLAGCFK	DVLFCGLICK	DMGFLGAVIK	DYSFAGAVMK	DLGFLAGVFK	DVLFCGLICK	DALLIGAVAK
	Score	0.598	0.081	0.201	0.76	0.604	0.127	0.326	0.895	0.64	0.246	0.14	0.974	0.604	0.391
	Substr.	Aib	Trp	Leu	Aib	Pro	Ala	Leu	Aib	Pro	Aib	Leu	Aib	Pro	Leu
	C-domain	HHxxxDG	HHxxxDG	HHxxxDG	HHxxxDG	HHxxxDG	HHxxxDG	HHxxxDG	HHxxxDG	HHxxxDG	HHxxxDG	HHxxxDG	HHxxxDG	HHxxxDG	HHxxxDG
T. citrinoviride TUCIM6016	Code	DLGFLCGVFK	DVLCAGAVNK	DAFFTGAVIK				DAFFVGAVTK	DLGYLAGCFK	DVLFCGLICK	DMGFLGAVIK	DYSFSGPVMK	DLGFLAGVFK	DVLFCGLICK	DAFLLGAVAK
	Score	0.784	0.082	0.359				0.249	0.895	0.64	0.224	0.17	0.974	0.604	0.349
	Substr.	Aib	Leu	Leu				Leu	Aib	Pro	Aib	Aib	Aib	Pro	Leu
	C-domain	HHxxxDG	HHxxxDG	HHxxxDG	HHxxxDG			**HHxxxDA**	HHxxxDG	HHxxxDG	HHxxxDG	HHxxxDG	HHxxxDG	HHxxxDG	HHxxxDG
T. gracile HK011-1	Code	DMGYLAGVFK	DVLCAGAVNK	DAFFAGAVFK				DAFFVGAVIK	DLGYLAGCFK	DVLFCGLICK	DMGFLGAVIK	DYSFAGAVMK	DLGFLAGVFK	DVLFCGLICK	DAFLLGVVLK
	Score	0.725	0.091	0.281				0.362	0.895	0.64	0.246	0.182	0.974	0.604	0.323
	Substr.	Aib	Trp	Leu				Leu	Aib	Pro	Aib	Leu	Aib	Pro	Leu
	C-domain	HHxxxDG	HHxxxDG	HHxxxDG	HHxxxDG			**HHxxxDA**	HHxxxDG	HHxxxDG	HHxxxDG	HHxxxDG	HHxxxDG	HHxxxDG	HHxxxDG
T. longibrachiatum ATCC18648	Code	DMGYLAGVFK	DVLAAGAVNK	DAFFAGVVIK	DLGYLCGVGK			DAFFVGAVIK	DLGYLAGCFK	DVLFCGLICK	DMCFFGAVVK	DFSFAGAVMK	DLGFVAGVFK	DVLFCGLICK	DAFLLGAVVK
	Score	0.622	0.091	0.299	0.14			0.353	0.895	0.609	0.189	0.178	0.902	0.604	0.453
	Substr.	Aib	Trp	Leu	Aib			Leu	Aib	Pro	Leu	Aib	Aib	Pro	Leu
	C-domain	HHxxxDG	HHxxxDG	HHxxxDG	HHxxxDG			**HHxxxDA**	HHxxxDG	HHxxxDG	HHxxxDG	HHxxxDG	HHxxxDG	HHxxxDG	HHxxxDG
T. koningii JCM1883	Code	DMGYLAGVFK	DVLAAGAVNK	DAFFAGVVIK	DLGYLCGVGK			DAFFVGAVIK	DLGYLAGCFK	DVLFCGLICK	DMCFFGAVVK	DFSFAGAVMK	DLGFVAGVFK	DVLFCGLICK	DAFLLGAVVK
	Score	0,622	0,091	0,299	0,14			0,353	0,895	0,609	0,189	0,178	0,902	0,604	0,453
	Substr.	Aib	Trp	Leu	Aib			Leu	Aib	Pro	Leu	Aib	Aib	Pro	Leu
	C-domain	HHxxxDG	HHxxxDG	HHxxxDG	HHxxxDG			HYxxxDA	HHxxxDG	HHxxxDG	HHxxxDG	HHxxxDG	HHxxxDG	HHxxxDG	HHxxxDG
T. arundinaceum IBT40837	Code	DLGYLCGVFK	DVLSTAAVNK	DAFFVGGVFK				DAFFVGAVIK	DLGYLAGCFK	DVLFCGLICK	DGGLIGAMAK	IVRGLGAVMK	DLGFLAGVFK	DVLFCGLICK	DALLLGVVLK
	Score	0,8	0,106	0,343				0,353	0,895	0,609	0,285	N/A	0,947	0,604	0,365
	Substr.	Aib	Ala	Val				Leu	Aib	Pro	Leu	N/A	Aib	Pro	Leu
	C-domain	HHxxxDG	HHxxxDG	HHxxxDG				HHxxxDG	HHxxxDG	HHxxxDG	HHxxxDG	HHxxxDG	HHxxxDG	HHxxxDG	HHxxxDG
Trichoderma sp. SK1-7	Code	DMGYLAGVFK	DVLCCGAVDK	DAFFTGVVIK	DLGYLCGVFK	DVLFCGLICK	DVGYAMSVIK	DALFVGAVAK	DLGYLAGCFK	DVLFCGLICK	DMGFLGAVIK	DFSFAGCVMK	DLGYLAGVFK	DVLFCGLICK	DAFIFGAITK
	Score	0,668	0,12	0,289	0,625	0,64	0,149	0,47	0,792	0,186	0,159	0,147	0,976	0,604	0,233
	Substr.	Aib	Leu	Leu	Aib	Pro	Ala	Leu	Aib	Pro	Trp	Leu	Aib	Pro	Leu
	C-domain	HHxxxDG	HHxxxDG	**HHxxxDA**	HHxxxDG	HHxxxDG	HHxxxDG	HHxxxDG	HHxxxDG	HHxxxDG	HHxxxDG	HHxxxDG	HHxxxDG	HHxxxDG	HHxxxDG
T. sp. IMV00454	Code	DMGYLAGVFK	DVLCCGAVDK	DAFFTGVVIK	DLGYLCGVFK	DVLFCGLICK	DVGYAMSVIK	DAFFVGAVIK	DLGYLAGCFK	DVLFCGLICK	DMLYLGAIVK	DFSFAGCVMK	DLGYLAGVFK	DVLFCGLICK	DAFIFCAIVK
	Score	0,695	0,12	0,292	0,526	0,64	0,149	0,453	0,895	0,64	0,288	0,147	0,976	0,604	0,176
	Substr.	Aib	Leu	Leu	Aib	Pro	Ala	Leu	Aib	Pro	Leu	Leu	Aib	Pro	Leu
	C-domain	HHxxxDG	HHxxxDG	**HHxxxDA**	HHxxxDG	HHxxxDG	HHxxxDG	HHxxxDG	HHxxxDG	HHxxxDG	HHxxxDG	HHxxxDG	HHxxxDG	HHxxxDG	HHxxxDG
T._virens Gv29-8	Code	DMGFIAGVLK	DVLCCGAVDK	DAFFAGVVIK	DLGYLCGVFK	DVLFCGLICK	DVGFAMSVIK	DAFFIGGVIK	DLGYLAGCFK	DVLFCGLICK	DMGFLGAVIK	DFSFAGCVMK	DLGYLAGVFK	DVLFCGLICK	DAFILCAVMK
	Score	0,487	0,111	0,278	0,651	0,604	0,151	0,268	0,895	0,64	0,164	0,133	0,982	0,64	0,169
	Substr.	Aib	Leu	Leu	Aib	Pro	Ala	Leu	Aib	Pro	Trp	Leu	Aib	Pro	Leu
	C-domain	HHxxxDG	HHxxxDG	HHxxxDG	HHxxxDG	HHxxxDG	HHxxxDG	HHxxxDG	HHxxxDG	HHxxxDG	HHxxxDG	HHxxxDG	HHxxxDG	HHxxxDG	HHxxxDG
T. guizhouese NJAU4742	Code	DMGYLAGVFK	DVLMCGAVDK	DAFTAGAIIK	DLGYLCGVFK	DVLFCGLICK	DVGYAMSIFK	DAFFVGGVIK	DLGYLAGCFK	DVLFCGLICK	DMGFLAGFVK	DFGFAGCVIK	DLGYLAGVFK	DVLFCGLICK	DAFIFCGIIK
	Score	0,668	0,128	0,218	0,76	0,64	0,23	0,375	0,895	0,64	0,451	0,175	0,882	0,604	0,149
	Substr.	Aib	Leu	Leu	Aib	Pro	Aib	Leu	Aib	Pro	Aib	Aib	Aib	Pro	Phe
	C-domain	HHxxxDG	HHxxxDG	**HHxxxDA**	HHxxxDG	HHxxxDG	HHxxxDG	HHxxxDG	HHxxxDG	HHxxxDG	HHxxxDG	HHxxxDG	HHxxxDG	HHxxxDG	HHxxxDG
T. lentiforme CFAM-422	Code	DMGYLAGVFK	DVLMCGAVDK	DAFFTGVVIK	DLGCLCGVFK	DVLFCGLICK	DVGYAMSVIK	DAFFVGAVIK	DLGYLAGCFK	DVLFCGLICK	DMLYLGAIVK	DFSFAGCVMK	DLGYLAGVFK	DVLFCGLICK	DAFIFGAITK
	Score	0,668	0,134	0,289	0,652	0,64	0,149	0,449	0,792	0,64	0,288	0,133	0,976	0,604	0,233
	Substr.	Aib	Leu	Leu	Aib	Pro	Ala	Leu	Aib	Pro	Leu	Leu	Aib	Pro	Leu

	C-domain	HHxxxDG	HHxxxDG	**HHxxxDA**	HHxxxDG	HHxxxDG	HHxxxDG	HHxxxDG	HHxxxDG	HHxxxDG	HHxxxDG	HHxxxDG	HHxxxDG	HHxxxDG	HHxxxDG
T. lixii MUT3171	Code	DMGYLAAVFK	DVLMCGSVDK	DAFFTGVVIK	DLGYLCGVFK			DALFVGAVAK	DLGYLAGCFK	DVLFCGLICK	DMGFLGAVIK	DFSFAGCVMK	DLGYLAGVLK	DVLFCGLICK	DAFIFCAIVK
	Score	0,647	0,108	0,289	0,76			0,48	0,895	0,64	0,157	0,133	0,65	0,604	0,177
	Substr.	Aib	Leu	Leu	Aib			Leu	Aib	Pro	Trp	Leu	Aib	Pro	Leu
	C-domain	HHxxxDG	HHxxxDG	**HHxxxDA**	HHxxxDG			HHxxxDG	HHxxxDG	HHxxxDG	HHxxxDG	HHxxxDG	HHxxxDG	HHxxxDG	HHxxxDG
T. harzianum CBS226.95	Code	DMGYLAAVFK	DVLMCGSVDK	DAFFTGVVIK	DLGYLCGVFK			DALFVGAVAK	DLGYLAGCFK	DVLFCGLICK	DMGFLGAVIK	DFSFAGCVMK	DLGYLAGVLK	DVLFCGLICK	DAFIFCAIVK
	Score	0,647	0,108	0,289	0,76			0,48	0,895	0,64	0,157	0,147	0,65	0,604	0,176
	Substr.	Aib	Leu	Leu	Aib			Leu	Aib	Pro	Trp	Leu	Aib	Pro	Leu
	C-domain	HHxxxDG	HHxxxDG	**HHxxxDA**	HHxxxDG			HHxxxDG	HHxxxDG	HHxxxDG	HHxxxDG	HHxxxDG	HHxxxDG	HHxxxDG	HHxxxDG
T. simmonsii GH-Sj1	Code	DMGFLAGVFK	DVLCCGAVDK	DAFFTGVVIK	DLGYLCGVFK	DVLFCGLICK	DVGYAMSVIK	DALFVGAVAK	DLGYLAGCFK	DVLFCGLICK	DMGFLGAVIK	DFSFAGCVMK	DLGYLAGVFK	DVLFCGLICK	DAFIFGAITK
	Score	0,687	0,12	0,292	0,526	0,621	0,149	0,47	0,895	0,64	0,157	0,133	0,976	0,604	0,196
	Substr.	Aib	Leu	Leu	Aib	Pro	Ala	Leu	Aib	Pro	Trp	Leu	Aib	Pro	Leu
	C-domain	HHxxxDG	HHxxxDG	HHxxxDA	HHxxxDG	HHxxxDG	HHxxxDG	HHxxxDG	HHxxxDG	HHxxxDG	HHxxxDG	HHxxxDG	HHxxxDG	HHxxxDG	HHxxxDG
T. gamsii T6085	Code	DMGYVGGVFK	DGIFCALVMK	DAAFIGAVIK	DLGYLCGVFK	DVLFCGLICK		DMSIVGGAAK	DLGYLAGCFK	DVLFCGLICK			DLGFLAGVFK	DVLFCGLICK	
	Score	0,355	0,113	0,212	0,76	0,604		0,156	0,918	0,64			0,944	0,604	
	Substr.	Aib	Leu	Leu	Aib	Pro		Leu	Aib	Pro			Aib	Pro	
	C-domain	HHxxxDG	HHxxxDG	HHxxxDG	HHxxxDG	HHxxxDG		HHxxxDG	HHxxxDG	HHxxxDG			HHxxxDG	HHxxxDG	HHxxxDW
T. atroviride	Code	DLGYLAGVFK	DGVSCALVMK	DAAIVGAVAK	DLGYLCGVFK			DAFFVGAVIK	DLGYLAGCFK	DMGFLGAVIK			DLGILGGVYK	DVLFCGLICK	
	Score	0,796	0,092	0.227	0.764			0,36	0,895	0,174			0,323	0,604	
	Substr.	Aib	Tyr	Leu	Aib			Leu	Aib	Val			Aib	Pro	
	C-domain	HHxxxDG	HHxxxDG	HHxxxDG	HHxxxDG			HHxxxDG	HHxxxDG	HHxxxDG			HHxxxDG	HHxxxDG	HYxxxDE

Table S8. Aib-biosynthesis associated genes analysis

Gene name	AntiSMASH annotation	Gene size (bp)	Protein [Organism]	Accession Number	Query cover, %	Protein identity, %	Known TqaLFM homologues	TqaLFM homologues identity, %
Region 1 (contig 36)								
Orf1	g5979	1717	BZIP domain-containing protein [Trichoderma simmonsii]	QYT00464.1	100	94		
Orf2	g5980	978	Thioesterase domain-containing protein [Trichoderma simmonsii]	QYT00465.1	100	96	-	-
tqaF-th	g5981	762	haloacid dehalogenase-like hydrolase domain-containing protein [Trichoderma breve]	XP_056027360.1	100	98	*tqaF-pa* (ADY16692.1)	45
Orf4	g5982	738	hypothetical protein Trisim1_012127 [Trichoderma cf. simile WF8]	KAL5084327.1	100	98		
			atp synthase alpha chain precursor [Trichoderma arundinaceum]	RFU76195.1	100	74		
tqaL-th	g5983	1170	2OG-Fe dioxygenase domain-containing protein [Trichoderma breve]	XP_056027362.1	100	98	*tqaL-pa* (ADY16694.1)	62
							tqaL-pd (EKV18313.1)	60
							tqaL-ha (XP_013945817.2)	85
Orf6	g5984	7413	Carrier domain-containing protein [Trichoderma simmonsii]	QYT00469.1	100	92		
Orf7	g5985	1797	AMP-binding enzyme domain-containing protein [Trichoderma breve]	XP_056027364.1	98	91		
Region 2								
tqaM-th	g6024	942	hypothetical protein TsFJ059_007072 [Trichoderma semiorbis]	KAH0524578.1	100	99	*tqaM-pa* (ADY16691.1)	71

Figure S8. Sequence similarity network (SSN) analysis of *tqaL-th* homologues

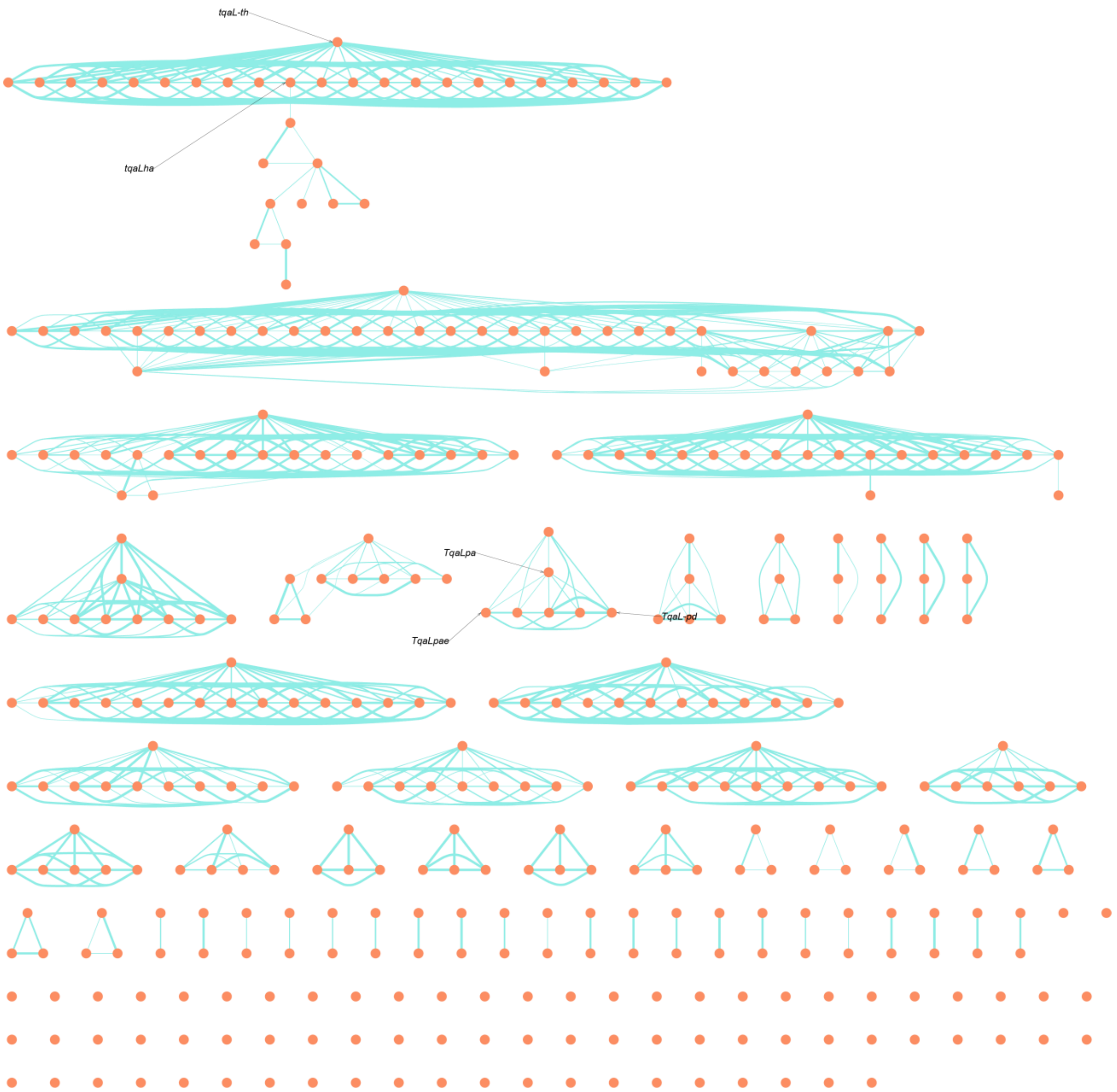

Figure S9. AlphaFold prediction for TqaL-th structure

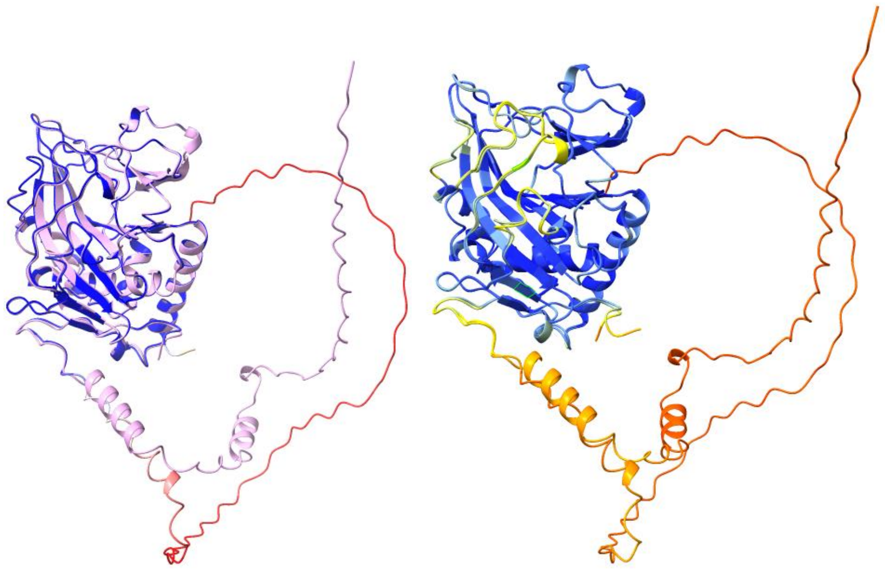

Merge of AlphaFold3 predicted structures of TqaL-*ha(XP_013945817.2) and* TqaL-*th. Left - RMSD(Root mean square deviation) blue - low, red - high, right - AlphaFold PAE(Predicted aligned errors).*

Table S9. Antimicrobial activity of **1** and **2**

**Bacterial strains**	**MIC, µg/mL**	
	**1**	**2**
Micrococcus luteus ATCC 4698	12.5	6.25
Arthrobacter ATCC 21022	12.5	6.25
Bacillus cereus X1	50	25
Bacillus subtilis 168	50	25
Lactococcus lactis 61	>50	>50
Enterococcus faecalis 125	>50	>50
Enterococcus faecium 40	50	>50
Macrococcus caseolyticus 107	12.5	6.25
Staphylococcus aureus GFP	>50	>50
Staphylococcus epidermidis 39	>50	>50
Staphylococcus haemolyticus 515	>50	>50
Escherichia coli ΔtolC	>50	>50
Escherichia coli lptD^{mut}	25	50
Escherichia coli BL21(DE3)	>50	>50
Pseudomonas aeruginosa 51911	>50	>50

Figure S10. Evaluation of cytotoxicity for **1** and **2**.

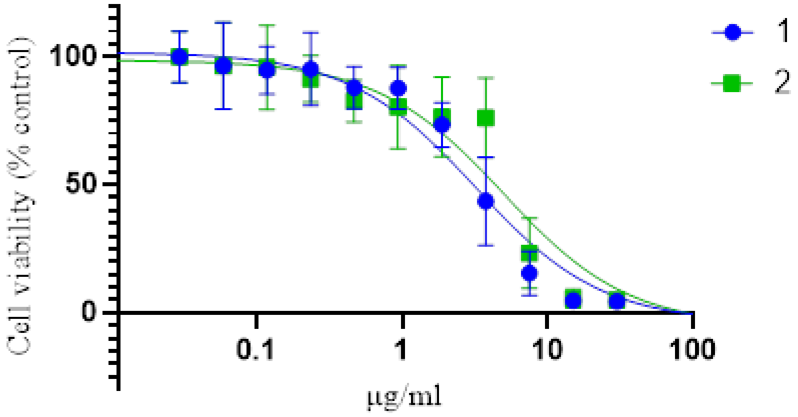

Compound	IC_{50},µg/ml
**1**	3±0,9
**2**	5 ±1,2

References

1. Mukherjee, P.K.; Wiest, A.; Ruiz, N.; Keightley, A.; Moran-Diez, M.E.; McCluskey, K.; Pouchus, Y.F.; Kenerley, C.M. Two Classes of New Peptaibols Are Synthesized by a Single Non-Ribosomal Peptide Synthetase of Trichoderma Virens. *Journal of Biological Chemistry* **2011**, *286*, 4544–4554, doi:10.1074/jbc.M110.159723.
2. Kubicek, C.P.; Herrera-Estrella, A.; Seidl-Seiboth, V.; Martinez, D.A.; Druzhinina, I.S.; Thon, M.; Zeilinger, S.; Casas-Flores, S.; Horwitz, B.A.; Mukherjee, P.K.; et al. Comparative Genome Sequence Analysis Underscores Mycoparasitism as the Ancestral Life Style of Trichoderma. *Genome Biol* **2011**, *12*, R40, doi:10.1186/gb-2011-12-4-r40.
3. Proctor, R.H.; McCormick, S.P.; Kim, H.-S.; Cardoza, R.E.; Stanley, A.M.; Lindo, L.; Kelly, A.; Brown, D.W.; Lee, T.; Vaughan, M.M.; et al. Evolution of Structural Diversity of Trichothecenes, a Family of Toxins Produced by Plant Pathogenic and Entomopathogenic Fungi. *PLoS Pathog* **2018**, *14*, e1006946, doi:10.1371/journal.ppat.1006946.
4. Degenkolb, T.; Karimi Aghcheh, R.; Dieckmann, R.; Neuhof, T.; Baker, S.E.; Druzhinina, I.S.; Kubicek, C.P.; Brückner, H.; von Döhren, H. The Production of Multiple Small Peptaibol Families by Single 14-Module Peptide Synthetases in *Trichoderma* / *Hypocrea*. *Chemistry & Biodiversity* **2012**, *9*, 499–535, doi:10.1002/cbdv.201100212.
5. Druzhinina, I.S.; Chenthamara, K.; Zhang, J.; Atanasova, L.; Yang, D.; Miao, Y.; Rahimi, M.J.; Grujic, M.; Cai, F.; Pourmehdi, S.; et al. Massive Lateral Transfer of Genes Encoding Plant Cell Wall-Degrading Enzymes to the Mycoparasitic Fungus Trichoderma from Its Plant-Associated Hosts. *PLoS Genet* **2018**, *14*, e1007322, doi:10.1371/journal.pgen.1007322.
6. Maddau, L.; Cabras, A.; Franceschini, A.; Linaldeddu, B.T.; Crobu, S.; Roggio, T.; Pagnozzi, D. Occurrence and Characterization of Peptaibols from Trichoderma Citrinoviride, an Endophytic Fungus of Cork Oak, Using Electrospray Ionization Quadrupole Time-of-Flight Mass Spectrometry. *Microbiology* **2009**, *155*, 3371–3381, doi:10.1099/mic.0.030916-0.
7. Baroncelli, R.; Zapparata, A.; Piaggeschi, G.; Sarrocco, S.; Vannacci, G. Draft Whole-Genome Sequence of *Trichoderma Gamsii* T6085, a Promising Biocontrol Agent of *Fusarium* Head Blight on Wheat. *Genome Announc* **2016**, *4*, e01747-15, doi:10.1128/genomeA.01747-15.
8. Marik, T.; Tyagi, C.; Racić, G.; Rakk, D.; Szekeres, A.; Vágvölgyi, C.; Kredics, L. New 19-Residue Peptaibols from Trichoderma Clade Viride. *Microorganisms* **2018**, *6*, 85, doi:10.3390/microorganisms6030085.
9. Niu, X.; Thaochan, N.; Hu, Q. Diversity of Linear Non-Ribosomal Peptide in Biocontrol Fungi. *JoF* **2020**, *6*, 61, doi:10.3390/jof6020061.
10. Peltola, J.; Ritieni, A.; Mikkola, R.; Grigoriev, P.A.; Pócsfalvi, G.; Andersson, M.A.; Salkinoja-Salonen, M.S. Biological Effects of *Trichoderma Harzianum* Peptaibols on Mammalian Cells. *Appl Environ Microbiol* **2004**, *70*, 4996–5004, doi:10.1128/AEM.70.8.4996-5004.2004.
11. Duval, D.; Rebuffat, S.; Goulard, C.; Prigent, Y.; Becchi, M.; Bodo, B. Isolation and Sequence Analysis of the Peptide Antibiotics Trichorzins PA from Trichoderma Harzianum. *J. Chem. Soc., Perkin Trans. 1* **1997**, 2147–2154, doi:10.1039/a700244k.
12. Goulard, C.; Hlimi, S.; Rebuffat, S.; Bodo, B. Trichorzins HA and MA, Antibiotic Peptides from Trichoderma Harzianum. I. Fermentation, Isolation and Biological Properties. *J. Antibiot.* **1995**, *48*, 1248–1253, doi:10.7164/antibiotics.48.1248.
13. Huang, Q.; Tezuka, Y.; Hatanaka, Y.; Kikuchi, T.; Nishi, A.; Tubaki, K. Studies on Metabolites of Mycoparasitic Fungi. IV. Minor Peptaibols of Trichoderma Koningii. *Chem. Pharm. Bull.* **1995**, *43*, 1663–1667, doi:10.1248/cpb.43.1663.

14. Rahimi Tamandegani, P.; Marik, T.; Zafari, D.; Balázs, D.; Vágvölgyi, C.; Szekeres, A.; Kredics, L. Changes in Peptaibol Production of Trichoderma Species during In Vitro Antagonistic Interactions with Fungal Plant Pathogens. *Biomolecules* **2020**, *10*, 730, doi:10.3390/biom10050730.
15. Leclerc, G.; Goulard, C.; Prigent, Y.; Bodo, B.; Wróblewski, H.; Rebuffat, S. Sequences and Antimycoplasmic Properties of Longibrachins LGB II and LGB III, Two Novel 20-Residue Peptaibols from *Trichoderma l Ongibrachiatum*. *J. Nat. Prod.* **2001**, *64*, 164–170, doi:10.1021/np000240s.
16. Mohamed-Benkada, M.; François Pouchus, Y.; Vérité, P.; Pagniez, F.; Caroff, N.; Ruiz, N. Identification and Biological Activities of Long-Chain Peptaibols Produced by a Marine-Derived Strain of *Trichoderma Longibrachiatum*. *Chemistry & Biodiversity* **2016**, *13*, 521–530, doi:10.1002/cbdv.201500159.
17. Martinez, D.; Berka, R.M.; Henrissat, B.; Saloheimo, M.; Arvas, M.; Baker, S.E.; Chapman, J.; Chertkov, O.; Coutinho, P.M.; Cullen, D.; et al. Genome Sequencing and Analysis of the Biomass-Degrading Fungus Trichoderma Reesei (Syn. Hypocrea Jecorina). *Nat Biotechnol* **2008**, *26*, 553–560, doi:10.1038/nbt1403.
18. Li, W.-C.; Huang, C.-H.; Chen, C.-L.; Chuang, Y.-C.; Tung, S.-Y.; Wang, T.-F. Trichoderma Reesei Complete Genome Sequence, Repeat-Induced Point Mutation, and Partitioning of CAZyme Gene Clusters. *Biotechnol Biofuels* **2017**, *10*, 170, doi:10.1186/s13068-017-0825-x.
19. Pócsfalvi, G.; Ritieni, A.; Ferranti, P.; Randazzo, G.; Vékey, K.; Malorni, A. Microheterogeneity Characterization of a Paracelsin Mixture fromTrichoderma Reesei Using High-Energy Collision-Induced Dissociation Tandem Mass Spectrometry. *Rapid Commun. Mass Spectrom.* **1997**, *11*, 922–930, doi:10.1002/(SICI)1097-0231(199705)11:8<922::AID-RCM927>3.0.CO;2-N.
